# Supplementary material for: 20S proteasomes secreted by the malaria parasite promote its growth
Source: Nat Commun. 2021 Feb 19;12:1172. doi: 10.1038/s41467-021-21344-8 (PMC7895969; doi:10.1038/s41467-021-21344-8)
Supplement: Supplementary file 1 — Supplementary Information [file 41467_2021_21344_MOESM1_ESM.pdf]

Supplementary Figure 1

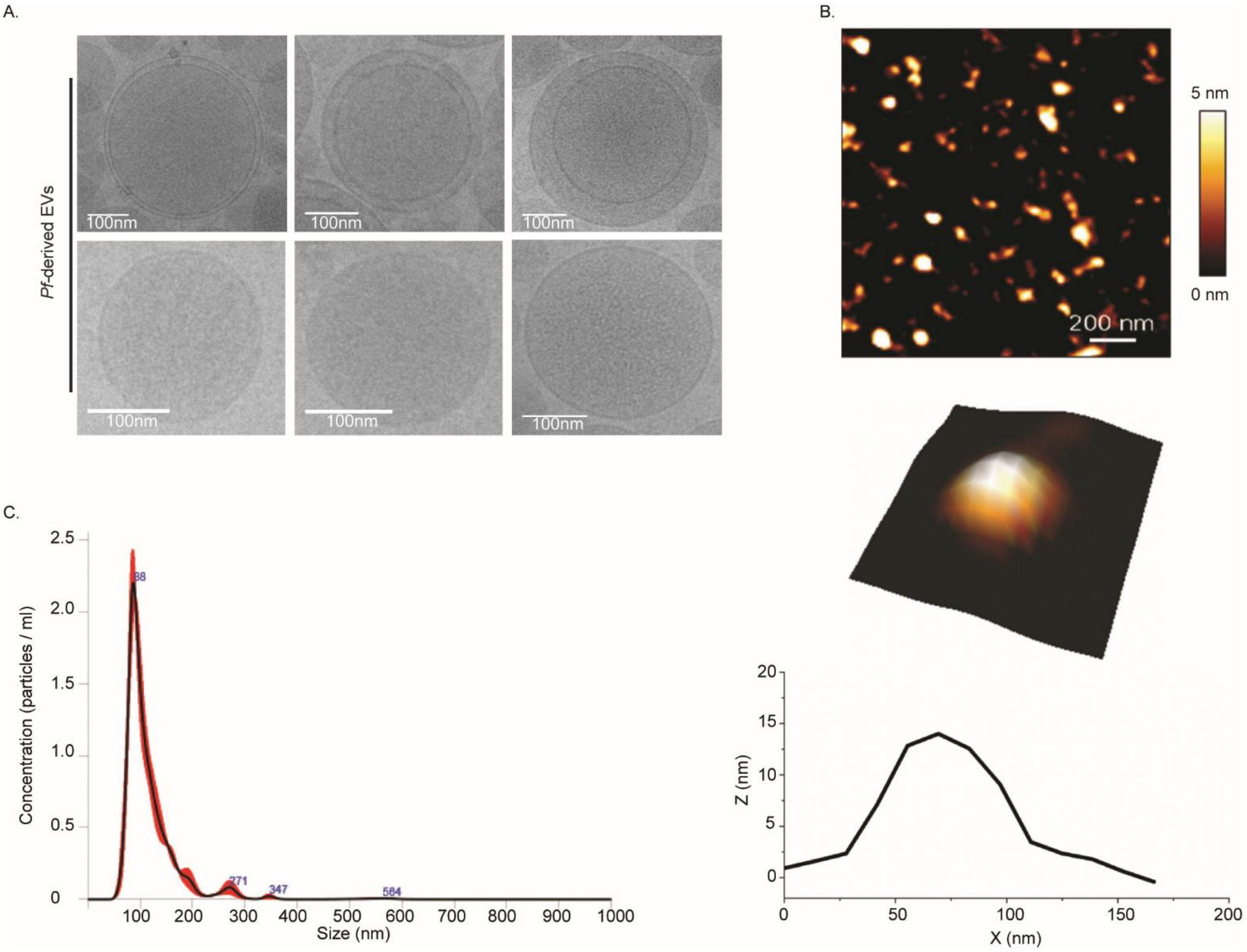

Supplementary Figure 1: Isolation and characterization of *Pf*-derived EVs.
(A) Cryo-EM images of *Pf*-derived EVs harvested from *Pf*-iRBCs. Scale bar is 100 nm. Micrographs were recorded in low-dose Cryo-mode using a bottom-mount Gatan OneView camera. **(B)** AFM images of *Pf*-EVs in PBS, adsorbed on mica modified with Mg^{2+} (incubated for 2 min with 10 mM $MgCl_2$ prior to EVs adsorption). AFM imaging was carried out using a JPK Nanowizard III AFM microscope (Berlin, Germany) in QI mode with a qp-BioAC-CI probe (Nanosensors). **(C)** Nanoparticle tracking analysis (NTA) at 20 °C of EV concentration and size. Sample size distributions were calibrated in a liquid suspension (1:1000 dilution) by the analysis of Brownian motion via light scattering. The camera level was set to 13 and the gain to 1, laser 405 or 488 nm without filter. These experiments were performed independently 3 times with similar results.

Supplementary Figure 2

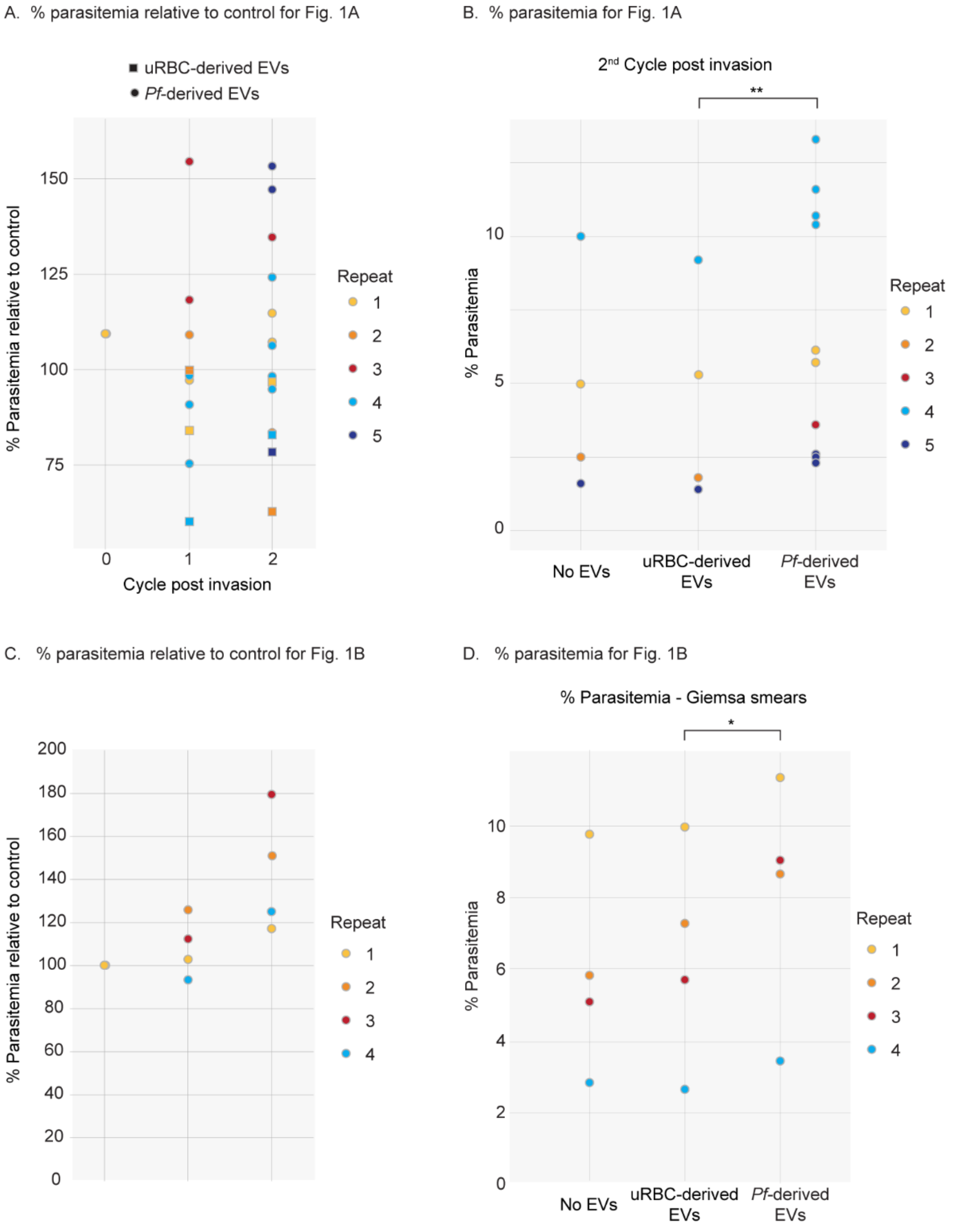

Supplementary Figure 2: Pretreatment with *Pf*-derived EVs increases parasitemia
(A) Raw data for Figure 1A. Squares and circles denote uRBC-derived Evs and *Pf*-derived Evs, respectively. Differentially colored datapoints represent biological repeats. **(B)** Raw data (percentage of parasitemia in the second blood cycle) from five different experiments that were used to generate Figure 1A. Differentially colored dots represent biological repeats. Percentages of parasitemia were compared using a 2-way ANOVA, accounting for treatment and batch, followed by Tukey's post-hoc test. While percentages are different among batches, the differences in parasitemia between uRBC-derived EVs and *Pf*-derived EVs are consistent between batches (**p=0.0026). **(C)** Raw data for Figure 1B. Differentially colored circles represent biological repeats. **(D)** Raw data (percentage of parasitemia, monitored by Giemsa smears), from four different experiments that were used to generate Figure 1B. Differentially colored dots represent biological repeats. Percentages of parasitemia were compared using a 2-way ANOVA, accounting for treatment and batch, followed by Tukey's post-hoc test (*p=0.044).

Supplementary Figure 3

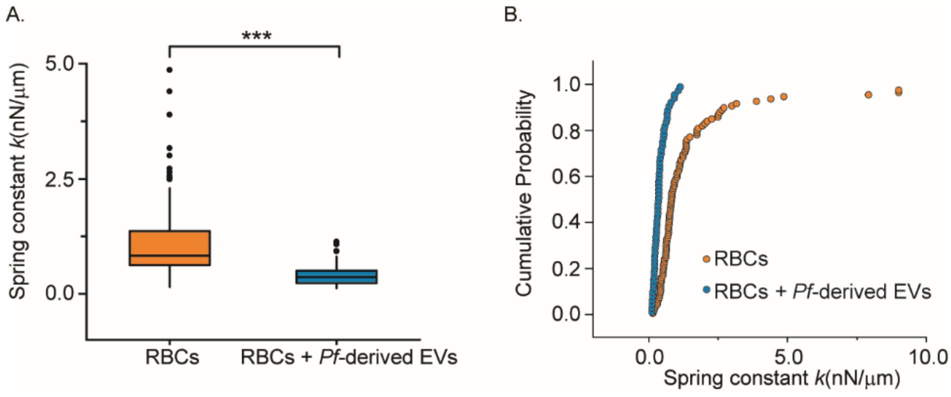

Supplementary Figure 3: The deformability of RBCs is increased after treatment with *Pf*-derived Evs.

Effect of *Pf*-derived EVs on RBCs mechanics as measured via Acoustic Force Spectroscopy in response to force clamps of ~340 pN. (A) Distribution of measured elastic coefficients/spring constants k and (B) cumulative probability distributions for untreated RBCs (N = 103) and cells treated with *Pf*-derived EVs (N = 58). For untreated RBCs the median value of k was found to be 0.83 nN/μm (90% Confidence Interval CI [0.63-1.36]), while for RBCs treated with *Pf*-derived EVs, the spring constant k = 0.36 (90% CI [0.23-0.50]). Box plots in (A) represent the 25% – 75% percentiles of the sample distribution. The black horizontal line represents the median. Black vertical lines represent the outermost data point that falls within the 75th percentile + (1.5 x Interquartile Range) for the upper inner fence and the 25th percentile - (1.5 x Interquartile Range) for the lower inner fence. Dots represent outliers. Statistical significance was assessed via a non-parametric two-sample two-tailed Kolmogorov-Smirnov test in OriginLab Pro 2018b (v. 9.55), ***p=3.78E-13.

Supplementary Figure 4

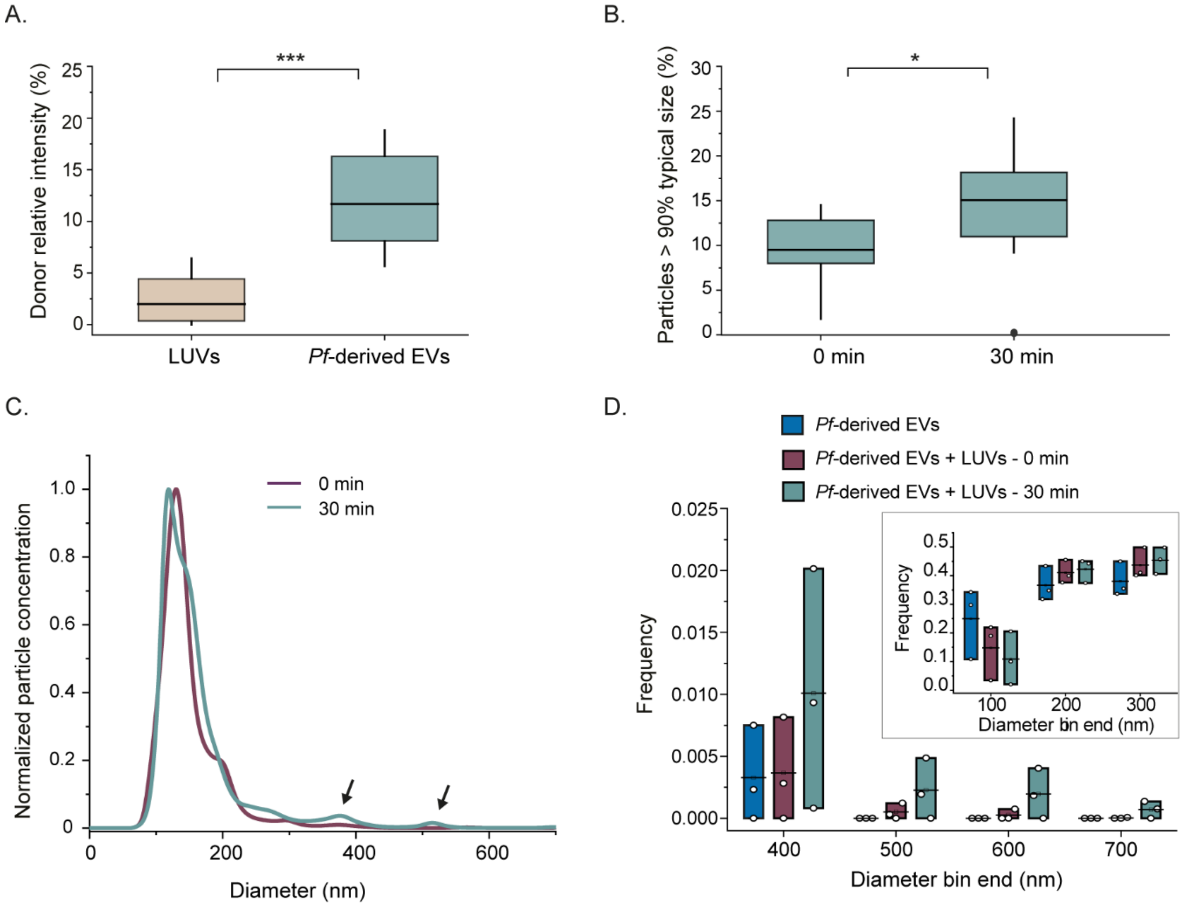

Supplementary Figure 4: *Pf*-derived EVs fuse with liposomes (LUVs) with a composition resembling the RBC membrane

(A) Membrane mixing assay between LUVs mimicking the RBC plasma membrane lipid composition and between *Pf*-derived EVs and LUVs, after incubation at 37 °C, represented as percentage of donor intensity compared to intensity after addition of TRITON X-100. Statistical analysis was done using two-sided t-test under equal variance (***p=3.8E-7). The experiment was performed four biologically independent times. Each biological repeat had three technical repeats. (B) Size distribution measurement of the fraction of particles above the D90, obtained at time 0 for *Pf*-derived EVs and LUVs, immediately after mixing and after 30 min of incubation at 37 °C. Boxes represent the 25 – 75 percentiles of the sample distribution, with black vertical lines representing the remaining percentiles of the distribution. The black dot represents an outlier. Black horizontal line represents the median value of the distribution. Statistical analysis was done using two-sided t-test under equal variance (*p=0.01282). The experiment was performed in three biologically independent times. Each biological repeat had five technical repeats. (C) Size distribution measurement obtained from nanoparticles tracking analysis for *Pf*-derived EVs mixed with RBC plasma membrane-mimicking LUVs immediately after mixing (red) and after 30 min incubation at 37 °C (light blue). Data are representative of the size distribution of one biological sample, averaged from five acquisitions for 60 s. Arrows indicate new diameter populations appearing after 30 min of interaction between EVs and LUVs. (D) Binned size distribution of *Pf*-derived EVs (blue), *Pf*-derived EVs interacting with RBC-mimicking LUVs immediately after mixing (red), and after 30 min of incubation at 37 °C (light blue), showing a statistically significant increase in size upon incubation with LUVs. Main panel shows large (400 – 700 nm) diameter portion of the distribution, where larger size populations appear after 30 min of incubation with LUVs. Inset shows the 0 – 300 nm diameter range, where no significant variation between the samples is observed. Data are represented as average and standard error calculated from 3 biological samples with 5 technical repeats each, using a bin end value of 100 nm. Boxes represent the 25 – 75 percentiles of the sample distribution, with black vertical lines representing the remaining percentiles of the distribution. Black horizontal line represents the mean value of the distribution. Dots represent the actual datapoints.

Supplementary Figure 5

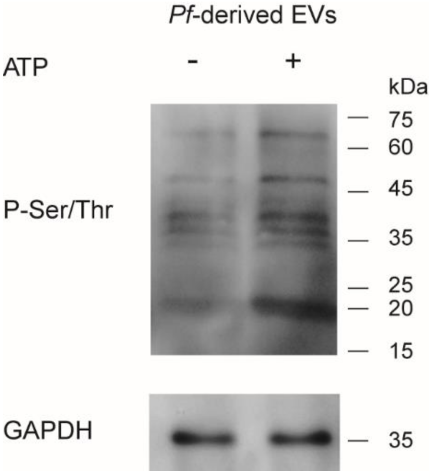

Supplementary Figure 5: The kinases within *Pf*-derived EVs are active.
Western blot analysis of the kinase reaction of *Pf*-derived EVs in the presence (+) or absence (-) of ATP. Protein samples were separated using SDS-PAGE and the changes in phosphorylation events were probed using a phospho-serine/threonine antibody. GAPDH was used as a control. This experiment was performed independently two times with similar results. Source data are provided in the source data file.

Supplementary Figure 6

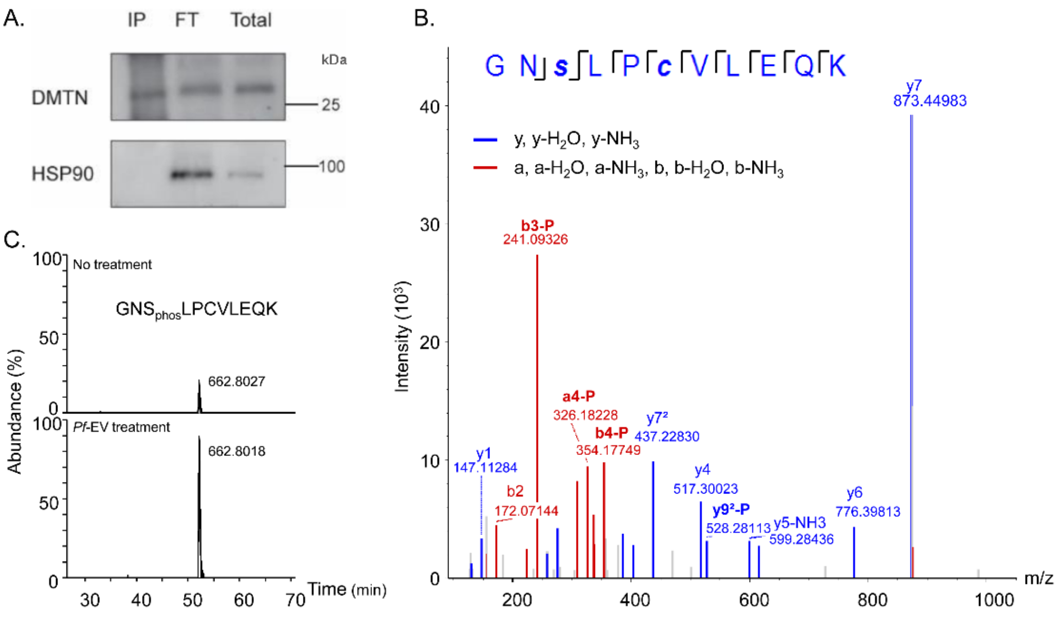

Supplementary Figure 6: Immunoprecipitation assay for dematin upon naïve RBC treatment with *Pf*-derived EVs.

(A) Naïve RBCs were treated with *Pf*-derived EVs, followed by immunoprecipitation (IP) using an anti-dematin (DMTN) antibody from the RBC lysate. Western blot analysis was used for the IP samples separated on the SDS-PAGE gel. FT represents flow through, IP represents dematin-immunoprecipitation sample. HSP90 was used as control. The IP experiments were performed three independent times with similar results. (B) LC-MS/MS spectrum of a doubly charged phosphopeptide ion (662.801 m/z, GNs(phos)LPc(carbmidomethyl)VLEQK) of dematin pull-down following *Pf*-derived EV treatment of uRBCs. The peptide fragmentation pattern indicates a Ser333 phosphorylation site. The *b* and *y* ion fragmentation series are labeled. Fragment ions written in bold indicates the phosphorylated (P) fragments of the peptide. (C) Elution profile of the phospho LC-MS/MS analysis of the same peptide of dematin pull-down following *Pf*-derived EV treatment of uRBCs (bottom panel). As a control untreated uRBC dematin pull-down were analyzed (top panel). The MS experiments were performed two independent times with similar results. Source data are provided in the source data file.

Supplementary Figure 7

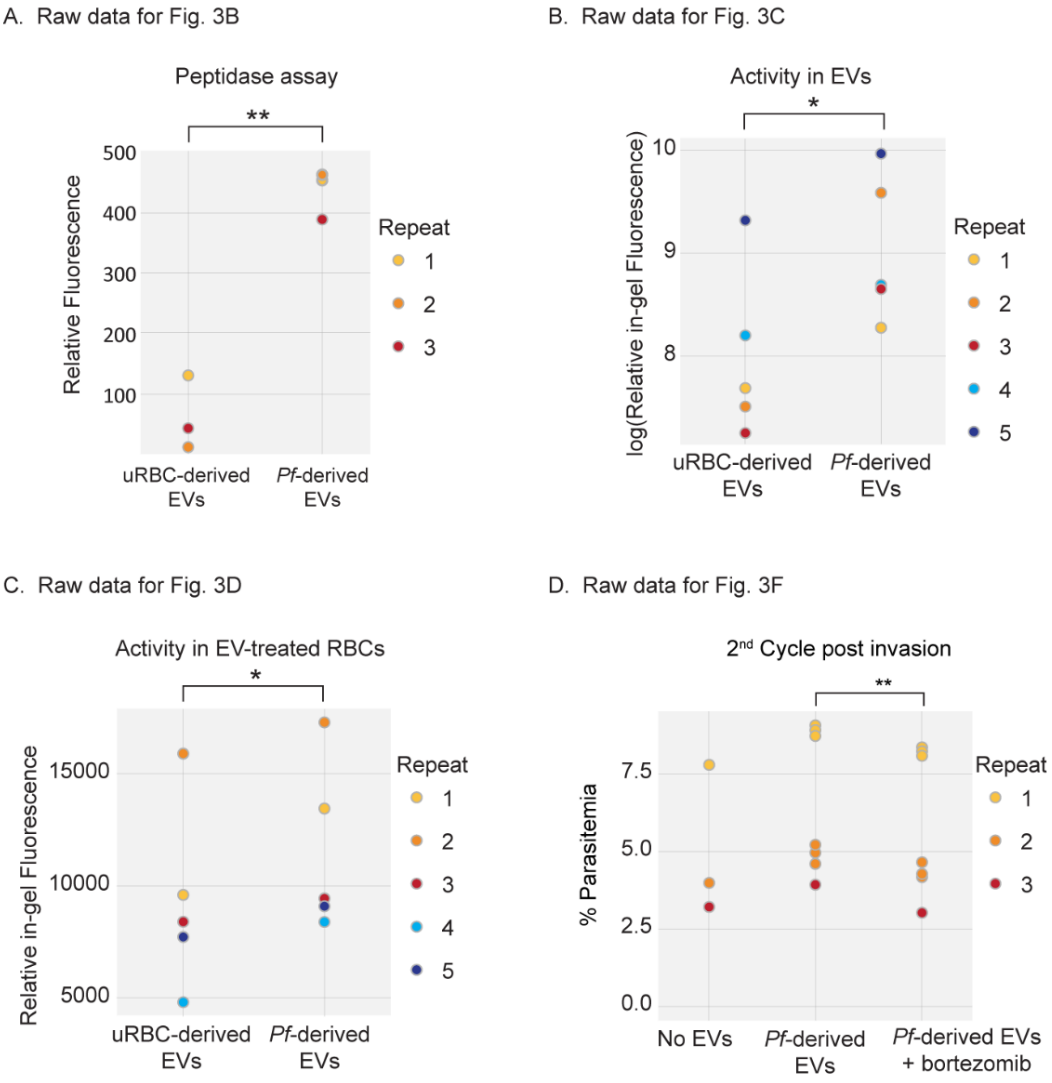

Supplementary Figure 7: Raw data used to generate figures 3B-3D and 3F
(A) Raw data (relative fluorescence) from three different experiments that were used to generate Figure 3B. Differentially colored dots represent biological repeats. Statistical analysis was performed using a two-way two sample t-test assuming unequal variances, (**p=0.0032). (B) Raw data (log of relative in-gel fluorescence), from five different experiments that were used to generate Figure 3C, middle panel. Differentially colored dots represent biological repeats. Biological replicates were subjected to paired t-test analysis using log-transformed measurements (*p=0.028). (C) Raw data (relative in-gel fluorescence), from five different experiments that were used to generate Figure 3D, middle panel. Differentially colored dots represent biological repeats. Biological replicates were subjected to paired t-test analysis (*p=0.021). (D) Raw data (percent parasitemia in the second blood cycle), from three different experiments that were used to generate Figure 3F. Differentially colored dots represent biological repeats. Percentages of parasitemia were compared using a 2-way ANOVA, accounting for treatment and batch, followed by Tukey's post-hoc (***p=0.00027). Source data are provided in the source data file.

Supplementary Figure 8

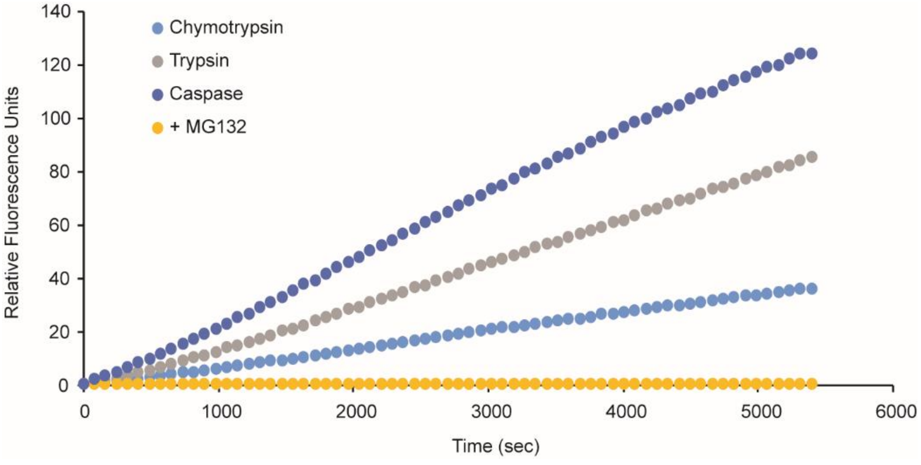

Supplementary Figure 8: Proteolytic activity of *Pf*-derived EV 20S proteasomes.
The three different proteolytic activities of the 20S proteasome were measured using three fluorogenic peptide substrates; suc-LLVY-AMC (for chymotrypsin-like activity), Boc-LRR-AMC (for trypsin-like activity) and Z-LLE-AMC (for caspase-like activity). The proteasome inhibitor, MG132, was used as control.

Supplementary Figure 9

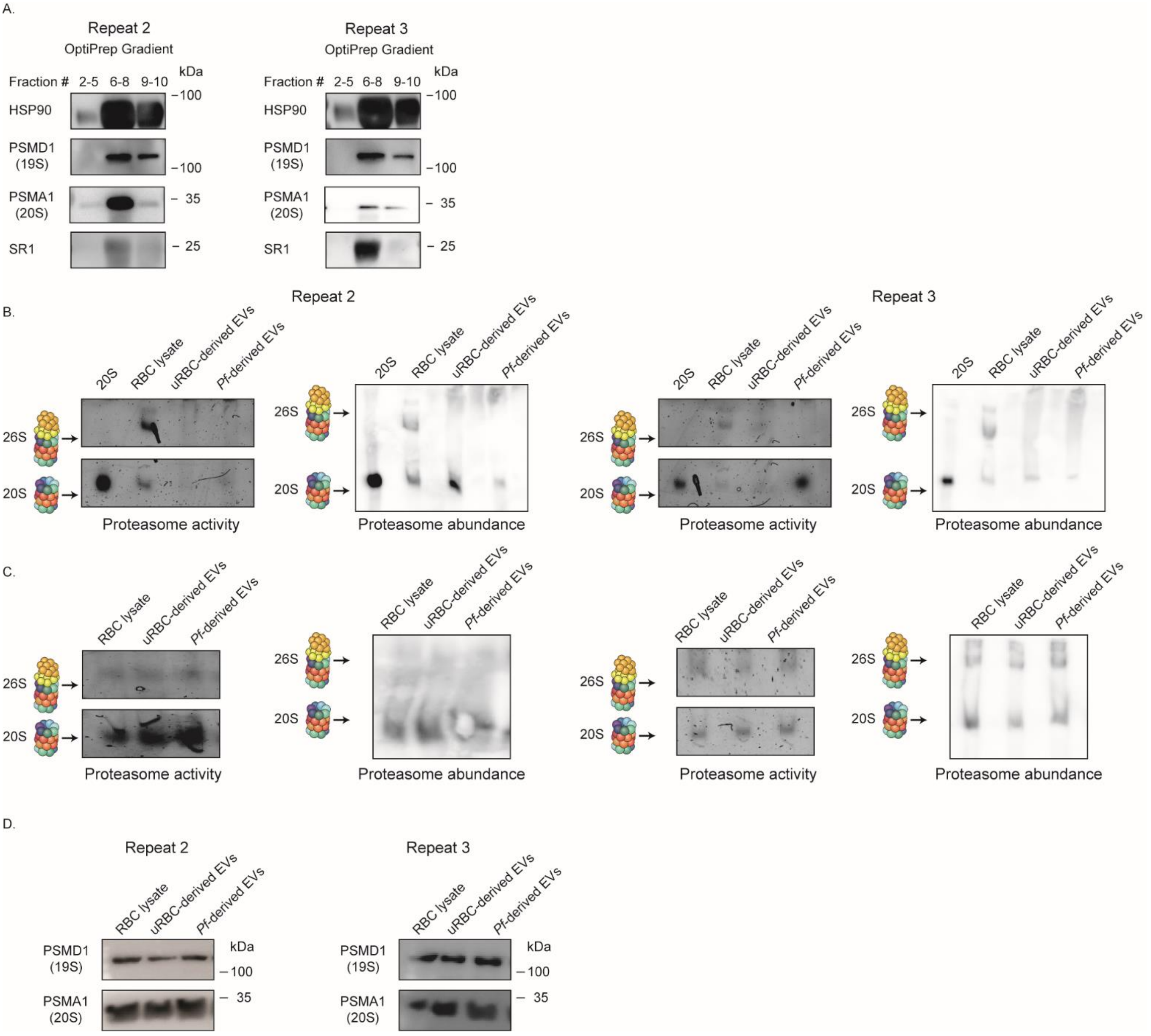

Supplementary Figure 9: The 20S proteasome is assembled and functional within *Pf*-derived EVs.

(A) Two additional repeats of the OptiPrep fractionation results shown in Fig. 3A. (B) Two additional repeats of the *Pf*-derived EV 20S proteasome activity and abundance shown in Fig. 3C. (C) Two additional repeats of the recipient naïve RBC 20S proteasomes' activity and abundance shown in Fig. 3D. (D) Two additional repeats of the naïve RBC denaturative gel shown in Fig. 3E. Source data are provided in the source data file.

Supplementary Figure 10

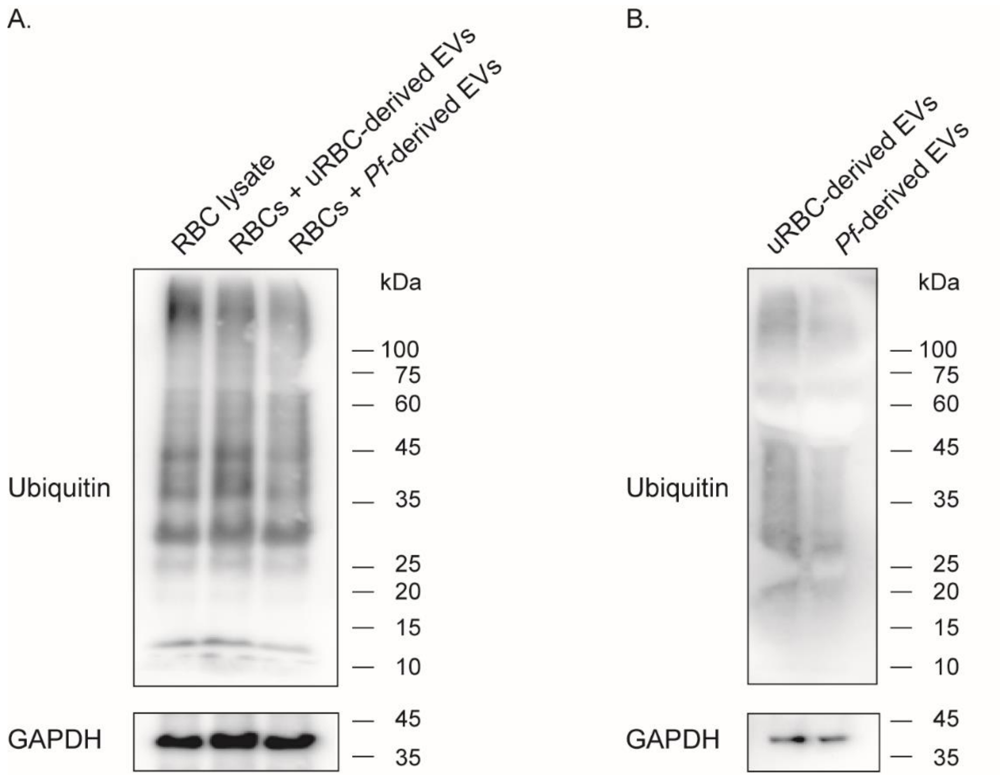

Supplementary Figure 10: Global ubiquitination levels remain unaffected following *Pf*-derived EVs treatment.
(A) Naïve RBCs were incubated with *Pf*-derived EVs and control EVs and analyzed for total ubiquitination by Western blot analysis using an anti-ubiquitin antibody. (B) EVs were analyzed for total ubiquitination by immunodetection using an anti-ubiquitin antibody. Representative results of four independent repeats. Source data are provided in the source data file.

Supplementary Figure 11

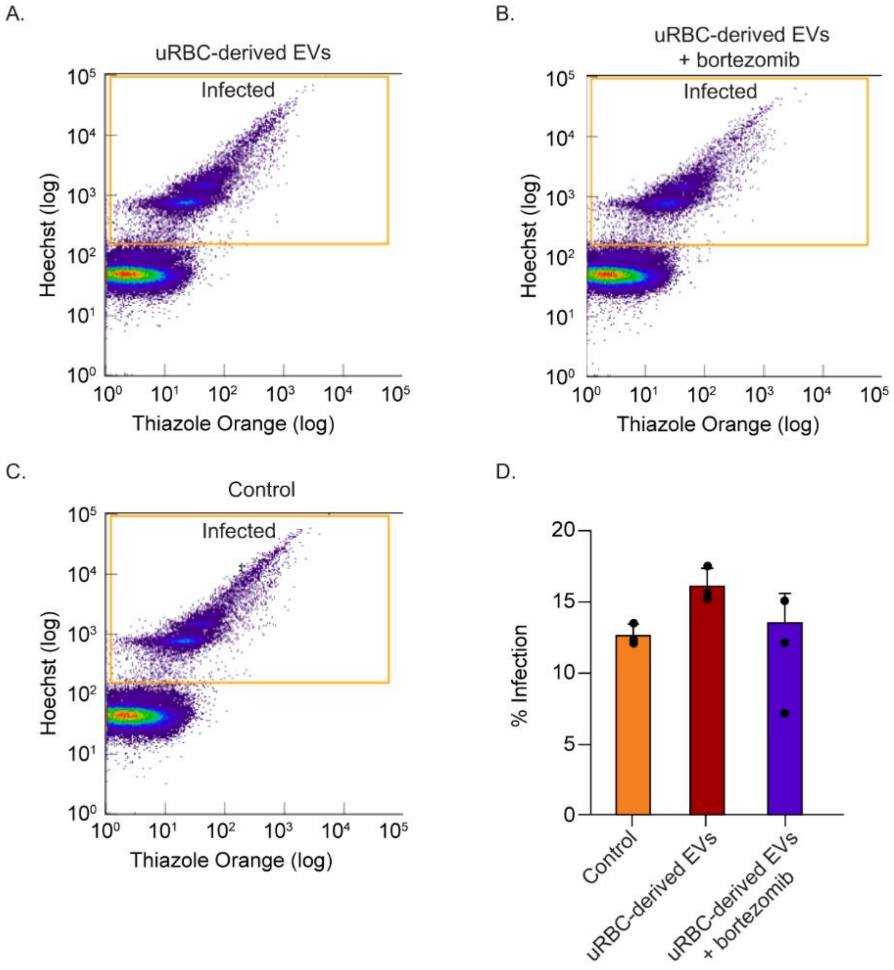

Supplementary Figure 11: The presence of sub-lethal concentration of bortezomib in EV treated culture does not affect parasitemia.
uRBC-derived EVs, either untreated (A) or pretreated with bortezomib (B), were incubated with naïve RBCs. Magnet-purified *Pf* were then introduced to the cells and parasitemia levels were monitored by flow cytometry. Treatment of RBCs with medium was used as control (C). (D) Average parasitemia levels from three experiments. Error bars represent SD.

Supplementary Figure 12

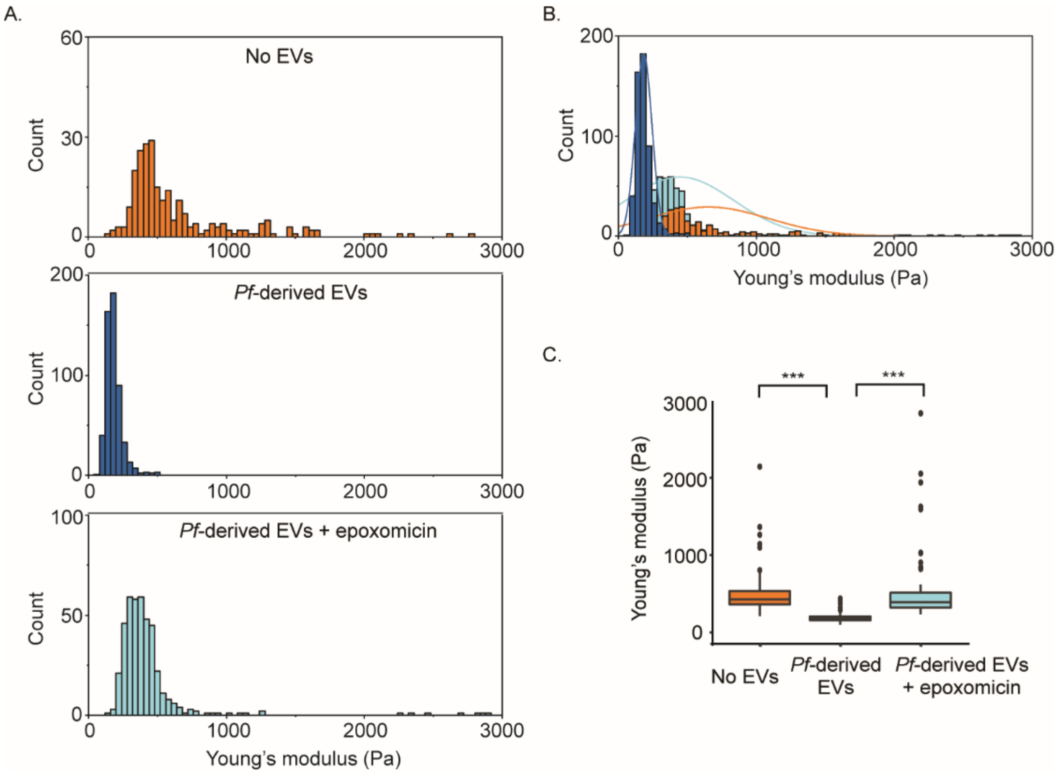

Supplementary Figure 12: Mechanical changes in RBCs measured by AFM following incubation with *Pf*-derived EV pretreated with epoxomicin, showing reversal of the mechanical changes induced by *Pf*-derived EVs.

Mechanical changes measured by AFM following *Pf*-derived EV incubation with naïve RBCs. *Pf*-derived EVs or *Pf*-derived EVs pretreated with an irreversible proteasome inhibitor (epoxomicin) were incubated with naïve RBCs. As control, untreated RBCs were used.

Pretreated cells were deposited on a mica surface for AFM topography and mechanical measurements. (A) Distributions of Young's modulus values, each count in the histogram represents data from a separate indentation, 9 different positions were measured near the center of each cell. (B) Superposition of the Young's modulus distributions of the different treatments. (C) Box plot of the average value from two different experiments. Number of cells measured were: for no EVs n=65, *Pf*-derived EVs n=85, *Pf*-derived Evs+epoxomicin n=72, where more than 60 cells were measured in each sample. Boxes represent the 25-75 percentiles of the sample distribution, with black vertical lines representing the 1.5xIQR (interquartile range). The black dots represent outliers. Black horizontal line represents the median. Significance was calculated by comparing the two treatments with a 2-way ANOVA (accounting for treatment and batch), followed by Tukey's post-hoc test (***p<0.001).

Supplementary Figure 13

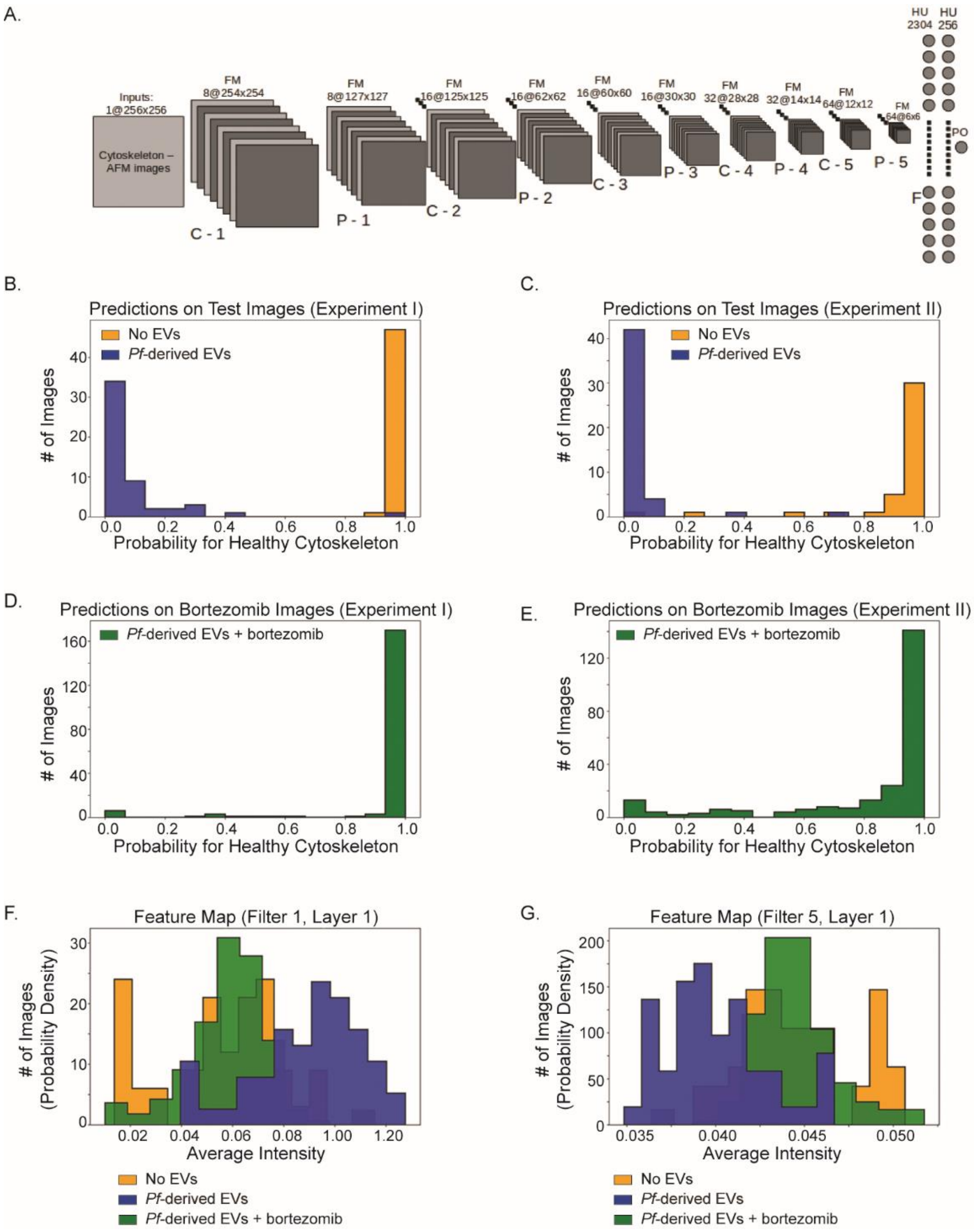

Supplementary Figure 13: Machine learning image processing.
(A) CNN model architecture used in this study, composed of 5 CNN + ReLU (rectified linear unit activation) + Max-pooling layers (where 2x2 cells are replaced by a 1 x1 cell with value of the maximum pixel, with strides of 2x2), flattened and connected to the fully connected layer + ReLU + the output unit with sigmoid activation. C: convolution (3x3 kernel) + ReLU; P: Max-pooling, strides 2x2; FM: feature maps; F: flatten; HU: hidden units; PO: probability output. (B) Predictions of the probability for a healthy cytoskeleton made by the model on testing set from data set 1. The x axis is the probability and the y axis is the number of images (100 images in total). Control (yellow bins) are the healthy RBC (no EVs) cytoskeleton images and Exo (blue bins) are the cytoskeleton images of *Pf*-derived EV-exposed RBC. (C) Predictions of the probability for a healthy cytoskeleton made by the model on testing set from data set 2. The x-axis is the probability and the y-axis the number of images (88 images in total). (D) Predictions of the probability for a healthy cytoskeleton made by the model on bortezomib-treated cytoskeleton images (green bins) for data set 1. The x axis and the y axis as above (188 images in total). (E) Predictions of the probability for a healthy cytoskeleton made by the model on bortezomib-treated cytoskeleton images (green bins) for data set 2. x-axis and y-axis as above (236 images in total). (F) Intensity distribution (averaged feature maps) for all images in testing set and treated *Pf*-derived EV exposed RBC cytoskeleton images for feature map 1 in layer 1 of the CNN. This filter activates the backbone regions. (G) As for (F), but for feature map 5 in layer 1 which activated the holes.

Supplementary Figure 14

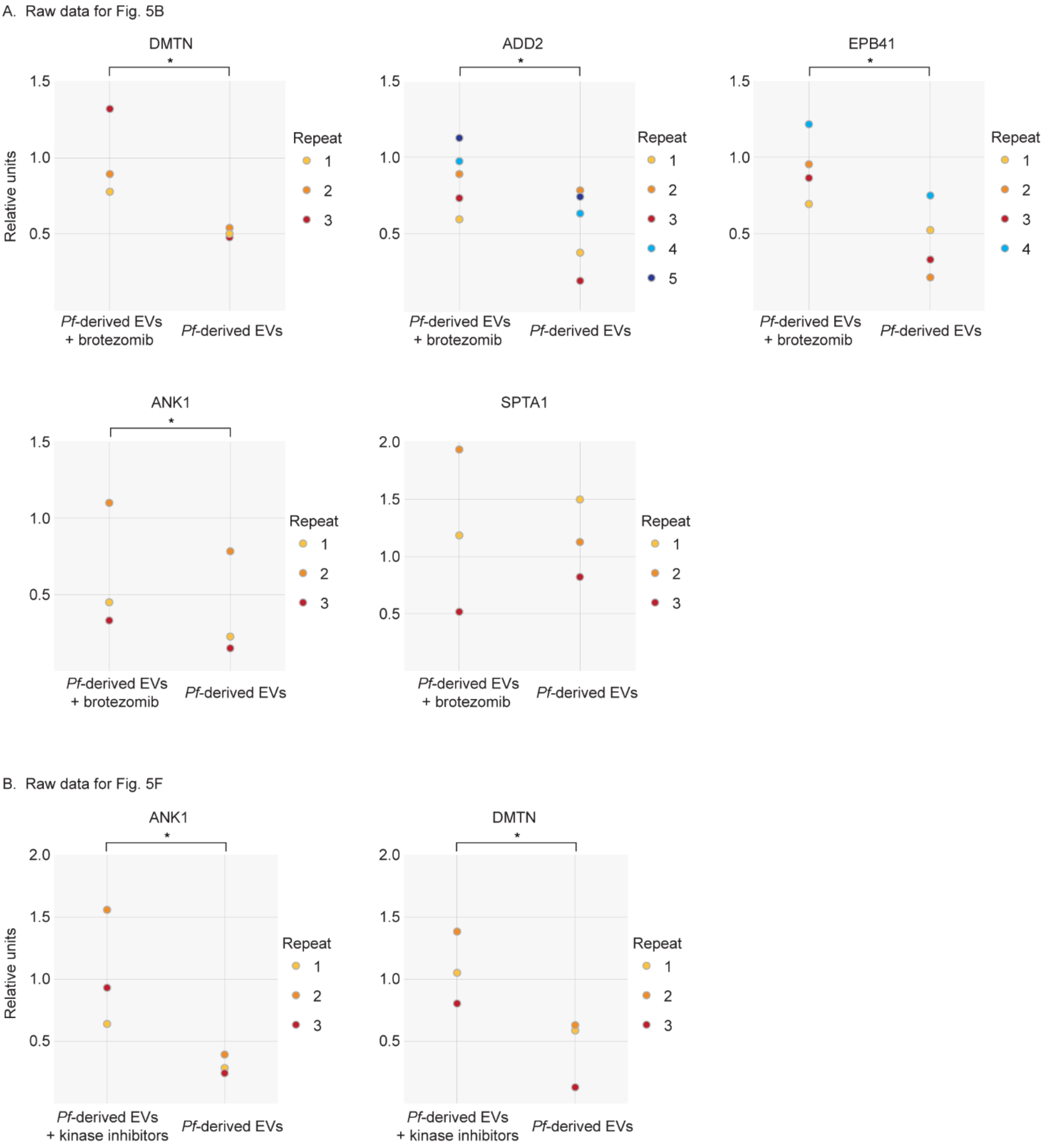

Supplementary Figure 14: Raw data used to generate figures 5B and 5F.
(A) Raw data (relative units) from three different experiments that were used to generate Figure 5B. Differentially colored dots represent biological repeats. Statistical analysis was performed using paired two way t-test, ((DMTN *p=0.0102, ADD2 *p = 0.01339, EPB41 *p = 0.02769, ANK *p = 0.01339*). (B) Raw data (relative units) from three different experiments that were used to generate Figure 5F. Differentially colored dots represent biological repeats. Statistical analysis was performed using paired two way t-test, (ANK *p = 0.0421, DMTN *p = 0.0402). Source data are provided in the source data file.

Supplementary Figure 15

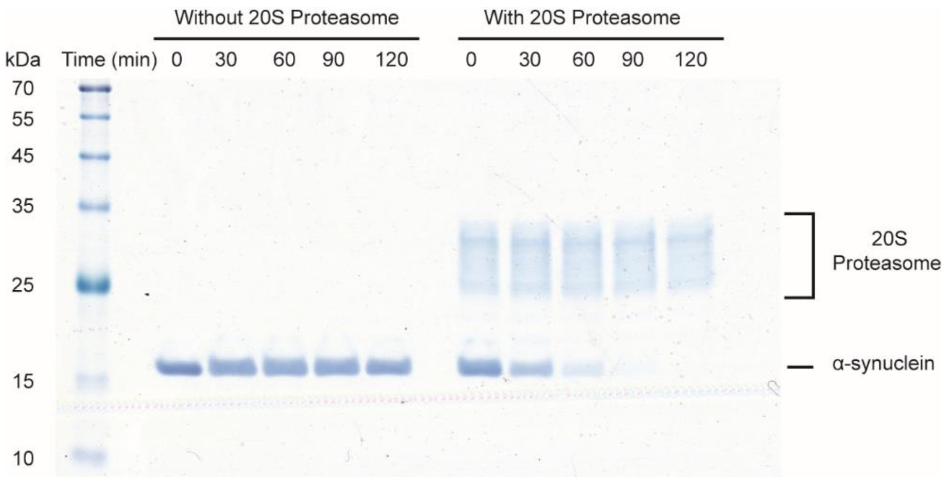

Supplementary Figure 15: Validation of 20S proteasome activity.
In-vitro degradation assays using the model substrate α-synuclein. The time-dependent assay indicates that α-synuclein is stable in the absence of the 20S proteasome. However, following the addition of highly purified 20S proteasome particles, α -synuclein is rapidly degraded.

Supplementary Figure 16

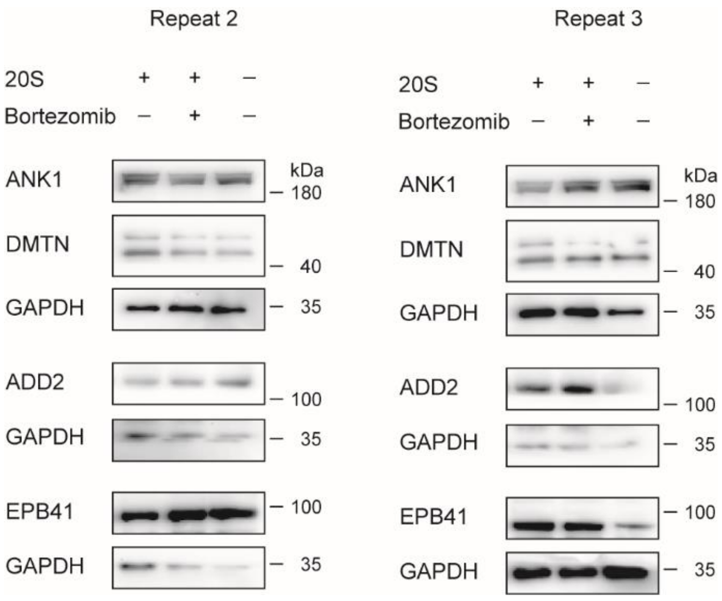

Supplementary Figure 16: RBC cytoskeleton proteins are not degraded by purified 20S proteasomes.
Two additional repeats of the degradation assays shown in Fig. 5D. Source data are provided in the source data file.

Supplementary Figure 17

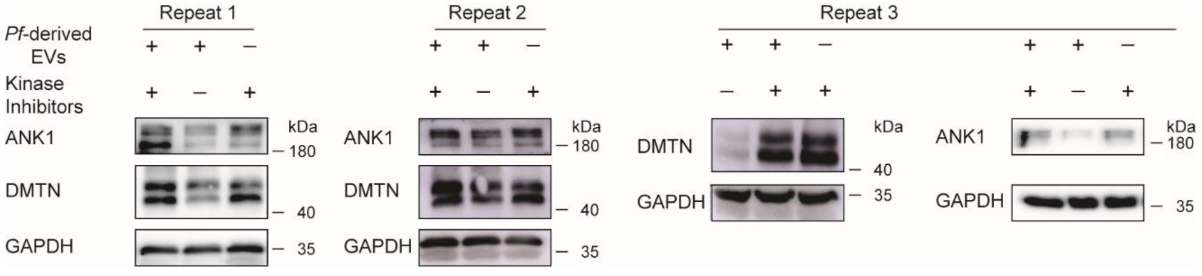

Supplementary Figure 17: The RBC cytoskeletal proteins ankiryn and dematin are stabilized in response to treatment with *Pf*-derived EVs in the presence of kinase inhibitors.
Two additional repeats of the assays shown in Fig. 5E. Source data are provided in the source data file.

Supplementary Figure 18

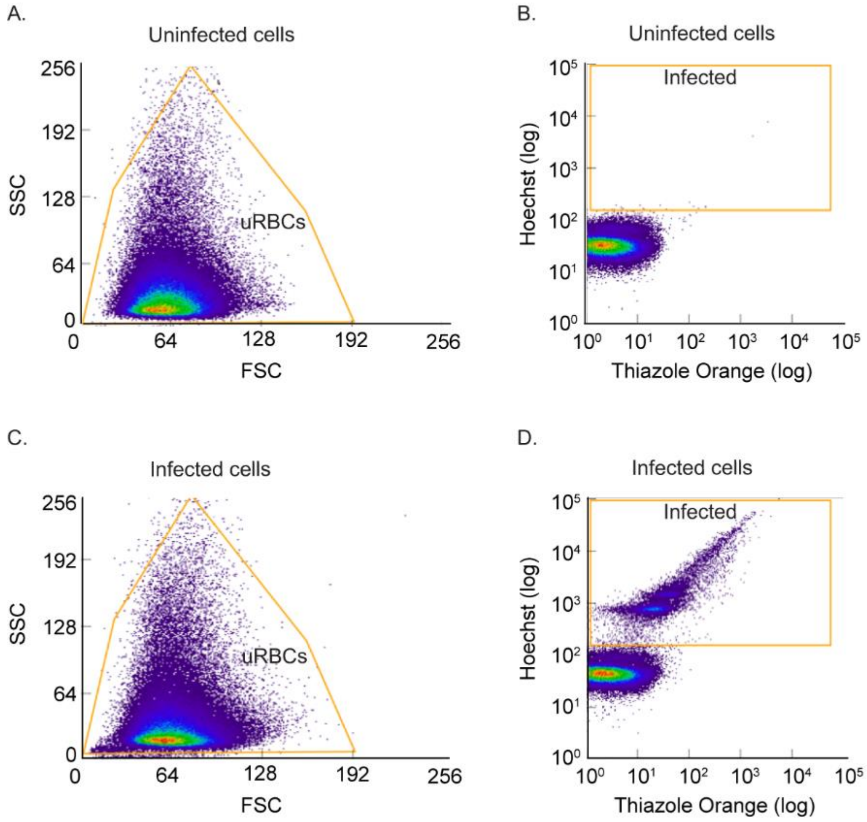

Supplementary Figure 18: Gating strategy for FACS analysis in Figure S11
Total populations of uninfected (A) and infected RBCs (C) were plotted using scatter plot. Gating was done in order to remove cell debris and form the total RBC population. In the next step, another scatter plot was generated, using the Thiazole Orange intensity in the X axis and Hoechst intensity at the Y axis. The "infected" gates (B, D) refer to the *Pf*-iRBC cell populations.

TABLE S1A: Proteomics Analysis of Plasmodium EV Fractions - Human

					Combined Analysis				Biological Replicate 1				Biological Replicate 2			
Accession	Genename	Description	CalcMr	Species	Score[a]	Cov(%)[b]	SigM[c]	SpC[d]	Score[a]	Cov(%)[b]	SigM[c]	SpC[d]	Score[a]	Cov(%)[b]	SigM[c]	SpC[d]
P04040	CAT	CAT Catalase Tax_Id=9606	59756	Homo sapiens	9605.21	75	48	190	9451.39	75	47	144	2375.32	46	21	44
P02768	ALB	ALB Serum albumin Tax_Id=9606	69366	Homo sapiens	7852.52	85	53	210	7769.89	85	52	169	1627.53	34	16	39
P13645	KRT10	KRT10 Keratin, type I cytoskeletal 10 Tax_Id=9606	58827	Homo sapiens	7635.41	56	37	316	7409.03	56	36	165	4381.42	52	30	146
P04264	KRT1	KRT1 Keratin, type II cytoskeletal 1 Tax_Id=9606	66038	Homo sapiens	7533.04	58	42	411	6763.56	53	40	208	5703.79	54	36	199
P02549	SPTA1	SPTA1 Spectrin alpha chain, erythrocytic 1 Tax_Id=9606	280014	Homo sapiens	6157.06	32	47	76	5740	30	44	46	2607.18	18	24	29
P35908	KRT2	KRT2 Keratin, type II cytoskeletal 2 epidermal Tax_Id=9606	65432	Homo sapiens	5706.85	65	42	193	5037.92	62	38	94	3866.46	49	32	98
P68871	HBB	HBB Hemoglobin subunit beta Tax_Id=9606	15998	Homo sapiens	5548.13	97	20	1122	5558.56	97	20	715	3955.82	97	16	403
P32119	PRDX2	PRDX2 Peroxiredoxin-2 Tax_Id=9606	21891	Homo sapiens	5177.2	89	26	388	5004.54	89	25	225	4065.38	87	23	163
P11277	SPTB	SPTB Spectrin beta chain, erythrocytic Tax_Id=9606	246468	Homo sapiens	5071.56	31	43	49	4925.67	29	42	44	400.13	8	5	5
P35527	KRT9	KRT9 Keratin, type I cytoskeletal 9 Tax_Id=9606	62064	Homo sapiens	4808.46	56	29	250	4662.36	56	29	123	3067.49	45	21	119
P00352	ALDH1A1	ALDH1A1 Retinal dehydrogenase 1 Tax_Id=9606	54861	Homo sapiens	4806.55	65	30	61	4562.3	65	30	40	1727.83	45	15	20
P00915	CA1	CA1 Carbonic anhydrase 1 Tax_Id=9606	28870	Homo sapiens	4425.52	79	20	268	4423.89	79	20	187	2403.99	60	15	77
P13798	APEH	APEH Acylamino-acid-releasing enzyme Tax_Id=9606	81224	Homo sapiens	4263.74	43	25	62	4091.87	42	24	40	1387.83	25	11	21
Q13228	SELENBP1	SELENBP1 Selenium-binding protein 1 Tax_Id=9606	52391	Homo sapiens	4074.14	58	27	121	4058.9	58	27	79	2494.38	47	18	42
P30041	PRDX6	PRDX6 Peroxiredoxin-6 Tax_Id=9606	25035	Homo sapiens	3912.15	78	23	182	3846.46	78	23	104	3089.38	78	23	78
P69905	HBA1HBA2	HBA1 Hemoglobin subunit alpha Tax_Id=9606	15257	Homo sapiens	3887.46	76	14	843	3899.62	76	14	497	2707.14	76	10	342
P04406	GAPDH	GAPDH Glyceraldehyde-3-phosphate dehydrogenase Tax_Id=9606	36053	Homo sapiens	3853.42	74	23	105	3611.03	74	22	58	2045.2	59	13	46
P02042	HBD	HBD Hemoglobin subunit delta Tax_Id=9606	16055	Homo sapiens	3730.81	95	15	743	3704.19	95	15	459	2948.79	95	14	282
P30043	BLVRB	BLVRB Flavin reductase (NADPH) Tax_Id=9606	22119	Homo sapiens	3718.8	95	16	176	3718.65	95	16	117	2282.3	87	12	59
P50395	GDI2	GDI2 Rab GDP dissociation inhibitor beta Tax_Id=9606	50663	Homo sapiens	3656.46	73	27	61	3376.45	70	25	45	1590.72	49	16	16
P08779	KRT16	KRT16 Keratin, type I cytoskeletal 16 Tax_Id=9606	51267	Homo sapiens	3459.8	51	21	100	3019.15	48	19	58	1456.12	28	11	37
P07195	LDHB	LDHB L-lactate dehydrogenase B chain Tax_Id=9606	36638	Homo sapiens	3422.16	69	20	77	2009.94	43	14	24	2855.49	69	20	52
P02533	KRT14	KRT14 Keratin, type I cytoskeletal 14 Tax_Id=9606	51561	Homo sapiens	3327.47	49	20	114	3001.04	45	18	58	1687.36	41	14	50
P23526	AHCY	AHCY Adenosylhomocysteinase Tax_Id=9606	47716	Homo sapiens	3300.4	41	22	54	3196.52	41	21	31	1554.02	35	13	21
P06744	GPI	GPI Glucose-6-phosphate isomerase Tax_Id=9606	63147	Homo sapiens	3299.89	52	23	35	3007.87	48	22	26	1015.3	27	7	8
P02538	KRT6A	KRT6A Keratin, type II cytoskeletal 6A Tax_Id=9606	60045	Homo sapiens	3196.54	42	24	111	2813.66	37	20	49	1860.45	34	17	61
P48668	KRT6C	KRT6C Keratin, type II cytoskeletal 6C Tax_Id=9606	60025	Homo sapiens	3174.6	44	24	117	2921.81	39	21	53	1860.45	36	17	62
P13716	ALAD	ALAD Delta-aminolevulinic acid dehydratase Tax_Id=9606	36294	Homo sapiens	3119.74	58	20	49	3040.6	58	20	31	1095.08	28	9	16
P04075	ALDOA	ALDOA Fructose-bisphosphate aldolase A Tax_Id=9606	39420	Homo sapiens	3066.83	59	18	54	2929.17	59	18	27	1550.47	50	13	26
P13647	KRT5	KRT5 Keratin, type II cytoskeletal 5 Tax_Id=9606	62378	Homo sapiens	2994	40	25	115	2864.67	37	23	54	1806.49	32	16	59
P00918	CA2	CA2 Carbonic anhydrase 2 Tax_Id=9606	29246	Homo sapiens	2856.59	47	15	118	2811.17	47	15	80	1062.78	37	7	37
Q06830	PRDX1	PRDX1 Peroxiredoxin-1 Tax_Id=9606	22110	Homo sapiens	2844.99	50	16	128	2640.29	50	15	78	2134.95	50	16	48
Q99497	PARK7	PARK7 Protein DJ-1 Tax_Id=9606	19891	Homo sapiens	2726.98	85	15	87	2572.52	85	14	59	1427.89	57	10	28
P11142	HSPA8	HSPA8 Heat shock cognate 71 kDa protein Tax_Id=9606	70898	Homo sapiens	2682.68	31	18	70	2547.62	31	18	42	1502.15	29	13	29
P06733	ENO1	ENO1 Alpha-enolase Tax_Id=9606	47169	Homo sapiens	2479.13	50	17	52	2222.8	41	15	30	1495.16	33	11	21
Q86VP6	CAND1	CAND1 Cullin-associated NEDD8-dissociated protein 1 Tax_Id=9606	136376	Homo sapiens	2457.75	24	22	31	2228.54	22	19	19	1070.61	15	12	12
P60174	TPI1	TPI1 Triosephosphate isomerase Tax_Id=9606	30791	Homo sapiens	2305.34	68	14	82	1940.73	61	12	46	2283.95	68	14	36
P22392-2	NME2	NME2 Isoform 3 of Nucleoside diphosphate kinase B Tax_Id=9606	30137	Homo sapiens	2276.71	54	17	82	1726.91	49	13	30	1615.7	49	13	50
P00568	AK1	AK1 Adenylate kinase isoenzyme 1 Tax_Id=9606	21634	Homo sapiens	2135.17	68	14	64	2084.09	68	14	42	1173.38	53	11	21
P52209	PGD	PGD 6-phosphogluconate dehydrogenase, decarboxylating Tax_Id=9606	53140	Homo sapiens	2120.01	41	15	31	2023.04	39	15	22	900.76	23	7	9
Q6XQN6	NAPRT1	NAPRT1 Nicotinate phosphoribosyltransferase Tax_Id=9606	57578	Homo sapiens	2116.6	40	15	24	2096.4	40	14	17	568.69	17	7	7
P48506	GCLC	GCLC Glutamate--cysteine ligase catalytic subunit Tax_Id=9606	72765	Homo sapiens	2110.7	22	15	35	1373.9	14	10	13	1291.73	22	12	20
O15067	PFAS	PFAS Phosphoribosylformylglycinamidine synthase Tax_Id=9606	144734	Homo sapiens	2084.18	19	17	23	2079.79	19	17	17	433.71	6	4	5
P07738	BPGM	BPGM Bisphosphoglycerate mutase Tax_Id=9606	30005	Homo sapiens	2021.27	60	12	53	1804.63	60	11	31	1563.9	42	10	22
P00558	PGK1	PGK1 Phosphoglycerate kinase 1 Tax_Id=9606	44614	Homo sapiens	1978.06	53	13	63	1976.11	53	13	40	1252.83	37	9	23
P30566	ADSL	ADSL Adenylosuccinate lyase Tax_Id=9606	54889	Homo sapiens	1854.54	46	14	19	1804.45	46	14	15	437.28	8	4	4
P00390	GSR	GSR Glutathione reductase, mitochondrial Tax_Id=9606	56257	Homo sapiens	1759.28	37	12	18	1635.06	37	12	13	616.73	18	5	5
P50990	CCT8	CCT8 T-complex protein 1 subunit theta Tax_Id=9606	59620	Homo sapiens	1739.67	34	14	20	1601.76	34	13	14	547.27	12	5	6
P49247	RPIA	RPIA Ribose-5-phosphate isomerase Tax_Id=9606	33269	Homo sapiens	1676.31	39	9	35	1232.46	39	9	14	1348.72	37	8	21

P29401	TKT	TKT Transketolase Tax_Id=9606	67877	Homo sapiens	1637.73	30	14	30	1594.57	27	13	15	872.56	29	8	14
P31939	ATIC	ATIC Bifunctional purine biosynthesis protein PURH Tax_Id=9606	64615	Homo sapiens	1633.18	40	11	21	1635.06	39	11	13	581.9	20	5	8
P62937	PPIA	PPIA Peptidyl-prolyl cis-trans isomerase A Tax_Id=9606	18012	Homo sapiens	1622.79	82	11	52	1472.02	82	11	32	948.43	44	8	20
P29144	TPP2	TPP2 Tripeptidyl-peptidase 2 Tax_Id=9606	138350	Homo sapiens	1588.79	17	13	14	1488.71	16	12	12	104.28	3	2	2
P01857	IGHG1	IGHG1 Ig gamma-1 chain C region Tax_Id=9606	36105	Homo sapiens	1539.22	48	11	17	1548.11	46	11	16	154.26	6	1	1
P40925	MDH1	MDH1 Malate dehydrogenase, cytoplasmic Tax_Id=9606	36426	Homo sapiens	1504.83	36	10	35	1092.38	30	7	13	1283.5	33	10	22
P48637	GSS	GSS Glutathione synthetase Tax_Id=9606	52384	Homo sapiens	1478.68	38	10	18	1423.53	37	10	13	547.64	10	4	5
P50991	CCT4	CCT4 T-complex protein 1 subunit delta Tax_Id=9606	57924	Homo sapiens	1453.61	19	9	12	1458.51	19	9	11	133.07	6	1	1
P54578	USP14	USP14 Ubiquitin carboxyl-terminal hydrolase 14 Tax_Id=9606	56069	Homo sapiens	1434.17	28	9	23	1340.79	26	9	16	531.59	15	6	7
P07384	CAPN1	CAPN1 Calpain-1 catalytic subunit Tax_Id=9606	81890	Homo sapiens	1385.2	22	11	17	1259.56	21	10	12	391.2	11	4	5
P60709	ACTB	ACTB Actin, cytoplasmic 1 Tax_Id=9606	41736	Homo sapiens	1369.17	43	12	35	1292.33	43	12	19	944.15	36	9	16
P69891	HBG1	HBG1 Hemoglobin subunit gamma-1 Tax_Id=9606	16140	Homo sapiens	1364.32	75	8	112	962.12	58	5	61	1139.32	67	7	51
P22314	UBA1	UBA1 Ubiquitin-like modifier-activating enzyme 1 Tax_Id=9606	117849	Homo sapiens	1360.14	15	10	22	1220.69	13	9	10	937.97	9	7	12
P78417	GSTO1	GSTO1 Glutathione S-transferase omega-1 Tax_Id=9606	27565	Homo sapiens	1333.8	38	10	43	1093	35	9	22	831.61	37	9	21
P78371	CCT2	CCT2 T-complex protein 1 subunit beta Tax_Id=9606	57488	Homo sapiens	1331.55	27	11	14	1192.92	27	10	11	267.84	7	3	3
P09960	LTA4H	LTA4H Leukotriene A-4 hydrolase Tax_Id=9606	69285	Homo sapiens	1324.45	23	10	26	1333.1	23	10	15	814.13	16	6	11
P37837	TALDO1	TALDO1 Transaldolase Tax_Id=9606	37540	Homo sapiens	1296.43	40	11	42	1256.57	34	10	25	688.77	34	7	16
P17174	GOT1	GOT1 Aspartate aminotransferase, cytoplasmic Tax_Id=9606	46247	Homo sapiens	1283.31	48	12	29	1012.62	33	8	14	858.82	37	10	15
P30086	PEBP1	PEBP1 Phosphatidylethanolamine-binding protein 1 Tax_Id=9606	21056	Homo sapiens	1229.89	76	9	32	1096.71	73	8	22	502.89	36	5	9
P49368	CCT3	CCT3 T-complex protein 1 subunit gamma Tax_Id=9606	60534	Homo sapiens	1227.38	32	11	13	1220.76	32	11	11	267.67	5	2	2
P01860	IGHG3	IGHG3 Ig gamma-3 chain C region Tax_Id=9606	41287	Homo sapiens	1177.27	28	9	12	1174.38	26	9	11	154.26	6	1	1
Q9NY33	DPP3	DPP3 Dipeptidyl peptidase 3 Tax_Id=9606	82589	Homo sapiens	1160.64	21	9	21	1156.66	19	9	13	581.44	12	5	8
P01024		C3 Complement C3 Tax_Id=9606	187148	Homo sapiens	1158.53	11	9	9	1150.62	11	9	9				
P00492	HPRT1	HPRT1 Hypoxanthine-guanine phosphoribosyltransferase Tax_Id=9606	24579	Homo sapiens	1135.87	44	7	29	1135.14	44	7	17	816.78	35	6	12
P21980	TGM2	TGM2 Protein-glutamine gamma-glutamyltransferase 2 Tax_Id=9606	77329	Homo sapiens	1110.55	20	8	19	1092.57	20	8	10	696.13	12	5	9
P01834	IGKC	IGKC Ig kappa chain C region Tax_Id=9606	11608	Homo sapiens	1107.91	85	8	12	1112.36	85	8	11	154.26	16	1	1
P23528	CFL1	CFL1 Cofilin-1 Tax_Id=9606	18502	Homo sapiens	1095.46	46	5	27	1096.71	46	5	20	417.59	27	3	7
Q14525	KRT33B	KRT33B Keratin, type I cuticular Ha3-II Tax_Id=9606	46213	Homo sapiens	1092.68	27	9	15	154.59	2	1	4	804.46	27	9	11
Q15323	KRT31	KRT31 Keratin, type I cuticular Ha1 Tax_Id=9606	47237	Homo sapiens	1092.68	26	9	15	154.59	2	1	4	768.52	26	9	11
P02787		TF Serotransferrin Tax_Id=9606	77064	Homo sapiens	1088.46	15	10	15	1099.92	15	10	15				
Q16775	HAGH	HAGH Hydroxyacylglutathione hydrolase, mitochondrial Tax_Id=9606	33805	Homo sapiens	1066.4	27	9	17	1070.25	27	9	15	139.49	5	1	2
P62987	UBA52	UBA52 Ubiquitin-60S ribosomal protein L40 Tax_Id=9606	14728	Homo sapiens	1049.92	43	7	40	1052.79	43	7	28	409.13	41	4	12
Q99832	CCT7	CCT7 T-complex protein 1 subunit eta Tax_Id=9606	59366	Homo sapiens	1036.91	28	11	13	1020.92	28	11	11	58.92	3	2	2
Q15631	TSN	TSN Translin Tax_Id=9606	26182	Homo sapiens	1002	29	5	9	990.42	25	5	8	62.18	9	1	1
P63104	YWHAZ	YWHAZ 14-3-3 protein zeta/delta Tax_Id=9606	27745	Homo sapiens	986.52	36	7	21	969.25	36	7	16	457.64	24	3	5
P07900	HSP90AA1	HSP90AA1 Heat shock protein HSP 90-alpha Tax_Id=9606	84659	Homo sapiens	984.03	15	8	9	975.64	15	8	8	35.96	6	1	1
P30613	PKLR	PKLR Pyruvate kinase PKLR Tax_Id=9606	61830	Homo sapiens	969.62	26	9	9	835.28	26	8	8	154.26	2	1	1
P01859	IGHG2	IGHG2 Ig gamma-2 chain C region Tax_Id=9606	35900	Homo sapiens	966.77	31	8	10	968.13	29	8	10				
P24666	ACP1	ACP1 Low molecular weight phosphotyrosine protein phosphatase Tax_Id=9606	18042	Homo sapiens	961.02	30	6	24	962.12	30	6	15	422.78	20	3	9
P62826	RAN	RAN GTP-binding nuclear protein Ran Tax_Id=9606	24423	Homo sapiens	961.02	28	7	18	962.12	28	7	12	418.89	28	4	6
Q9GZP4	PITHD1	PITHD1 PITH domain-containing protein 1 Tax_Id=9606	24177	Homo sapiens	959.81	53	8	21	944.06	53	8	16	348.3	29	4	5
O75083	WDR1	WDR1 WD repeat-containing protein 1 Tax_Id=9606	66193	Homo sapiens	957.65	17	7	9	827.53	17	6	7	172.1	10	2	2
P40227	CCT6A	CCT6A T-complex protein 1 subunit zeta Tax_Id=9606	58024	Homo sapiens	956.81	21	8	10	950.67	21	8	8	129.82	7	2	2
P00491	PNP	PNP Purine nucleoside phosphorylase Tax_Id=9606	32117	Homo sapiens	913.41	31	6	24	669.04	17	4	9	808.87	31	6	15
P00338	LDHA	LDHA L-lactate dehydrogenase A chain Tax_Id=9606	36688	Homo sapiens	908.97	30	6	22	776.42	22	6	10	708.34	27	5	12
Q16531	DDB1	DDB1 DNA damage-binding protein 1 Tax_Id=9606	126968	Homo sapiens	898.09	9	7	13	893.26	9	7	7	433.1	7	5	6
P08107	HSPA1AHSPA	HSPA1A Heat shock 70 kDa protein 1A/1B Tax_Id=9606	70052	Homo sapiens	890.4	13	6	26	759.54	13	6	12	617.38	10	4	14
P26447	S100A4	S100A4 Protein S100-A4 Tax_Id=9606	11728	Homo sapiens	888.6	35	5	17	888.68	35	5	15	64.86	11	1	2
P02647	APOA1	APOA1 Apolipoprotein A-I Tax_Id=9606	30777	Homo sapiens	888.23	36	8	21	878.04	36	8	22				
P16157	ANK1	ANK1 Ankyrin-1 Tax_Id=9606	206265	Homo sapiens	886.55	8	7	7	879.68	8	7	7				
O43790	KRT86	KRT86 Keratin, type II cuticular Hb6 Tax_Id=9606	53500	Homo sapiens	864.7	20	7	9					653.85	20	7	9
P01876	IGHA1	IGHA1 Ig alpha-1 chain C region Tax_Id=9606	37654	Homo sapiens	862.95	18	6	7	868.99	18	6	7				

P17987	TCP1	TCP1 T-complex protein 1 subunit alpha Tax_Id=9606	60343	Homo sapiens	839.8	16	7	8	569.87	11	5	5	345.97	6	3	3
O76011	KRT34	KRT34 Keratin, type I cuticular Ha4 Tax_Id=9606	49423	Homo sapiens	837.06	17	7	7					634.31	17	7	7
Q13630	TSTA3	TSTA3 GDP-L-fucose synthase Tax_Id=9606	35892	Homo sapiens	823.17	20	6	16	683.82	20	5	8	532.47	15	5	8
P50502	ST13	ST13 Hsc70-interacting protein Tax_Id=9606	41331	Homo sapiens	820.82	15	6	20	814.23	14	6	15	398.16	15	4	5
Q04760	GLO1	GLO1 Lactoylglutathione lyase Tax_Id=9606	20777	Homo sapiens	809.86	32	7	23	796.59	32	7	17	211.09	13	3	6
P52565	ARHGDIA	ARHGDIA Rho GDP-dissociation inhibitor 1 Tax_Id=9606	23207	Homo sapiens	800.55	31	5	18	811.1	31	6	12	422.78	22	3	7
P02675	FGB	FGB Fibrinogen beta chain Tax_Id=9606	55928	Homo sapiens	796.44	20	7	7	788.2	20	7	7				
P09104	ENO2	ENO2 Gamma-enolase Tax_Id=9606	47268	Homo sapiens	779.24	16	5	19	781.36	16	5	10	557.04	12	3	9
P22061	PCMT1	PCMT1 Protein-L-isoaspartate(D-aspartate) O-methyltransferase Tax_Id=9606	24636	Homo sapiens	777.33	46	6	26	744.02	46	6	15	605.59	33	5	11
Q86Y46	KRT73	KRT73 Keratin, type II cytoskeletal 73 Tax_Id=9606	58923	Homo sapiens	755.16	18	6	29	593.67	13	4	14	454.85	13	4	15
P34932	HSPA4	HSPA4 Heat shock 70 kDa protein 4 Tax_Id=9606	94331	Homo sapiens	753.08	9	6	9	558.35	9	4	5	306.76	4	3	4
P30046	DDT	DDT D-dopachrome decarboxylase Tax_Id=9606	12711	Homo sapiens	737.42	46	5	15	714.18	46	5	8	555.81	46	5	7
P48643	CCT5	CCT5 T-complex protein 1 subunit epsilon Tax_Id=9606	59671	Homo sapiens	711.67	14	6	9	671.33	13	5	6	192.39	8	3	3
P07451	CA3	CA3 Carbonic anhydrase 3 Tax_Id=9606	29557	Homo sapiens	694.07	27	5	8	585.45	23	5	7	90.56	7	1	1
Q00796	SORD	SORD Sorbitol dehydrogenase Tax_Id=9606	38324	Homo sapiens	692.16	23	5	6	692.94	23	5	5	154.26	7	1	1
Q3LXA3	DAK	DAK Bifunctional ATP-dependent dihydroxyacetone kinase/FAD-AMP lyase (cyclizing) Tax	58947	Homo sapiens	678.18	17	5	10	620.82	13	5	6	74.62	8	1	5
P06132	UROD	UROD Uroporphyrinogen decarboxylase Tax_Id=9606	40787	Homo sapiens	674.34	20	5	13	674.37	20	5	6	447.55	20	5	7
P09211	GSTP1	GSTP1 Glutathione S-transferase P Tax_Id=9606	23355	Homo sapiens	667.92	36	5	19	609.91	36	5	12	475.65	31	4	7
P13489	RNH1	RNH1 Ribonuclease inhibitor Tax_Id=9606	49973	Homo sapiens	667.89	20	6	14	671.04	20	6	11	410.36	10	3	3
Q9UKV8	AGO2	AGO2 Protein argonaute-2 Tax_Id=9606	97208	Homo sapiens	649.88	13	6	7	637.74	13	6	6	33.21	3	1	1
P35611	ADD1	ADD1 Alpha-adducin Tax_Id=9606	80955	Homo sapiens	645.42	10	5	6	598.61	10	5	5	61.45	1	1	1
P11413	G6PD	G6PD Glucose-6-phosphate 1-dehydrogenase Tax_Id=9606	59256	Homo sapiens	635.45	18	5	6	629.36	15	5	5	82.72	7	1	1
O95336	PGLS	PGLS 6-phosphogluconolactonase Tax_Id=9606	27546	Homo sapiens	625.01	32	6	6	614.2	32	6	6				
P61970	NUTF2	NUTF2 Nuclear transport factor 2 Tax_Id=9606	14478	Homo sapiens	614.97	40	5	17	615.3	40	5	7	319.29	28	4	8
P07954		FH Fumarate hydratase, mitochondrial Tax_Id=9606	54637	Homo sapiens	592.95	19	5	8	593.19	17	5	5	356.42	10	3	3
P21281	ATP6V1B2	ATP6V1B2 V-type proton ATPase subunit B, brain isoform Tax_Id=9606	56500	Homo sapiens	590.02	18	5	5	589.16	18	5	5				
P52566	ARHGDIB	ARHGDIB Rho GDP-dissociation inhibitor 2 Tax_Id=9606	22988	Homo sapiens	588.56	34	5	12	593.18	34	5	9	110.69	10	2	3
P18669	PGAM1	PGAM1 Phosphoglycerate mutase 1 Tax_Id=9606	28803	Homo sapiens	586.12	25	4	7	405.63	25	4	5	228.48	15	2	3
P55786	NPEPPS	NPEPPS Puromycin-sensitive aminopeptidase Tax_Id=9606	103276	Homo sapiens	578.79	11	5	5	423.77	8	3	3	95.3	4	2	2
P06703	S100A6	S100A6 Protein S100-A6 Tax_Id=9606	10179	Homo sapiens	572.35	30	5	11	571.24	30	5	11				
P14923	JUP	JUP Junction plakoglobin Tax_Id=9606	81745	Homo sapiens	557.73	10	4	5	558.35	10	4	4	29.15	1	1	1
P12955	PEPD	PEPD Xaa-Pro dipeptidase Tax_Id=9606	54548	Homo sapiens	555.28	15	5	7	545.68	13	4	4	70.34	5	2	2
P15924	DSP	DSP Desmoplakin Tax_Id=9606	331774	Homo sapiens	545.93	6	4	5	540.63	3	4	5				
P17858	PFKL	PFKL 6-phosphofructokinase, liver type Tax_Id=9606	85018	Homo sapiens	545.22	11	5	5	542.65	11	5	5				
P22234	PAICS	PAICS Multifunctional protein ADE2 Tax_Id=9606	47079	Homo sapiens	539.22	17	4	6	544.31	15	4	4	288.52	11	2	2
Q96IU4	ABHD14B	ABHD14B Alpha/beta hydrolase domain-containing protein 14B Tax_Id=9606	22345	Homo sapiens	538.3	35	4	7	540.04	35	4	5	154.26	6	1	2
P16930	FAH	FAH Fumarylacetoacetase Tax_Id=9606	46374	Homo sapiens	504.56	18	4	7	496.78	13	4	4	288.52	11	2	3
P63241	EIF5A	EIF5A Eukaryotic translation initiation factor 5A-1 Tax_Id=9606	16832	Homo sapiens	503.07	41	3	4	494.81	34	3	4				
P30153	PPP2R1A	PPP2R1A Serine/threonine-protein phosphatase 2A 65 kDa regulatory subunit A alpha is	65308	Homo sapiens	498.78	8	4	5	389.85	8	4	4	110.6	2	1	1
Q7Z3Y8	KRT27	KRT27 Keratin, type I cytoskeletal 27 Tax_Id=9606	49822	Homo sapiens	482.56	13	4	29	289.18	10	2	14	412.79	13	4	15
P45974	USP5	USP5 Ubiquitin carboxyl-terminal hydrolase 5 Tax_Id=9606	95786	Homo sapiens	476.05	9	4	7	479.99	8	4	4	165.36	4	2	3
P07203	GPX1	GPX1 Glutathione peroxidase 1 Tax_Id=9606	22088	Homo sapiens	466.24	24	4	12	287.46	19	3	7	319.02	24	4	5
P60891	PRPS1	PRPS1 Ribose-phosphate pyrophosphokinase 1 Tax_Id=9606	34834	Homo sapiens	449.79	19	4	5	349.17	19	3	3	202.46	11	2	2
P43034	PAFAH1B1	PAFAH1B1 Platelet-activating factor acetylhydrolase IB subunit alpha Tax_Id=9606	46638	Homo sapiens	447.87	15	5	8	440.87	13	4	5	154.26	8	1	2
Q9NRV9	HEBP1	HEBP1 Heme-binding protein 1 Tax_Id=9606	21097	Homo sapiens	446.67	43	4	11	441.39	35	4	7	92.4	35	2	3
P10599	TXN	TXN Thioredoxin Tax_Id=9606	11737	Homo sapiens	439.19	40	4	6	435.36	40	4	5	154.26	21	1	1
P00441	SOD1	SOD1 Superoxide dismutase [Cu-Zn] Tax_Id=9606	15935	Homo sapiens	423.3	28	3	4	423.77	28	3	5				
Q9BS40	LXN	LXN Latexin Tax_Id=9606	25750	Homo sapiens	423.3	17	3	12	423.77	17	3	7	288.52	12	2	5
O14618	CCS	CCS Copper chaperone for superoxide dismutase Tax_Id=9606	29040	Homo sapiens	423.3	20	3	8	421.75	14	3	8				
P16083	NQO2	NQO2 Ribosyldihydronicotinamide dehydrogenase [quinone] Tax_Id=9606	25918	Homo sapiens	423.3	19	3	7	423.77	19	3	3	324.75	19	3	4
P09972	ALDOC	ALDOC Fructose-bisphosphate aldolase C Tax_Id=9606	39455	Homo sapiens	423.3	21	3	7	154.59	14	1	1	422.78	17	3	6
P61088	UBE2N	UBE2N Ubiquitin-conjugating enzyme E2 N Tax_Id=9606	17137	Homo sapiens	423.3	20	3	17	423.77	20	3	9	335.26	20	3	8

P68036	UBE2L3	UBE2L3 Ubiquitin-conjugating enzyme E2 L3 Tax_Id=9606	17861	Homo sapiens	423.3	27	3	3	423.77	27	3	3				
Q13404	UBE2V1	UBE2V1 Ubiquitin-conjugating enzyme E2 variant 1 Tax_Id=9606	16495	Homo sapiens	423.3	29	3	12	423.77	29	3	7	351.69	24	3	5
Q16881	TXNRD1	TXNRD1 Thioredoxin reductase 1, cytoplasmic Tax_Id=9606	70906	Homo sapiens	423.3	6	3	4	423.77	6	3	3	154.26	4	1	1
Q86X55	CARM1	CARM1 Histone-arginine methyltransferase CARM1 Tax_Id=9606	65853	Homo sapiens	423.3	7	3	3	423.77	7	3	3				
Q9HC38	GLOD4	GLOD4 Glyoxalase domain-containing protein 4 Tax_Id=9606	34793	Homo sapiens	423.3	13	3	5	423.77	13	3	4	99.6	3	1	1
Q9UIA9	XPO7	XPO7 Exportin-7 Tax_Id=9606	123907	Homo sapiens	423.3	4	3	4	423.77	4	3	4				
P54725	RAD23A	RAD23A UV excision repair protein RAD23 homolog A Tax_Id=9606	39609	Homo sapiens	419.56	16	3	3	280.35	13	2	2	86.16	6	1	1
P02775	PPBP	PPBP Platelet basic protein Tax_Id=9606	13894	Homo sapiens	419.52	27	3	3	412.51	27	3	3				
P15374	UCHL3	UCHL3 Ubiquitin carboxyl-terminal hydrolase isozyme L3 Tax_Id=9606	26182	Homo sapiens	414.59	16	2	5	404.58	16	2	3	163.92	16	2	2
Q9NTK5	OLA1	OLA1 Obg-like ATPase 1 Tax_Id=9606	44743	Homo sapiens	409.93	10	3	7	277.8	6	2	4	253.85	7	2	3
P00738		HP Haptoglobin Tax_Id=9606	45205	Homo sapiens	407.45	13	3	3	402.11	13	3	3				
O00233	PSMD9	PSMD9 26S proteasome non-ATPase regulatory subunit 9 Tax_Id=9606	24681	Homo sapiens	395.15	17	3	11	387.4	17	3	7	221.38	9	2	4
P35612	ADD2	ADD2 Beta-adducin Tax_Id=9606	80854	Homo sapiens	387.93	7	3	3	392.73	7	3	3				
Q9GZT8	NIF3L1	NIF3L1 Putative GTP cyclohydrolase 1 type 2 NIF3L1 Tax_Id=9606	41968	Homo sapiens	384.73	25	5	7	379.85	25	5	5	39.87	3	1	2
Q9BT73	PSMG3	PSMG3 Proteasome assembly chaperone 3 Tax_Id=9606	13104	Homo sapiens	383.94	43	3	4	380.82	43	3	3	61.08	20	1	1
B9A064	IGLL5	IGLL5 Immunoglobulin lambda-like polypeptide 5 Tax_Id=9606	23063	Homo sapiens	368.6	18	3	4	361.51	18	3	4				
Q5XPI4	RNF123	RNF123 E3 ubiquitin-protein ligase RNF123 Tax_Id=9606	148515	Homo sapiens	359.14	3	3	3	360.84	3	3	3				
P62258	YWHAE	YWHAE 14-3-3 protein epsilon Tax_Id=9606	29173	Homo sapiens	349.8	27	4	4	344.06	23	4	4				
Q13098	GPS1	GPS1 COP9 signalosome complex subunit 1 Tax_Id=9606	55536	Homo sapiens	349.11	12	3	3	338.5	12	3	3				
Q7Z4W1	DCXR	DCXR L-xylulose reductase Tax_Id=9606	25913	Homo sapiens	344.63	14	3	5	200.25	9	2	2	200.05	14	3	3
Q00610	CLTC	CLTC Clathrin heavy chain 1 Tax_Id=9606	191615	Homo sapiens	342.98	3	3	4	342.07	3	3	4				
P26038	MSN	MSN Moesin Tax_Id=9606	67820	Homo sapiens	329.7	10	3	7	325.66	9	3	6	154.26	6	1	1
P25325	MPST	MPST 3-mercaptopyruvate sulfurtransferase Tax_Id=9606	33178	Homo sapiens	324.99	11	3	4	328.36	11	3	3	115.7	4	1	1
P05089	ARG1	ARG1 Arginase-1 Tax_Id=9606	34734	Homo sapiens	318.03	15	3	6	283.31	11	2	2	164.21	10	2	4
P08397	HMBS	HMBS Porphobilinogen deaminase Tax_Id=9606	39330	Homo sapiens	316	13	3	8	316.26	13	3	4	288.52	13	2	4
P58546	MTPN	MTPN Myotrophin Tax_Id=9606	12894	Homo sapiens	295.62	17	2	3	298.77	17	2	3				
P52888	THOP1	THOP1 Thimet oligopeptidase Tax_Id=9606	78839	Homo sapiens	288.86	10	2	2	289.18	10	2	2				
Q96G03	PGM2	PGM2 Phosphoglucomutase-2 Tax_Id=9606	68283	Homo sapiens	288.86	5	2	2	289.18	5	2	2				
P01625		Ig kappa chain V-IV region Len Tax_Id=9606	12640	Homo sapiens	288.86	24	2	2	289.18	24	2	2				
P01742		Ig heavy chain V-I region EU Tax_Id=9606	12472	Homo sapiens	288.86	33	2	2	289.18	16	2	2				
P49189	ALDH9A1	ALDH9A1 4-trimethylaminobutyraldehyde dehydrogenase Tax_Id=9606	53802	Homo sapiens	288.86	17	2	2	289.18	17	2	2				
P53004	BLVRA	BLVRA Biliverdin reductase A Tax_Id=9606	33428	Homo sapiens	288.86	19	2	10	289.18	19	2	4	254.6	8	2	6
Q16658	FSCN1	FSCN1 Fascin Tax_Id=9606	54530	Homo sapiens	288.86	10	2	7	289.18	10	2	5	225.19	6	2	2
Q9BWD1	ACAT2	ACAT2 Acetyl-CoA acetyltransferase, cytosolic Tax_Id=9606	41350	Homo sapiens	288.86	11	2	2	289.18	8	2	2				
Q9H479	FN3K	FN3K Fructosamine-3-kinase Tax_Id=9606	35171	Homo sapiens	288.86	14	2	2	289.18	14	2	2				
Q9NWV4	C1orf123	C1orf123 UPF0587 protein C1orf123 Tax_Id=9606	18048	Homo sapiens	288.86	16	2	9	289.18	16	2	7	82.45	16	1	1
O00321	ETV2	ETV2 ETS translocation variant 2 Tax_Id=9606	36632	Homo sapiens	288.86	3	1	32	289.18	3	1	15	280.41	3	1	17
O14980	XPO1	XPO1 Exportin-1 Tax_Id=9606	123386	Homo sapiens	288.86	3	2	2	289.18	3	2	2				
P02679	FGG	FGG Fibrinogen gamma chain Tax_Id=9606	51511	Homo sapiens	288.86	9	2	2	289.18	9	2	2				
P07477	PRSS1	PRSS1 Trypsin-1 Tax_Id=9606	26558	Homo sapiens	288.86	4	1	21	289.18	4	1	11	265.83	4	1	10
P36959	GMPR	GMPR GMP reductase 1 Tax_Id=9606	37418	Homo sapiens	288.86	32	2	2	289.18	32	2	2				
P48426	PIP4K2A	PIP4K2A Phosphatidylinositol 5-phosphate 4-kinase type-2 alpha Tax_Id=9606	46224	Homo sapiens	288.86	6	2	3	289.18	6	2	2	154.26	2	1	1
P60842	EIF4A1	EIF4A1 Eukaryotic initiation factor 4A-I Tax_Id=9606	46154	Homo sapiens	288.86	13	2	3	289.18	13	2	2	41.64	3	1	1
Q02413	DSG1	DSG1 Desmoglein-1 Tax_Id=9606	113747	Homo sapiens	288.86	3	2	2	289.18	3	2	2				
Q13564	NAE1	NAE1 NEDD8-activating enzyme E1 regulatory subunit Tax_Id=9606	60246	Homo sapiens	288.86	9	2	2	289.18	9	2	2				
Q5TDH0	DDI2	DDI2 Protein DDI1 homolog 2 Tax_Id=9606	44522	Homo sapiens	288.86	13	2	2	289.18	13	2	2				
Q96F85	CNRIP1	CNRIP1 CB1 cannabinoid receptor-interacting protein 1 Tax_Id=9606	18648	Homo sapiens	288.86	17	2	3	289.18	17	2	3				
Q99627	COPS8	COPS8 COP9 signalosome complex subunit 8 Tax_Id=9606	23225	Homo sapiens	288.86	21	2	2	289.18	21	2	2				
Q9UN70	PCDHGC3	PCDHGC3 Protocadherin gamma-C3 Tax_Id=9606	101077	Homo sapiens	288.86	3	1	26	243.11	3	1	15	136.74	2	1	10
P49589	CARS	CARS Cysteine--tRNA ligase, cytoplasmic Tax_Id=9606	85473	Homo sapiens	287.1	6	2	2	286.92	6	2	2				
P04632	CAPNS1	CAPNS1 Calpain small subunit 1 Tax_Id=9606	28315	Homo sapiens	285.36	12	3	4	271.11	12	3	3	55.86	7	1	1
P55072	VCP	VCP Transitional endoplasmic reticulum ATPase Tax_Id=9606	89322	Homo sapiens	277.29	5	3	4	175.28	4	2	2	66.65	2	1	2

Q9C0C9	UBE2O	UBE2O Ubiquitin-conjugating enzyme E2 O Tax_Id=9606	141293	Homo sapiens	276.73	3	2	2	283.3	3	2	2				
P30626	SRI	SRI Sorcin Tax_Id=9606	21676	Homo sapiens	273.16	19	3	9	269.22	19	3	6	111.48	13	2	3
P49720	PSMB3	PSMB3 Proteasome subunit beta type-3 Tax_Id=9606	22948	Homo sapiens	265.69	15	2	2	260.43	15	2	2				
P28074	PSMB5	PSMB5 Proteasome subunit beta type-5 Tax_Id=9606	28480	Homo sapiens	240.33	14	3	3	247.55	14	3	3				
Q13315	ATM	ATM Serine-protein kinase ATM Tax_Id=9606	350688	Homo sapiens	237.49	4	2	5	124.37	3	2	3	72.27	2	1	2
Q9Y230	RUVBL2	RUVBL2 RuvB-like 2 Tax_Id=9606	51156	Homo sapiens	233.05	5	2	2	110.16	2	1	1	71.55	3	1	1
Q07092	COL16A1	COL16A1 Collagen alpha-1(XVI) chain Tax_Id=9606	157751	Homo sapiens	230.42	3	1	5	128.11	3	1	1	127.94	3	1	4
Q5T4D3-2	TMTC4	TMTC4 Isoform 2 of Transmembrane and TPR repeat-containing protein 4 Tax_Id=9606	68352	Homo sapiens	215.06	3	1	46	210.82	3	1	22	80.63	3	1	21
P05109	S100A8	S100A8 Protein S100-A8 Tax_Id=9606	10834	Homo sapiens	214.87	24	2	3	78.13	12	1	1	154.1	12	1	2
P31946	YWHAB	YWHAB 14-3-3 protein beta/alpha Tax_Id=9606	28082	Homo sapiens	214.79	26	3	3	207.72	26	3	3				
P08069	IGF1R	IGF1R Insulin-like growth factor 1 receptor Tax_Id=9606	154793	Homo sapiens	213.93	3	1	17	209.17	3	1	8	101.54	3	1	7
P28072	PSMB6	PSMB6 Proteasome subunit beta type-6 Tax_Id=9606	25357	Homo sapiens	209.79	9	2	2	205.88	9	2	2				
Q13200	PSMD2	PSMD2 26S proteasome non-ATPase regulatory subunit 2 Tax_Id=9606	100199	Homo sapiens	209.55	3	2	2	208.92	3	2	2				
Q15102	PAFAH1B3	PAFAH1B3 Platelet-activating factor acetylhydrolase IB subunit gamma Tax_Id=9606	25734	Homo sapiens	209.35	7	2	6	195.36	7	2	4	42.92	4	1	2
Q9BRA2	TXNDC17	TXNDC17 Thioredoxin domain-containing protein 17 Tax_Id=9606	13940	Homo sapiens	207.57	30	2	3	205.27	30	2	3				
Q9NUW8	TDP1	TDP1 Tyrosyl-DNA phosphodiesterase 1 Tax_Id=9606	68420	Homo sapiens	205.63	7	2	13	157.46	7	2	8	154.26	5	1	5
A0AVI4	TMEM129	TMEM129 Transmembrane protein 129 Tax_Id=9606	40464	Homo sapiens	202.08	5	1	6	147.9	5	1	4	63.78	5	1	1
Q14494	NFE2L1	NFE2L1 Nuclear factor erythroid 2-related factor 1 Tax_Id=9606	84703	Homo sapiens	199.07	4	1	3	62.32	4	1	1	97.15	2	1	2
P04424	ASL	ASL Argininosuccinate lyase Tax_Id=9606	51658	Homo sapiens	197.83	4	2	2	188.44	4	2	2				
Q5H9U9	DDX60L	DDX60L Probable ATP-dependent RNA helicase DDX60-like Tax_Id=9606	197674	Homo sapiens	196.28	2	2	9	183.64	2	2	4	127.51	1	1	5
Q8N3C0-3	ASCC3	ASCC3 Isoform 2 of Activating signal cointegrator 1 complex subunit 3 Tax_Id=9606	13014	Homo sapiens	180.61	15	2	4	147.91	15	1	1	29.29	8	1	3
Q9ULJ1	ODF2L	ODF2L Outer dense fiber protein 2-like Tax_Id=9606	73728	Homo sapiens	175.3	9	1	2					88.75	7	1	2
Q99460	PSMD1	PSMD1 26S proteasome non-ATPase regulatory subunit 1 Tax_Id=9606	105836	Homo sapiens	171.82	4	2	19	154.59	2	1	8	126.33	4	1	10
Q8WZ42	TTN	TTN Titin Tax_Id=9606	3816036	Homo sapiens	162.37	2	5	5	130.39	2	4	4	25.84	1	2	2
Q7L5N1	COPS6	COPS6 COP9 signalosome complex subunit 6 Tax_Id=9606	36163	Homo sapiens	161.98	7	2	2	163.22	7	2	2				
Q9UIQ6	LNPEP	LNPEP Leucyl-cystinyl aminopeptidase Tax_Id=9606	117349	Homo sapiens	158.46	2	1	2	122.28	2	1	2	34.37	2	1	1
P01772		Ig heavy chain V-III region KOL Tax_Id=9606	13718	Homo sapiens	154.43	29	1	1	154.59	29	1	1				
P01781		Ig heavy chain V-III region GAL Tax_Id=9606	12726	Homo sapiens	154.43	9	1	1	154.59	9	1	1				
P04080	CSTB	CSTB Cystatin-B Tax_Id=9606	11139	Homo sapiens	154.43	12	1	1	154.59	12	1	1				
P14174	MIF	MIF Macrophage migration inhibitory factor Tax_Id=9606	12476	Homo sapiens	154.43	10	1	5	154.59	10	1	3	154.26	10	1	2
P14550	AKR1A1	AKR1A1 Alcohol dehydrogenase [NADP(+)] Tax_Id=9606	36573	Homo sapiens	154.43	10	1	1	154.59	10	1	1				
Q15181	PPA1	PPA1 Inorganic pyrophosphatase Tax_Id=9606	32660	Homo sapiens	154.43	3	1	1	154.59	3	1	1				
Q86YZ3	HRNR	HRNR Hornerin Tax_Id=9606	282390	Homo sapiens	154.43	2	1	1	154.59	2	1	1				
Q96KW2	POM121L2	POM121L2 POM121-like protein 2 Tax_Id=9606	109912	Homo sapiens	154.43	3	1	3	154.59	3	1	2				
Q99969	RARRES2	RARRES2 Retinoic acid receptor responder protein 2 Tax_Id=9606	18617	Homo sapiens	154.43	25	1	4	154.59	18	1	3	132.72	14	1	1
Q9BSL1	UBAC1	UBAC1 Ubiquitin-associated domain-containing protein 1 Tax_Id=9606	45338	Homo sapiens	154.43	3	1	1	154.59	3	1	1				
Q9BYZ2	LDHAL6B	LDHAL6B L-lactate dehydrogenase A-like 6B Tax_Id=9606	41943	Homo sapiens	154.43	7	1	2					154.26	7	1	2
Q9H3K6	BOLA2BOLA2E	BOLA2 BolA-like protein 2 Tax_Id=9606	10116	Homo sapiens	154.43	10	1	2	154.59	10	1	2				
Q9NR45	NANS	NANS Sialic acid synthase Tax_Id=9606	40307	Homo sapiens	154.43	10	1	1	154.59	5	1	1				
Q9P0G3	KLK14	KLK14 Kallikrein-14 Tax_Id=9606	29121	Homo sapiens	154.43	16	1	5	96.25	16	1	3	101.83	10	1	2
O00267	SUPT5H	SUPT5H Transcription elongation factor SPT5 Tax_Id=9606	120999	Homo sapiens	154.43	6	1	1	154.59	6	1	1				
O00410	IPO5	IPO5 Importin-5 Tax_Id=9606	123630	Homo sapiens	154.43	2	1	1	154.59	2	1	1				
O75190	DNAJB6	DNAJB6 DnaJ homolog subfamily B member 6 Tax_Id=9606	36087	Homo sapiens	154.43	4	1	1					93.93	4	1	1
O75223	GGCT	GGCT Gamma-glutamylcyclotransferase Tax_Id=9606	21007	Homo sapiens	154.43	19	1	2	154.59	12	1	1	154.26	13	1	1
O75970	MPDZ	MPDZ Multiple PDZ domain protein Tax_Id=9606	221618	Homo sapiens	154.43	4	1	8	154.59	3	1	2	154.26	2	1	6
P01009	SERPINA1	SERPINA1 Alpha-1-antitrypsin Tax_Id=9606	46736	Homo sapiens	154.43	8	1	2	154.59	8	1	2				
P01600		Ig kappa chain V-I region Hau Tax_Id=9606	11670	Homo sapiens	154.43	22	1	1	154.59	22	1	1				
P01614		Ig kappa chain V-II region Cum Tax_Id=9606	12676	Homo sapiens	154.43	11	1	1	154.59	11	1	1				
P01620		Ig kappa chain V-III region SIE Tax_Id=9606	11775	Homo sapiens	154.43	45	1	1	154.59	45	1	1				
P01765		Ig heavy chain V-III region TIL Tax_Id=9606	12352	Homo sapiens	154.43	28	1	1	154.59	28	1	1				
P01766		Ig heavy chain V-III region BRO Tax_Id=9606	13226	Homo sapiens	154.43	25	1	1	154.59	25	1	1				
P02671	FGA	FGA Fibrinogen alpha chain Tax_Id=9606	94973	Homo sapiens	154.43	4	1	1	154.59	4	1	1				

P0CG29	GSTT2	GSTT2 Glutathione S-transferase theta-2 Tax_Id=9606	27506	Homo sapiens	154.43	9	1	3	154.59	9	1	2	64.84	9	1	1
P11171	EPB41	EPB41 Protein 4.1 Tax_Id=9606	97017	Homo sapiens	154.43	8	1	1	154.59	5	1	1				
P17980	PSMC3	PSMC3 26S protease regulatory subunit 6A Tax_Id=9606	49203	Homo sapiens	154.43	4	1	1	154.59	4	1	1				
P20810	CAST	CAST Calpastatin Tax_Id=9606	76572	Homo sapiens	154.43	4	1	5	154.59	4	1	2	60.97	2	1	3
P23381	WARS	WARS Tryptophan--tRNA ligase, cytoplasmic Tax_Id=9606	53165	Homo sapiens	154.43	4	1	1	154.59	4	1	1				
P28066	PSMA5	PSMA5 Proteasome subunit alpha type-5 Tax_Id=9606	26411	Homo sapiens	154.43	7	1	2	154.59	4	1	1	80.57	7	1	1
P30622	CLIP1	CLIP1 CAP-Gly domain-containing linker protein 1 Tax_Id=9606	162246	Homo sapiens	154.43	7	1	20	154.59	6	1	9	85.94	5	1	10
P30740	SERPINB1	SERPINB1 Leukocyte elastase inhibitor Tax_Id=9606	42741	Homo sapiens	154.43	3	1	1	154.59	3	1	1				
P47756	CAPZB	CAPZB F-actin-capping protein subunit beta Tax_Id=9606	31350	Homo sapiens	154.43	13	1	1					154.26	4	1	1
P62158	CALM1CALM2	CALM1 Calmodulin Tax_Id=9606	16837	Homo sapiens	154.43	11	1	3	154.59	11	1	3				
P62195	PSMC5	PSMC5 26S protease regulatory subunit 8 Tax_Id=9606	45626	Homo sapiens	154.43	8	1	1	154.59	6	1	1				
P62491	RAB11A	RAB11A Ras-related protein Rab-11A Tax_Id=9606	24393	Homo sapiens	154.43	5	1	2	122.69	5	1	1	154.26	5	1	1
P62942	FKBP1A	FKBP1A Peptidyl-prolyl cis-trans isomerase FKBP1A Tax_Id=9606	11950	Homo sapiens	154.43	12	1	1	154.59	12	1	1				
P63000	RAC1	RAC1 Ras-related C3 botulinum toxin substrate 1 Tax_Id=9606	21450	Homo sapiens	154.43	8	1	1	154.59	8	1	1				
P63208	SKP1	SKP1 S-phase kinase-associated protein 1 Tax_Id=9606	18658	Homo sapiens	154.43	5	1	1	154.59	5	1	1				
P81605	DCD	DCD Dermcidin Tax_Id=9606	11283	Homo sapiens	154.43	10	1	2	154.59	10	1	1	154.26	10	1	1
P84077	ARF1	ARF1 ADP-ribosylation factor 1 Tax_Id=9606	20696	Homo sapiens	154.43	8	1	1	154.59	8	1	1				
Q08554	DSC1	DSC1 Desmocollin-1 Tax_Id=9606	99987	Homo sapiens	154.43	3	1	1	154.59	3	1	1				
Q13322	GRB10	GRB10 Growth factor receptor-bound protein 10 Tax_Id=9606	67231	Homo sapiens	154.43	4	1	1					96.25	4	1	1
Q14241	TCEB3	TCEB3 Transcription elongation factor B polypeptide 3 Tax_Id=9606	89908	Homo sapiens	154.43	3	1	1	154.59	2	1	1				
Q14974	KPNB1	KPNB1 Importin subunit beta-1 Tax_Id=9606	97170	Homo sapiens	154.43	1	1	2	154.59	1	1	1	22.71	1	1	1
Q15257	PPP2R4	PPP2R4 Serine/threonine-protein phosphatase 2A activator Tax_Id=9606	40667	Homo sapiens	154.43	12	1	3	154.59	10	1	2	104.35	6	1	1
Q5VYK3	ECM29	ECM29 Proteasome-associated protein ECM29 homolog Tax_Id=9606	204291	Homo sapiens	154.43	1	1	10	154.59	1	1	4	107.88	0	1	6
Q6YBV0	SLC36A4	SLC36A4 Proton-coupled amino acid transporter 4 Tax_Id=9606	56157	Homo sapiens	154.43	9	1	3	154.59	9	1	2	51.51	5	1	1
Q6ZMW3	EML6	EML6 Echinoderm microtubule-associated protein-like 6 Tax_Id=9606	217900	Homo sapiens	154.43	3	1	1					86.53	1	1	1
Q8NBJ5	COLGALT1	COLGALT1 Procollagen galactosyltransferase 1 Tax_Id=9606	71636	Homo sapiens	154.43	6	1	12	154.59	4	1	5	96.23	4	1	7
Q8TAC1	RFESD	RFESD Rieske domain-containing protein Tax_Id=9606	17762	Homo sapiens	154.43	10	1	1	154.59	10	1	1				
Q8TBC4	UBA3	UBA3 NEDD8-activating enzyme E1 catalytic subunit Tax_Id=9606	51852	Homo sapiens	154.43	2	1	1	154.59	2	1	1				
Q92665	MRPS31	MRPS31 28S ribosomal protein S31, mitochondrial Tax_Id=9606	45318	Homo sapiens	154.43	3	1	11	154.59	3	1	7	62.13	3	1	3
Q96KS0	EGLN2	EGLN2 Egl nine homolog 2 Tax_Id=9606	43650	Homo sapiens	154.43	2	1	7	154.59	2	1	4	136.16	2	1	3
Q9BT78	COPS4	COPS4 COP9 signalosome complex subunit 4 Tax_Id=9606	46268	Homo sapiens	154.43	6	1	1	154.59	3	1	1				
Q9NZL9	MAT2B	MAT2B Methionine adenosyltransferase 2 subunit beta Tax_Id=9606	37551	Homo sapiens	154.43	9	1	1	154.59	9	1	1				
Q9UNZ2	NSFL1C	NSFL1C NSFL1 cofactor p47 Tax_Id=9606	40572	Homo sapiens	154.43	8	1	1	154.59	8	1	1				
Q9Y265	RUVBL1	RUVBL1 RuvB-like 1 Tax_Id=9606	50228	Homo sapiens	154.43	3	1	2	154.59	3	1	1	119.75	3	1	1
Q9Y3C8	UFC1	UFC1 Ubiquitin-fold modifier-conjugating enzyme 1 Tax_Id=9606	19458	Homo sapiens	154.43	5	1	1	154.59	5	1	1				
Q9Y4E8	USP15	USP15 Ubiquitin carboxyl-terminal hydrolase 15 Tax_Id=9606	112419	Homo sapiens	154.43	2	1	1	154.59	2	1	1				
Q9Y4G8	RAPGEF2	RAPGEF2 Rap guanine nucleotide exchange factor 2 Tax_Id=9606	167417	Homo sapiens	154.43	3	1	3	51.2	3	1	1	92.06	2	1	2
Q8TD26-2	CHD6	CHD6 Isoform 2 of Chromodomain-helicase-DNA-binding protein 6 Tax_Id=9606	41653	Homo sapiens	154.17	5	1	1					87.73	2	1	1
Q5T5Y3	CAMSAP1	CAMSAP1 Calmodulin-regulated spectrin-associated protein 1 Tax_Id=9606	177972	Homo sapiens	153.59	6	1	1					85.02	6	1	1
Q9H706	GAREM	GAREM GRB2-associated and regulator of MAPK protein Tax_Id=9606	97186	Homo sapiens	153.02	5	1	2	154.59	5	1	2				
Q53FA7	TP53I3	TP53I3 Quinone oxidoreductase PIG3 Tax_Id=9606	35536	Homo sapiens	152.04	7	1	2	147.27	7	1	1	62.19	7	1	1
Q9Y2P8	RCL1	RCL1 RNA 3'-terminal phosphate cyclase-like protein Tax_Id=9606	40842	Homo sapiens	151.2	4	1	2	153.48	4	1	1	66.03	4	1	1
Q96GG9	DCUN1D1	DCUN1D1 DCN1-like protein 1 Tax_Id=9606	30124	Homo sapiens	149.07	5	1	1	152.2	5	1	1				
Q15006	EMC2	EMC2 ER membrane protein complex subunit 2 Tax_Id=9606	34833	Homo sapiens	147.27	6	1	5	146.96	6	1	2	26.33	6	1	1
P61566		HERV-K_22q11.21 provirus ancestral Env polyprotein Tax_Id=9606	66584	Homo sapiens	147.03	2	1	6	147.91	2	1	3	79.53	2	1	3
O75052	NOS1AP	NOS1AP Carboxyl-terminal PDZ ligand of neuronal nitric oxide synthase protein Tax_Id=9	56149	Homo sapiens	145.54	8	2	3	147.03	8	2	3				
Q8N715	CCDC185	C1orf65 Uncharacterized protein C1orf65 Tax_Id=9606	72348	Homo sapiens	144.04	11	1	8	63.31	8	1	2	65.24	9	1	2
Q8N3D4	EHBP1L1	EHBP1L1 EH domain-binding protein 1-like protein 1 Tax_Id=9606	161854	Homo sapiens	143.96	3	1	3	137.1	3	1	2	64.73	1	1	1
P35030	PRSS3	PRSS3 Trypsin-3 Tax_Id=9606	32528	Homo sapiens	143.6	4	1	1					80.66	4	1	1
O95714	HERC2	HERC2 E3 ubiquitin-protein ligase HERC2 Tax_Id=9606	527229	Homo sapiens	142.84	2	1	7	105.72	1	1	5	85.04	1	1	2
P20618	PSMB1	PSMB1 Proteasome subunit beta type-1 Tax_Id=9606	26489	Homo sapiens	142.33	21	1	1	145.92	21	1	1				
O15061	SYNM	SYNM Synemin Tax_Id=9606	172767	Homo sapiens	142.3	4	1	9	138.89	3	1	5	62.13	3	1	4

Q68EM7-3	ARHGAP17	ARHGAP17 Isoform 3 of Rho GTPase-activating protein 17 Tax_Id=9606	64267	Homo sapiens	141.05	2	1	4	33.67	2	1	4	80.76	2	1	1
P19883	FST	FST Follistatin Tax_Id=9606	38007	Homo sapiens	140.38	3	1	1	148.14	3	1	1				
Q8TC92	ENOX1	ENOX1 Ecto-NOX disulfide-thiol exchanger 1 Tax_Id=9606	73348	Homo sapiens	139.91	2	1	1	137.6	2	1	1				
P07202	TPO	TPO Thyroid peroxidase Tax_Id=9606	102962	Homo sapiens	139.81	2	1	1	138.39	2	1	1				
Q9NZ56	FMN2	FMN2 Formin-2 Tax_Id=9606	180105	Homo sapiens	138.16	3	1	3	143.11	3	1	3				
O95861	BPNT1	BPNT1 3'(2'),5'-bisphosphate nucleotidase 1 Tax_Id=9606	33392	Homo sapiens	137.66	6	1	1	138.49	6	1	1				
P20339	RAB5A	RAB5A Ras-related protein Rab-5A Tax_Id=9606	23658	Homo sapiens	137.15	5	1	4	122.6	5	1	3	75.52	5	1	1
P17540	CKMT2	CKMT2 Creatine kinase S-type, mitochondrial Tax_Id=9606	47504	Homo sapiens	136.84	19	2	2					71.13	11	2	2
Q7RTT3	SSX9	SSX9 Protein SSX9 Tax_Id=9606	21553	Homo sapiens	136.39	7	2	10	79.84	7	2	6	58.28	7	1	2
Q9P281	BAHCC1	BAHCC1 BAH and coiled-coil domain-containing protein 1 Tax_Id=9606	276932	Homo sapiens	136.21	2	2	8	43.19	2	1	4	68.97	1	2	4
Q8N9B5	JMY	JMY Junction-mediating and -regulatory protein Tax_Id=9606	111445	Homo sapiens	136.13	2	1	1	130.54	2	1	1				
Q71H61	ILDR2	ILDR2 Immunoglobulin-like domain-containing receptor 2 Tax_Id=9606	71200	Homo sapiens	135.31	2	1	7	121.79	2	1	3	75.32	2	1	4
Q96QV6	HIST1H2AA	HIST1H2AA Histone H2A type 1-A Tax_Id=9606	14233	Homo sapiens	134.65	7	1	2	133.48	7	1	2				
Q9HCD5	NCOA5	NCOA5 Nuclear receptor coactivator 5 Tax_Id=9606	65536	Homo sapiens	134.62	2	1	1					78.4	2	1	1
O00151	PDLIM1	PDLIM1 PDZ and LIM domain protein 1 Tax_Id=9606	36071	Homo sapiens	134.41	3	1	3	133.86	3	1	3				
P02763	ORM1	ORM1 Alpha-1-acid glycoprotein 1 Tax_Id=9606	23511	Homo sapiens	132.56	4	1	1	135.45	4	1	1				
P53804	TTC3	TTC3 E3 ubiquitin-protein ligase TTC3 Tax_Id=9606	229869	Homo sapiens	131.28	5	2	2	136.72	5	2	2				
Q92905	COPS5	COPS5 COP9 signalosome complex subunit 5 Tax_Id=9606	37578	Homo sapiens	130.75	9	1	1	124.32	3	1	1				
P50226	SULT1A2	SULT1A2 Sulfotransferase 1A2 Tax_Id=9606	34309	Homo sapiens	130.51	8	1	2					70.89	8	1	1
Q07864	POLE	POLE DNA polymerase epsilon catalytic subunit A Tax_Id=9606	261518	Homo sapiens	130.26	2	1	1	129.84	1	1	1				
Q05215	EGR4	EGR4 Early growth response protein 4 Tax_Id=9606	61623	Homo sapiens	130.24	6	1	4	132.53	6	1	4				
Q9P2Q2	FRMD4A	FRMD4A FERM domain-containing protein 4A Tax_Id=9606	115458	Homo sapiens	129.55	3	1	1					73.28	2	1	1
Q16821	PPP1R3A	PPP1R3A Protein phosphatase 1 regulatory subunit 3A Tax_Id=9606	125766	Homo sapiens	129.21	1	1	4	57.36	1	1	2	73.49	1	1	1
Q13510	ASAH1	ASAH1 Acid ceramidase Tax_Id=9606	44659	Homo sapiens	126.36	3	1	2					71.79	3	1	2
P27708	CAD	CAD CAD protein Tax_Id=9606	242984	Homo sapiens	125.83	1	1	1	118.13	1	1	1				
P61086	UBE2K	UBE2K Ubiquitin-conjugating enzyme E2 K Tax_Id=9606	22406	Homo sapiens	125.82	14	1	1	133.24	14	1	1				
Q8WUM4	PDCD6IP	PDCD6IP Programmed cell death 6-interacting protein Tax_Id=9606	96023	Homo sapiens	124.79	7	3	3	118.35	4	3	3				
Q8N1I0	DOCK4	DOCK4 Dedicator of cytokinesis protein 4 Tax_Id=9606	225206	Homo sapiens	123.44	6	1	2	122.52	3	1	1	56.01	4	1	1
Q01668	CACNA1D	CACNA1D Voltage-dependent L-type calcium channel subunit alpha-1D Tax_Id=9606	245141	Homo sapiens	122.62	2	1	3	79.53	2	1	1	68.17	2	1	2
Q16222	UAP1	UAP1 UDP-N-acetylhexosamine pyrophosphorylase Tax_Id=9606	58769	Homo sapiens	121.16	5	1	1	109.79	3	1	1				
Q5SXM2	SNAPC4	SNAPC4 snRNA-activating protein complex subunit 4 Tax_Id=9606	159433	Homo sapiens	120.31	4	1	4	127.23	3	1	3	31.08	3	1	1
P31944	CASP14	CASP14 Caspase-14 Tax_Id=9606	27679	Homo sapiens	118.43	4	1	1	108.97	4	1	1				
Q9P2D8	UNC79	UNC79 Protein unc-79 homolog Tax_Id=9606	295327	Homo sapiens	117.98	2	1	3	29.65	1	1	2	59.88	1	1	2
Q8IWC1	MAP7D3	MAP7D3 MAP7 domain-containing protein 3 Tax_Id=9606	98429	Homo sapiens	117.47	4	1	1	114.09	4	1	1				
Q9H299	SH3BGRL3	SH3BGRL3 SH3 domain-binding glutamic acid-rich-like protein 3 Tax_Id=9606	10437	Homo sapiens	117.44	11	1	1	116.45	11	1	1				
P04433		Ig kappa chain V-III region VG (Fragment) Tax_Id=9606	12575	Homo sapiens	115.39	8	1	1	118.19	8	1	1				
Q149M9	NWD1	NWD1 NACHT and WD repeat domain-containing protein 1 Tax_Id=9606	174552	Homo sapiens	115.32	3	1	1	116.28	3	1	1				
P06396	GSN	GSN Gelsolin Tax_Id=9606	85697	Homo sapiens	114.96	2	1	3	111.49	2	1	2				
Q9Y462	ZNF711	ZNF711 Zinc finger protein 711 Tax_Id=9606	86245	Homo sapiens	114.71	2	1	5	89.3	2	1	3	31.75	2	1	2
O43396	TXNL1	TXNL1 Thioredoxin-like protein 1 Tax_Id=9606	32251	Homo sapiens	113.89	10	2	3	49.04	2	1	1	55.8	10	2	2
Q9NZJ4	SACS	SACS Sacsin Tax_Id=9606	521127	Homo sapiens	112.79	4	1	1	108.13	3	1	1				
Q9Y3B8	REXO2	REXO2 Oligoribonuclease, mitochondrial Tax_Id=9606	26832	Homo sapiens	112.33	3	1	3	108.63	3	1	3				
Q9Y4R7	TTLL3	TTLL3 Tubulin monoglycylase TTLL3 Tax_Id=9606	87414	Homo sapiens	110.51	6	1	2					63.41	2	1	2
Q7Z570	ZNF804A	ZNF804A Zinc finger protein 804A Tax_Id=9606	136888	Homo sapiens	110.47	4	1	2					63.59	4	1	2
P42566	EPS15	EPS15 Epidermal growth factor receptor substrate 15 Tax_Id=9606	98655	Homo sapiens	110.38	4	1	3	79.28	4	1	1	58.29	2	1	2
Q92833	JARID2	JARID2 Protein Jumonji Tax_Id=9606	138734	Homo sapiens	109.04	4	1	1	110.82	3	1	1				
Q9H307	PNN	PNN Pinin Tax_Id=9606	81613	Homo sapiens	108.82	6	1	4	69.35	6	1	2	60.79	2	1	2
Q99965	ADAM2	ADAM2 Disintegrin and metalloproteinase domain-containing protein 2 Tax_Id=9606	82457	Homo sapiens	108.54	7	1	1	107.89	7	1	1				
Q12981	BNIP1	BNIP1 Vesicle transport protein SEC20 Tax_Id=9606	26132	Homo sapiens	108.27	18	1	4	98.43	18	1	3	33.34	18	1	1
Q9Y616	IRAK3	IRAK3 Interleukin-1 receptor-associated kinase 3 Tax_Id=9606	67766	Homo sapiens	108.01	5	1	1					62.65	5	1	1
Q8IW41	MAPKAPK5	MAPKAPK5 MAP kinase-activated protein kinase 5 Tax_Id=9606	54220	Homo sapiens	107.23	4	1	1	99	4	1	1				
Q8N3K9	CMYA5	CMYA5 Cardiomyopathy-associated protein 5 Tax_Id=9606	449211	Homo sapiens	106.49	4	1	1	103.42	3	1	1				

Q6SZW1	SARM1	SARM1 Sterile alpha and TIR motif-containing protein 1 Tax_Id=9606	79388	Homo sapiens	106.44	6	1	1	108.44	6	1	1				
Q7Z5Y7	KCTD20	KCTD20 BTB/POZ domain-containing protein KCTD20 Tax_Id=9606	47480	Homo sapiens	106.35	2	1	1	102.35	2	1	1				
Q6N022	TENM4	TENM4 Teneurin-4 Tax_Id=9606	307957	Homo sapiens	106.26	2	1	1	102.23	2	1	1				
Q96SQ5	ZNF587	ZNF587 Zinc finger protein 587 Tax_Id=9606	65622	Homo sapiens	106.2	4	1	2	40.12	4	1	1	59.81	4	1	1
Q86YQ8	CPNE8	CPNE8 Copine-8 Tax_Id=9606	63107	Homo sapiens	104.58	4	1	2					54.84	1	1	2
P55285	CDH6	CDH6 Cadherin-6 Tax_Id=9606	88308	Homo sapiens	104.04	3	1	2	102.17	3	1	2				
P10114	RAP2A	RAP2A Ras-related protein Rap-2a Tax_Id=9606	20615	Homo sapiens	103.71	6	1	1	103.87	6	1	1				
P09493	TPM1	TPM1 Tropomyosin alpha-1 chain Tax_Id=9606	32708	Homo sapiens	103.33	5	1	1	107.18	5	1	1				
Q96GP6	SCARF2	SCARF2 Scavenger receptor class F member 2 Tax_Id=9606	92479	Homo sapiens	103.09	3	1	2					59.28	3	1	2
P42892-4	ECE1	ECE1 Isoform D of Endothelin-converting enzyme 1 Tax_Id=9606	87022	Homo sapiens	102.37	4	1	2	98.11	4	1	1				
Q9BUQ8	DDX23	DDX23 Probable ATP-dependent RNA helicase DDX23 Tax_Id=9606	95582	Homo sapiens	102.11	6	1	4	93.9	6	1	2	55.96	3	1	2
Q9BXJ9	NAA15	NAA15 N-alpha-acetyltransferase 15, NatA auxiliary subunit Tax_Id=9606	101272	Homo sapiens	102.03	4	1	13	100.47	2	1	7	45.6	4	1	5
Q8N573	OXR1	OXR1 Oxidation resistance protein 1 Tax_Id=9606	97970	Homo sapiens	101.43	4	1	6	68.17	4	1	4	55.71	3	1	2
O43542	XRCC3	XRCC3 DNA repair protein XRCC3 Tax_Id=9606	37849	Homo sapiens	101.21	6	1	1	103.23	6	1	1				
P07311	ACYP1	ACYP1 Acylphosphatase-1 Tax_Id=9606	11260	Homo sapiens	100.99	20	2	2	103.25	20	2	2				
Q6NT04	TIGD7	TIGD7 Tigger transposable element-derived protein 7 Tax_Id=9606	63236	Homo sapiens	100.58	3	1	10	89.25	3	1	6	57.56	3	1	4
P07098-3	LIPF	LIPF Isoform 3 of Gastric triacylglycerol lipase Tax_Id=9606	46361	Homo sapiens	100.45	2	1	5	68.43	2	1	2	56.95	2	1	3
Q15643	TRIP11	TRIP11 Thyroid receptor-interacting protein 11 Tax_Id=9606	227586	Homo sapiens	100.16	2	1	1	97.22	1	1	1				
P16144	ITGB4	ITGB4 Integrin beta-4 Tax_Id=9606	202167	Homo sapiens	99.87	1	1	2	100.4	1	1	2				
P10523	SAG	SAG S-arrestin Tax_Id=9606	45119	Homo sapiens	99.69	3	1	1	93.4	3	1	1				
Q8TBB6	SLC7A14	SLC7A14 Probable cationic amino acid transporter Tax_Id=9606	84051	Homo sapiens	99.32	4	1	1	94.43	4	1	1				
Q6ZRP7	QSOX2	QSOX2 Sulfhydryl oxidase 2 Tax_Id=9606	77528	Homo sapiens	97.86	4	1	1	103.17	4	1	1				
Q14587	ZNF268	ZNF268 Zinc finger protein 268 Tax_Id=9606	108374	Homo sapiens	97.86	3	1	38	91.67	3	1	22	47.86	1	1	12
P00450		CP Ceruloplasmin Tax_Id=9606	122205	Homo sapiens	97.51	1	1	1	91.66	1	1	1				
Q5HYA8	TMEM67	TMEM67 Meckelin Tax_Id=9606	111745	Homo sapiens	97.28	6	1	1	96.85	4	1	1				
Q9H9A6	LRRC40	LRRC40 Leucine-rich repeat-containing protein 40 Tax_Id=9606	68250	Homo sapiens	96.96	7	1	1	97.18	7	1	1				
Q86W92	PPFIBP1	PPFIBP1 Liprin-beta-1 Tax_Id=9606	114024	Homo sapiens	96.84	5	1	1					56.62	3	1	1
Q8NHS4	CLHC1	CLHC1 Clathrin heavy chain linker domain-containing protein 1 Tax_Id=9606	67249	Homo sapiens	96.56	3	1	2	98.09	3	1	1	53.63	3	1	1
Q9UKT6	FBXL21	FBXL21 F-box/LRR-repeat protein 21 Tax_Id=9606	49151	Homo sapiens	96.45	3	1	25	88.16	3	1	12	57.19	3	1	10
Q9UKA2	FBXL4	FBXL4 F-box/LRR-repeat protein 4 Tax_Id=9606	70097	Homo sapiens	95.96	8	1	4	41.04	8	1	1	50.34	4	1	3
Q6ZR52	ZNF493	ZNF493 Zinc finger protein 493 Tax_Id=9606	75341	Homo sapiens	94.34	5	1	1	97.49	3	1	1				
P01011	SERPINA3	SERPINA3 Alpha-1-antichymotrypsin Tax_Id=9606	47650	Homo sapiens	94.25	4	1	1	91.79	4	1	1				
Q96F44	TRIM11	TRIM11 E3 ubiquitin-protein ligase TRIM11 Tax_Id=9606	52774	Homo sapiens	93.87	10	2	2	32.43	7	1	1	44.71	8	1	1
Q9Y2D5-4	AKAP2	AKAP2 Isoform 2 of A-kinase anchor protein 2 Tax_Id=9606	122071	Homo sapiens	93.24	2	1	3					53.86	1	1	3
Q96ID5	IGSF21	IGSF21 Immunoglobulin superfamily member 21 Tax_Id=9606	51835	Homo sapiens	92.79	7	1	1					51.55	4	1	1
Q9NXV2	KCTD5	KCTD5 BTB/POZ domain-containing protein KCTD5 Tax_Id=9606	26092	Homo sapiens	92.75	6	1	1					55.75	6	1	1
P06730	EIF4E	EIF4E Eukaryotic translation initiation factor 4E Tax_Id=9606	25097	Homo sapiens	92.36	6	1	1	91.26	6	1	1				
Q2QD12	RPEL1	RPEL1 Ribulose-phosphate 3-epimerase-like protein 1 Tax_Id=9606	25022	Homo sapiens	91.66	11	1	3	96.32	3	1	2	48.01	11	1	1
P13796	LCP1	LCP1 Plastin-2 Tax_Id=9606	70288	Homo sapiens	91.1	3	1	1	96.68	3	1	1				
Q13751	LAMB3	LAMB3 Laminin subunit beta-3 Tax_Id=9606	129573	Homo sapiens	90.16	1	1	1					50.61	1	1	1
Q9BV73	CEP250	CEP250 Centrosome-associated protein CEP250 Tax_Id=9606	281137	Homo sapiens	90.04	2	1	7	92.56	1	1	4	33.27	1	1	3
P28161	GSTM2	GSTM2 Glutathione S-transferase Mu 2 Tax_Id=9606	25744	Homo sapiens	89.72	4	1	1	81.75	4	1	1				
Q5VU65	NUP210L	NUP210L Nuclear pore membrane glycoprotein 210-like Tax_Id=9606	210605	Homo sapiens	88.88	2	1	2	34.75	2	1	1	52.36	1	1	1
Q96T37	RBM15	RBM15 Putative RNA-binding protein 15 Tax_Id=9606	107188	Homo sapiens	88.6	4	1	1	82.43	4	1	1				
Q9HBH7	BEX1	BEX1 Protein BEX1 Tax_Id=9606	14859	Homo sapiens	88.47	12	1	1	83.35	10	1	1				
Q8NBM8	PCYOX1L	PCYOX1L Prenylcysteine oxidase-like Tax_Id=9606	54646	Homo sapiens	88.17	5	1	1	90.65	5	1	1				
Q8IVL1	NAV2	NAV2 Neuron navigator 2 Tax_Id=9606	268167	Homo sapiens	87.15	1	1	7	38.25	1	1	3	50.58	1	1	3
Q9NX74	DUS2	DUS2 tRNA-dihydrouridine(20) synthase [NAD(P)+]-like Tax_Id=9606	55050	Homo sapiens	86.91	6	1	1					44.78	3	1	1
P0CJ78	ZNF865	ZNF865 Zinc finger protein 865 Tax_Id=9606	111077	Homo sapiens	86.61	6	2	2					38.05	4	2	2
Q5QP82	DCAF10	DCAF10 DDB1- and CUL4-associated factor 10 Tax_Id=9606	60582	Homo sapiens	86.38	1	1	5	90.1	1	1	3	46.68	1	1	2
Q96NR3	PTCHD1	PTCHD1 Patched domain-containing protein 1 Tax_Id=9606	101341	Homo sapiens	85.86	2	1	1	84.03	2	1	1				
P51582	P2RY4	P2RY4 P2Y purinoceptor 4 Tax_Id=9606	40963	Homo sapiens	85.73	7	1	5					47.81	7	1	4

Q9Y6N9	USH1C	USH1C Harmonin Tax_Id=9606	62210	Homo sapiens	85.3	3	1	1	86.95	3	1	1				
Q9C0C7	AMBRA1	AMBRA1 Activating molecule in BECN1-regulated autophagy protein 1 Tax_Id=9606	142507	Homo sapiens	84.93	5	1	2	28.91	4	1	1	42.02	3	1	1
Q8N4C6	NIN	NIN Ninein Tax_Id=9606	243249	Homo sapiens	84.74	3	1	1					48.76	3	1	1
Q6WRI0	IGSF10	IGSF10 Immunoglobulin superfamily member 10 Tax_Id=9606	290838	Homo sapiens	83.92	2	1	1					49.06	1	1	1
Q9NUL5	C19orf66	C19orf66 UPF0515 protein C19orf66 Tax_Id=9606	33110	Homo sapiens	83.79	3	1	1	84.63	3	1	1				
Q86XZ4	SPATS2	SPATS2 Spermatogenesis-associated serine-rich protein 2 Tax_Id=9606	59544	Homo sapiens	82.89	5	1	1	81.43	5	1	1				
Q5VZ89	DENND4C	DENND4C DENN domain-containing protein 4C Tax_Id=9606	186857	Homo sapiens	82.8	3	1	1					47.48	2	1	1
P49207	RPL34	RPL34 60S ribosomal protein L34 Tax_Id=9606	13292	Homo sapiens	82.51	9	1	1	80.7	9	1	1				
Q9BTZ2-4	DHRS4	DHRS4 Isoform 4 of Dehydrogenase/reductase SDR family member 4 Tax_Id=9606	25882	Homo sapiens	82.07	5	1	1	77.25	5	1	1				
O43451	MGAM	MGAM Maltase-glucoamylase, intestinal Tax_Id=9606	209852	Homo sapiens	81.68	2	1	1	83.47	2	1	1				
P35318	ADM	ADM ADM Tax_Id=9606	20420	Homo sapiens	80.97	5	1	1	84.11	5	1	1				
O60437	PPL	PPL Periplakin Tax_Id=9606	204747	Homo sapiens	80.79	2	1	1					47.25	2	1	1
O60294	LCMT2	LCMT2 tRNA wybutosine-synthesizing protein 4 Tax_Id=9606	75601	Homo sapiens	80.04	3	1	1	79.75	3	1	1				
O14647	CHD2	CHD2 Chromodomain-helicase-DNA-binding protein 2 Tax_Id=9606	211343	Homo sapiens	79.9	2	1	1	72.9	2	1	1				
O75912	DGKI	DGKI Diacylglycerol kinase iota Tax_Id=9606	116997	Homo sapiens	79.24	2	1	1	67.86	2	1	1				
Q9UL51	HCN2	HCN2 Potassium/sodium hyperpolarization-activated cyclic nucleotide-gated channel 2 T	96950	Homo sapiens	78.8	2	1	2					44.99	2	1	1
O75165	DNAJC13	DNAJC13 DnaJ homolog subfamily C member 13 Tax_Id=9606	254415	Homo sapiens	78.43	2	1	5	68.92	2	1	2	45.97	1	1	2
O95672	ECEL1	ECEL1 Endothelin-converting enzyme-like 1 Tax_Id=9606	87791	Homo sapiens	78.4	7	1	1	79.34	7	1	1				
Q8NG50	RDM1	RDM1 RAD52 motif-containing protein 1 Tax_Id=9606	31969	Homo sapiens	78.19	5	1	1	74.1	5	1	1				
P06702	S100A9	S100A9 Protein S100-A9 Tax_Id=9606	13242	Homo sapiens	78.16	11	1	1					45.47	11	1	1
O75325	LRRN2	LRRN2 Leucine-rich repeat neuronal protein 2 Tax_Id=9606	78858	Homo sapiens	78.14	6	2	5	77.38	5	2	4	39.05	6	1	1
P56537	EIF6	EIF6 Eukaryotic translation initiation factor 6 Tax_Id=9606	26599	Homo sapiens	77.8	4	1	1	77.3	4	1	1				
Q8NFR7-4	CCDC148	CCDC148 Isoform 4 of Coiled-coil domain-containing protein 148 Tax_Id=9606	35793	Homo sapiens	77.71	5	1	1	72.98	5	1	1				
Q8N2Y8	RUSC2	RUSC2 Iporin Tax_Id=9606	161225	Homo sapiens	77.67	4	1	2					42.79	3	1	2
Q7Z478	DHX29	DHX29 ATP-dependent RNA helicase DHX29 Tax_Id=9606	155235	Homo sapiens	77.53	3	1	1					44.65	2	1	1
O95633	FSTL3	FSTL3 Follistatin-related protein 3 Tax_Id=9606	27663	Homo sapiens	77.43	5	1	1	80.7	5	1	1				
P05090	APOD	APOD Apolipoprotein D Tax_Id=9606	21275	Homo sapiens	77.41	6	1	1	81.06	6	1	1				
Q9NS62	THSD1	THSD1 Thrombospondin type-1 domain-containing protein 1 Tax_Id=9606	94584	Homo sapiens	77.28	4	1	1	80.43	4	1	1				
Q8TAQ5	ZNF420	ZNF420 Zinc finger protein 420 Tax_Id=9606	80247	Homo sapiens	76.99	3	1	3	76.41	3	1	2	28.33	3	1	1
Q00013	MPP1	MPP1 55 kDa erythrocyte membrane protein Tax_Id=9606	52296	Homo sapiens	76.44	5	1	1					42.57	5	1	1
Q8NH19	OR10AG1	OR10AG1 Olfactory receptor 10AG1 Tax_Id=9606	34104	Homo sapiens	76.4	3	1	1	84.66	3	1	1				
Q8N6Y0	USHBP1	USHBP1 Usher syndrome type-1C protein-binding protein 1 Tax_Id=9606	76068	Homo sapiens	76.32	2	1	1	74.22	2	1	1				
Q14683	SMC1A	SMC1A Structural maintenance of chromosomes protein 1A Tax_Id=9606	143233	Homo sapiens	76.14	4	1	3	80.16	3	1	4				
Q9BW92	TARS2	TARS2 Threonine--tRNA ligase, mitochondrial Tax_Id=9606	81036	Homo sapiens	74.28	1	1	2					43.33	1	1	2
P45985	MAP2K4	MAP2K4 Dual specificity mitogen-activated protein kinase kinase 4 Tax_Id=9606	44287	Homo sapiens	73.2	10	1	1	92.18	10	1	1				
Q86UQ4-2	ABCA13	ABCA13 Isoform 2 of ATP-binding cassette sub-family A member 13 Tax_Id=9606	36920	Homo sapiens	72.62	10	1	2	69.37	10	1	2				
O60268	KIAA0513	KIAA0513 Uncharacterized protein KIAA0513 Tax_Id=9606	46638	Homo sapiens	72.34	6	1	4	60.17	3	1	1	41.32	6	1	2
P0CL83	STAG3L1	STAG3L1 Putative STAG3-like protein 1 Tax_Id=9606	23774	Homo sapiens	72.17	6	1	1	72.05	6	1	1				
Q7L1W4	LRRC8D	LRRC8D Leucine-rich repeat-containing protein 8D Tax_Id=9606	98201	Homo sapiens	72.13	1	1	1					42.42	1	1	1
Q9GZN2	TGIF2	TGIF2 Homeobox protein TGIF2 Tax_Id=9606	25878	Homo sapiens	71.57	7	1	3	22.21	7	1	1	40.88	7	1	1
Q8N6G6-5	ADAMTSL1	ADAMTSL1 Isoform 5 of ADAMTS-like protein 1 Tax_Id=9606	27468	Homo sapiens	71.51	3	1	4	60.35	3	1	1	39.94	3	1	2
Q9H082	RAB33B	RAB33B Ras-related protein Rab-33B Tax_Id=9606	25717	Homo sapiens	71.39	8	1	1	74.59	8	1	1				
Q15149	PLEC	PLEC Plectin Tax_Id=9606	531791	Homo sapiens	71.33	2	1	3	77.97	1	1	2	32.92	1	1	1
Q9HBG4	ATP6V0A4	ATP6V0A4 V-type proton ATPase 116 kDa subunit a isoform 4 Tax_Id=9606	96386	Homo sapiens	71.32	1	1	1	65.64	1	1	1				
Q8WUQ7	CACTIN	CACTIN Cactin Tax_Id=9606	88701	Homo sapiens	71.01	1	1	1	73.61	1	1	1				
Q8NA82	Mar-10	MARCH10 Probable E3 ubiquitin-protein ligase MARCH10 Tax_Id=9606	90511	Homo sapiens	70.2	2	1	1					41.02	2	1	1
Q13490	BIRC2	BIRC2 Baculoviral IAP repeat-containing protein 2 Tax_Id=9606	69899	Homo sapiens	70.07	3	1	1	65.31	3	1	1				
Q9ULL0	KIAA1210	KIAA1210 Uncharacterized protein KIAA1210 Tax_Id=9606	187021	Homo sapiens	69.95	2	1	4	26.42	2	1	1	34.42	1	1	2
Q13330	MTA1	MTA1 Metastasis-associated protein MTA1 Tax_Id=9606	80786	Homo sapiens	69.65	2	1	1					38.24	2	1	1
Q7Z7L7	ZER1	ZER1 Protein zer-1 homolog Tax_Id=9606	88169	Homo sapiens	69.61	2	1	1	67.98	2	1	1				
Q5T5X7	BEND3	BEND3 BEN domain-containing protein 3 Tax_Id=9606	94474	Homo sapiens	69.37	2	1	1	67.38	2	1	1				
Q06136	KDSR	KDSR 3-ketodihydrosphingosine reductase Tax_Id=9606	36187	Homo sapiens	69.35	12	1	1	71.4	12	1	1				

Q8N6K7	SAMD3	SAMD3 Sterile alpha motif domain-containing protein 3 Tax_Id=9606	61236	Homo sapiens	68.98	3	1	1					39.07	3	1	1
Q15208	STK38	STK38 Serine/threonine-protein kinase 38 Tax_Id=9606	54190	Homo sapiens	68.24	9	1	1	71.63	9	1	1				
Q9ULE0	WWC3	WWC3 Protein WWC3 Tax_Id=9606	122676	Homo sapiens	68.03	6	1	1					38.94	4	1	1
P12821	ACE	ACE Angiotensin-converting enzyme Tax_Id=9606	149715	Homo sapiens	66.76	1	1	1					37.4	1	1	1
Q9Y2G0	EFR3B	EFR3B Protein EFR3 homolog B Tax_Id=9606	92486	Homo sapiens	66.64	3	1	1					35.12	3	1	1
Q96A65	EXOC4	EXOC4 Exocyst complex component 4 Tax_Id=9606	110498	Homo sapiens	66.58	7	1	1	67.63	5	1	1				
Q8N6N2	TTC9B	TTC9B Tetratricopeptide repeat protein 9B Tax_Id=9606	25932	Homo sapiens	66.23	7	1	1	64.93	7	1	1				
Q8IZU0	FAM9B	FAM9B Protein FAM9B Tax_Id=9606	22437	Homo sapiens	65.43	15	1	1					37.76	15	1	1
P29218	IMPA1	IMPA1 Inositol monophosphatase 1 Tax_Id=9606	30188	Homo sapiens	64.15	9	1	1					35.52	9	1	1
P20340-4	RAB6A	RAB6A Isoform 4 of Ras-related protein Rab-6A Tax_Id=9606	19934	Homo sapiens	63.55	11	1	2	54.57	11	1	1	28.12	11	1	1
Q08AG5	ZNF844	ZNF844 Zinc finger protein 844 Tax_Id=9606	76934	Homo sapiens	63.45	6	2	5	58.16	5	2	2	25.29	6	2	2
P49257	LMAN1	LMAN1 Protein ERGIC-53 Tax_Id=9606	57549	Homo sapiens	63.36	2	1	1					36.15	2	1	1
P15498	VAV1	VAV1 Proto-oncogene vav Tax_Id=9606	98314	Homo sapiens	63.24	3	1	2	36.89	3	1	1	33.51	3	1	1
Q86UR5-12	RIMS1	RIMS1 Isoform 12 of Regulating synaptic membrane exocytosis protein 1 Tax_Id=9606	112988	Homo sapiens	63.24	2	1	2	62.11	2	1	1	24.63	2	1	1
Q8TBM8	DNAJB14	DNAJB14 DnaJ homolog subfamily B member 14 Tax_Id=9606	42515	Homo sapiens	63.16	14	1	1	62.61	14	1	1				
Q702N8	XIRP1	XIRP1 Xin actin-binding repeat-containing protein 1 Tax_Id=9606	198561	Homo sapiens	63.1	2	1	1					35.11	2	1	1
O95342	ABCB11	ABCB11 Bile salt export pump Tax_Id=9606	146407	Homo sapiens	62.96	1	1	1	57.61	1	1	1				
Q6NV75	GPR153	GPR153 Probable G-protein coupled receptor 153 Tax_Id=9606	65361	Homo sapiens	62.92	5	1	1					30.91	5	1	1
O75891	ALDH1L1	ALDH1L1 Cytosolic 10-formyltetrahydrofolate dehydrogenase Tax_Id=9606	98829	Homo sapiens	62.68	4	1	1	62.48	3	1	1				
P19823	ITIH2	ITIH2 Inter-alpha-trypsin inhibitor heavy chain H2 Tax_Id=9606	106463	Homo sapiens	62.67	4	1	1	65.24	4	1	1				
P26639	TARS	TARS Threonine--tRNA ligase, cytoplasmic Tax_Id=9606	83435	Homo sapiens	62.37	6	1	1	56.47	3	1	1				
A6NCS4	NKX2-6	NKX2-6 Homeobox protein Nkx-2.6 Tax_Id=9606	32121	Homo sapiens	62.27	7	1	1	62.15	7	1	1				
Q9BX10	GTPBP2	GTPBP2 GTP-binding protein 2 Tax_Id=9606	65768	Homo sapiens	62.19	2	1	1	65.73	2	1	1				
Q8N8U3	ZCCHC5	ZCCHC5 Zinc finger CCHC domain-containing protein 5 Tax_Id=9606	52817	Homo sapiens	62.16	9	1	1					39.1	9	1	1
P01833	PIGR	PIGR Polymeric immunoglobulin receptor Tax_Id=9606	83283	Homo sapiens	62.12	3	1	2	60.44	3	1	2				
O43173	ST8SIA3	ST8SIA3 Sia-alpha-2,3-Gal-beta-1,4-GlcNAc-R:alpha 2,8-sialyltransferase Tax_Id=9606	43969	Homo sapiens	62.01	10	1	1					34.81	10	1	1
Q6PCE3	PGM2L1	PGM2L1 Glucose 1,6-bisphosphate synthase Tax_Id=9606	70441	Homo sapiens	61.91	6	1	1					35.14	5	1	1
Q02383	SEMG2	SEMG2 Semenogelin-2 Tax_Id=9606	65444	Homo sapiens	61.49	4	1	1					36.96	4	1	1
Q00872	MYBPC1	MYBPC1 Myosin-binding protein C, slow-type Tax_Id=9606	128294	Homo sapiens	61.36	3	1	2	60.36	1	1	2				
O94979	SEC31A	SEC31A Protein transport protein Sec31A Tax_Id=9606	133014	Homo sapiens	61.15	3	1	2	65.7	3	1	2				
Q6PIW4	FIGNL1	FIGNL1 Fidgetin-like protein 1 Tax_Id=9606	74077	Homo sapiens	60.98	2	1	4	64.29	2	1	3	24.87	2	1	1
Q8WWX9	SELM	SELM Selenoprotein M Tax_Id=9606	16231	Homo sapiens	60.92	10	1	1	57.47	10	1	1				
A4FU49	SH3D21	SH3D21 SH3 domain-containing protein 21 Tax_Id=9606	70519	Homo sapiens	60.88	5	1	3	63.24	4	1	2	26.09	5	1	1
Q9Y6K5	OAS3	OAS3 2'-5'-oligoadenylate synthase 3 Tax_Id=9606	121170	Homo sapiens	60.81	2	1	1	56.55	2	1	1				
O75911	DHRS3	DHRS3 Short-chain dehydrogenase/reductase 3 Tax_Id=9606	33548	Homo sapiens	60.47	10	1	1	52.3	10	1	1				
O15263	DEFB4ADEFB4	DEFB4A Beta-defensin 4A Tax_Id=9606	7037	Homo sapiens	60.37	22	1	6	49.17	22	1	2	36.97	22	1	4
Q969L4	LSM10	LSM10 U7 snRNA-associated Sm-like protein LSm10 Tax_Id=9606	14080	Homo sapiens	60.09	15	1	1	59.07	15	1	1				
Q9BVM4	GGACT	GGACT Gamma-glutamylaminecyclotransferase Tax_Id=9606	17328	Homo sapiens	60.04	5	1	1	51.99	5	1	1				
P19634	SLC9A1	SLC9A1 Sodium/hydrogen exchanger 1 Tax_Id=9606	90763	Homo sapiens	60.02	3	1	1	58.39	3	1	1				
Q9Y217	MTMR6	MTMR6 Myotubularin-related protein 6 Tax_Id=9606	71968	Homo sapiens	59.86	8	1	3	27.73	8	1	1	28.97	7	1	2
P32927	CSF2RB	CSF2RB Cytokine receptor common subunit beta Tax_Id=9606	97335	Homo sapiens	59.81	3	1	1					38.87	3	1	1
Q96PQ0	SORCS2	SORCS2 VPS10 domain-containing receptor SorCS2 Tax_Id=9606	128151	Homo sapiens	59.52	2	1	2					33.51	2	1	2
Q9UQ07	MOK	MOK MAPK/MAK/MRK overlapping kinase Tax_Id=9606	48013	Homo sapiens	59.39	3	1	2	63.92	3	1	1	26.09	3	1	1
Q5VUB5	FAM171A1	FAM171A1 Protein FAM171A1 Tax_Id=9606	97854	Homo sapiens	59.33	1	1	1					33.75	1	1	1
Q14055	COL9A2	COL9A2 Collagen alpha-2(IX) chain Tax_Id=9606	65131	Homo sapiens	59.33	3	1	1					35.76	3	1	1
Q92959	SLCO2A1	SLCO2A1 Solute carrier organic anion transporter family member 2A1 Tax_Id=9606	70044	Homo sapiens	58.97	3	1	1	60.63	3	1	1				
Q9H1M3	DEFB129	DEFB129 Beta-defensin 129 Tax_Id=9606	20298	Homo sapiens	58.93	11	1	1					33.63	11	1	1
P16662	UGT2B7	UGT2B7 UDP-glucuronosyltransferase 2B7 Tax_Id=9606	60694	Homo sapiens	58.87	4	1	1					37.24	4	1	1
O14531	DPYSL4	DPYSL4 Dihydropyrimidinase-related protein 4 Tax_Id=9606	61877	Homo sapiens	58.76	9	1	2					37.03	2	1	2
Q14994	NR1I3	NR1I3 Nuclear receptor subfamily 1 group I member 3 Tax_Id=9606	39942	Homo sapiens	58.53	6	1	1	61.56	3	1	1				
Q96K21	ZFYVE19	ZFYVE19 Zinc finger FYVE domain-containing protein 19 Tax_Id=9606	51546	Homo sapiens	58.49	6	1	2					36.21	6	1	1
Q5TZA2	CROCC	CROCC Rootletin Tax_Id=9606	228523	Homo sapiens	58.41	1	1	1					32.08	1	1	1

Q6UWY5	OLFML1	OLFML1 Olfactomedin-like protein 1 Tax_Id=9606	45951	Homo sapiens	58.34	3	1	1	56.52	3	1	1				
Q9UEY8	ADD3	ADD3 Gamma-adducin Tax_Id=9606	79154	Homo sapiens	58.31	4	1	1	61.23	3	1	1				
Q8NEZ4	KMT2C	KMT2C Histone-lysine N-methyltransferase 2C Tax_Id=9606	541371	Homo sapiens	58.03	2	1	1					34.33	1	1	1
Q96JM3	CHAMP1	CHAMP1 Chromosome alignment-maintaining phosphoprotein 1 Tax_Id=9606	89099	Homo sapiens	57.85	1	1	1	60.21	1	1	1				
O15131	KPNA5	KPNA5 Importin subunit alpha-6 Tax_Id=9606	60349	Homo sapiens	57.81	5	1	1	61.72	5	1	1				
P16152	CBR1	CBR1 Carbonyl reductase [NADPH] 1 Tax_Id=9606	30374	Homo sapiens	57.78	3	1	1	55.26	3	1	1				
Q9BPX7	C7orf25	C7orf25 UPF0415 protein C7orf25 Tax_Id=9606	46451	Homo sapiens	57.77	5	1	1	58.26	5	1	1				
P43487	RANBP1	RANBP1 Ran-specific GTPase-activating protein Tax_Id=9606	23310	Homo sapiens	57.22	18	1	1	58.95	18	1	1				
Q8WXX0	DNAH7	DNAH7 Dynein heavy chain 7, axonemal Tax_Id=9606	461160	Homo sapiens	57.14	2	1	5	51.66	2	1	3	36.07	1	1	2
Q8WXB4	ZNF606	ZNF606 Zinc finger protein 606 Tax_Id=9606	91812	Homo sapiens	56.71	5	1	1					33.41	2	1	1
Q9BZW8	CD244	CD244 Natural killer cell receptor 2B4 Tax_Id=9606	41616	Homo sapiens	56.68	2	1	1	53.52	2	1	1				
Q8TE58	ADAMTS15	ADAMTS15 A disintegrin and metalloproteinase with thrombospondin motifs 15 Tax_Id=	103287	Homo sapiens	56.62	7	1	1					34.07	6	1	1
Q8N4M1	SLC44A3	SLC44A3 Choline transporter-like protein 3 Tax_Id=9606	73783	Homo sapiens	56.37	4	1	1					33.61	4	1	1
Q6PRD1	GPR179	GPR179 Probable G-protein coupled receptor 179 Tax_Id=9606	257364	Homo sapiens	56.32	2	1	1	56.01	2	1	1				
P10768	ESD	ESD S-formylglutathione hydrolase Tax_Id=9606	31462	Homo sapiens	56.18	2	1	1					33.64	2	1	1
P30085	CMPK1	CMPK1 UMP-CMP kinase Tax_Id=9606	22222	Homo sapiens	56.02	4	1	1	54.11	4	1	1				
Q58FF6	HSP90AB4P	HSP90AB4P Putative heat shock protein HSP 90-beta 4 Tax_Id=9606	58264	Homo sapiens	55.66	3	1	2	54.56	3	1	1				
A0JNW5	UHRF1BP1L	UHRF1BP1L UHRF1-binding protein 1-like Tax_Id=9606	164198	Homo sapiens	55.14	3	1	1	57.48	3	1	1				
O00566	MPHOSPH10	MPHOSPH10 U3 small nucleolar ribonucleoprotein protein MPP10 Tax_Id=9606	78863	Homo sapiens	55.13	2	1	1	57.59	2	1	1				
O95995	GAS8	GAS8 Growth arrest-specific protein 8 Tax_Id=9606	56355	Homo sapiens	55.02	5	1	1					33.73	5	1	1
Q9BQ52	ELAC2	ELAC2 Zinc phosphodiesterase ELAC protein 2 Tax_Id=9606	92219	Homo sapiens	54.93	3	1	1					32.25	1	1	1
Q8IYJ2	C10orf67	C10orf67 Uncharacterized protein C10orf67 Tax_Id=9606	21594	Homo sapiens	54.69	6	1	1	53.79	6	1	1				
Q8TDW7	FAT3	FAT3 Protocadherin Fat 3 Tax_Id=9606	505524	Homo sapiens	54.52	1	1	1	53.61	1	1	1				
Q9Y223	GNE	GNE Bifunctional UDP-N-acetylglucosamine 2-epimerase/N-acetylmannosamine kinase T	79274	Homo sapiens	54.28	3	1	2					32.6	3	1	1
Q76N32	CEP68	CEP68 Centrosomal protein of 68 kDa Tax_Id=9606	81102	Homo sapiens	54.12	6	1	1	50.89	4	1	1				
Q3V6T2	CCDC88A	CCDC88A Girdin Tax_Id=9606	216041	Homo sapiens	53.94	3	1	1	51.24	2	1	1				
Q5QJ38	TCHHL1	TCHHL1 Trichohyalin-like protein 1 Tax_Id=9606	99275	Homo sapiens	53.77	4	1	1	48.55	2	1	1				
Q15008	PSMD6	PSMD6 26S proteasome non-ATPase regulatory subunit 6 Tax_Id=9606	45531	Homo sapiens	53.65	11	1	1					32.57	9	1	1
Q96S53	TESK2	TESK2 Dual specificity testis-specific protein kinase 2 Tax_Id=9606	63639	Homo sapiens	53.62	4	1	1	56.54	4	1	1				
Q8WU10	PYROXD1	PYROXD1 Pyridine nucleotide-disulfide oxidoreductase domain-containing protein 1 Tax_	55793	Homo sapiens	52.85	4	1	1	47.89	4	1	1				
Q92570	NR4A3	NR4A3 Nuclear receptor subfamily 4 group A member 3 Tax_Id=9606	68229	Homo sapiens	52.82	2	1	1	53.19	2	1	1				
Q86YB7	ECHDC2	ECHDC2 Enoyl-CoA hydratase domain-containing protein 2, mitochondrial Tax_Id=9606	31126	Homo sapiens	52.62	10	1	1	55.1	10	1	1				
Q99418	CYTH2	CYTH2 Cytohesin-2 Tax_Id=9606	46546	Homo sapiens	52.14	5	1	1	46.79	5	1	1				
Q9Y6N6	LAMC3	LAMC3 Laminin subunit gamma-3 Tax_Id=9606	171228	Homo sapiens	52.13	3	1	1	51.32	3	1	1				
Q5BJF6	ODF2	ODF2 Outer dense fiber protein 2 Tax_Id=9606	95401	Homo sapiens	51.79	8	1	1					34.1	5	1	1
O43150	ASAP2	ASAP2 Arf-GAP with SH3 domain, ANK repeat and PH domain-containing protein 2 Tax_I	111650	Homo sapiens	51.77	6	1	2	50.1	5	1	2				
Q8IWA4	MFN1	MFN1 Mitofusin-1 Tax_Id=9606	84100	Homo sapiens	51.75	3	1	2	27.61	3	1	1	26.73	3	1	1
Q5VT25	CDC42BPA	CDC42BPA Serine/threonine-protein kinase MRCK alpha Tax_Id=9606	197307	Homo sapiens	51.7	2	1	1	49.74	1	1	1				
Q6ZR08	DNAH12	DNAH12 Dynein heavy chain 12, axonemal Tax_Id=9606	356942	Homo sapiens	51.61	1	1	1					29.28	1	1	1
Q9BTP6	ZBED2	ZBED2 Zinc finger BED domain-containing protein 2 Tax_Id=9606	25122	Homo sapiens	51.32	15	1	1	48.36	15	1	2				
O43304	SEC14L5	SEC14L5 SEC14-like protein 5 Tax_Id=9606	78941	Homo sapiens	51.12	4	1	1					33.41	4	1	1
Q8NCY6	MSANTD4	MSANTD4 Myb/SANT-like DNA-binding domain-containing protein 4 Tax_Id=9606	41149	Homo sapiens	50.97	8	1	3	56.65	4	1	2	24.97	8	1	1
P35070	BTC	BTC Probetacellulin Tax_Id=9606	19746	Homo sapiens	50.95	6	1	1					27.41	6	1	1
P28331	NDUFS1	NDUFS1 NADH-ubiquinone oxidoreductase 75 kDa subunit, mitochondrial Tax_Id=9606	79467	Homo sapiens	50.86	4	1	1	52.06	4	1	1				
O15169	AXIN1	AXIN1 Axin-1 Tax_Id=9606	95635	Homo sapiens	50.43	1	1	1	45	1	1	1				
Q8WVS4	WDR60	WDR60 WD repeat-containing protein 60 Tax_Id=9606	122570	Homo sapiens	50.18	5	1	1	53.04	4	1	1				
Q8N228	SCML4	SCML4 Sex comb on midleg-like protein 4 Tax_Id=9606	45022	Homo sapiens	50.15	8	1	4	54.61	5	1	2				
Q5JV73	FRMPD3	FRMPD3 FERM and PDZ domain-containing protein 3 Tax_Id=9606	199209	Homo sapiens	50.07	2	1	1	51.5	2	1	1				
Q7L3T8	PARS2	PARS2 Probable proline--tRNA ligase, mitochondrial Tax_Id=9606	53262	Homo sapiens	49.84	4	1	2					34.72	3	1	2
Q9NZH5	PTTG2	PTTG2 Securin-2 Tax_Id=9606	22301	Homo sapiens	49.31	17	1	1					29.23	17	1	1
P24941	CDK2	CDK2 Cyclin-dependent kinase 2 Tax_Id=9606	33929	Homo sapiens	49.28	7	1	2					28.62	4	1	2
P21754-3	ZP3	ZP3 Isoform 3 of Zona pellucida sperm-binding protein 3 Tax_Id=9606	41271	Homo sapiens	49.23	3	1	1					26.44	3	1	1

Q8N9P6	C9orf163	C9orf163 Uncharacterized protein C9orf163 Tax_Id=9606	22153	Homo sapiens	48.63	5	1	4	26.83	5	1	1	29.05	5	1	2
Q58F21	BRDT	BRDT Bromodomain testis-specific protein Tax_Id=9606	107954	Homo sapiens	48.51	4	1	1	48.97	4	1	1				
Q6W2J9	BCOR	BCOR BCL-6 corepressor Tax_Id=9606	192189	Homo sapiens	48.46	6	1	3	29.1	5	1	2	28.71	4	1	1
Q17R55	FAM187B	FAM187B Protein FAM187B Tax_Id=9606	42386	Homo sapiens	48.28	5	1	1	47.42	5	1	1				
Q5T1A1	DCST2	DCST2 DC-STAMP domain-containing protein 2 Tax_Id=9606	86230	Homo sapiens	48.22	3	1	1	37.91	3	1	1				
P19801	AOC1	AOC1 Amiloride-sensitive amine oxidase [copper-containing] Tax_Id=9606	85377	Homo sapiens	48.01	2	1	1					25.74	2	1	1
Q8TBZ5	ZNF502	ZNF502 Zinc finger protein 502 Tax_Id=9606	62920	Homo sapiens	47.95	2	1	3	44.36	2	1	1	28.68	2	1	2
Q8N7U6	EFHB	EFHB EF-hand domain-containing family member B Tax_Id=9606	93802	Homo sapiens	47.75	4	1	1					27.94	4	1	1
O14607	UTY	UTY Histone demethylase UTY Tax_Id=9606	149548	Homo sapiens	47.64	1	1	1					27.48	1	1	1
Q99985	SEMA3C	SEMA3C Semaphorin-3C Tax_Id=9606	85207	Homo sapiens	47.28	1	1	1	47.91	1	1	1				
Q13347	EIF3I	EIF3I Eukaryotic translation initiation factor 3 subunit I Tax_Id=9606	36501	Homo sapiens	47.24	6	1	1	43.96	6	1	1				
Q5NE16	CTSL3P	CTSL3P Putative inactive cathepsin L-like protein CTSL3P Tax_Id=9606	25059	Homo sapiens	47.17	6	1	1	48.04	6	1	1				
O14920	IKBKB	IKBKB Inhibitor of nuclear factor kappa-B kinase subunit beta Tax_Id=9606	86564	Homo sapiens	47.1	6	1	1					30.33	2	1	1
P28062	PSMB8	PSMB8 Proteasome subunit beta type-8 Tax_Id=9606	30354	Homo sapiens	47.1	9	1	1	47.71	9	1	1				
Q8IYS0	GRAMD1C	GRAMD1C GRAM domain-containing protein 1C Tax_Id=9606	76035	Homo sapiens	47.08	5	1	1	44.53	4	1	1				
Q6ZT12	UBR3	UBR3 E3 ubiquitin-protein ligase UBR3 Tax_Id=9606	212434	Homo sapiens	46.93	3	1	1	37.76	2	1	1				
Q5JYT7	KIAA1755	KIAA1755 Uncharacterized protein KIAA1755 Tax_Id=9606	130846	Homo sapiens	46.68	3	1	1	47.04	3	1	1				
O14907	TAX1BP3	TAX1BP3 Tax1-binding protein 3 Tax_Id=9606	13734	Homo sapiens	46.64	15	1	1					26.25	15	1	1
Q8IWI9	MGA	MGA MAX gene-associated protein Tax_Id=9606	331837	Homo sapiens	46.22	2	1	1	48.03	2	1	1				
Q8NHM4	PRSS3P2	PRSS3P2 Putative trypsin-6 Tax_Id=9606	26537	Homo sapiens	45.71	17	1	1	46.1	17	1	1				
P63316	TNNC1	TNNC1 Troponin C, slow skeletal and cardiac muscles Tax_Id=9606	18402	Homo sapiens	45.6	27	1	1					31.1	27	1	1
O43861	ATP9B	ATP9B Probable phospholipid-transporting ATPase IIB Tax_Id=9606	129303	Homo sapiens	45.56	5	1	1					32.74	5	1	1
Q5W0V3-2	FAM160B1	FAM160B1 Isoform 2 of Protein FAM160B1 Tax_Id=9606	83735	Homo sapiens	45.49	5	1	2	39.63	5	1	1				
O60941	DTNB	DTNB Dystrobrevin beta Tax_Id=9606	71355	Homo sapiens	45.33	6	1	1					28.02	3	1	1
Q99829	CPNE1	CPNE1 Copine-1 Tax_Id=9606	59058	Homo sapiens	45.32	4	1	1	48.99	4	1	1				
O75815	BCAR3	BCAR3 Breast cancer anti-estrogen resistance protein 3 Tax_Id=9606	92565	Homo sapiens	45.3	5	1	1					28.57	2	1	1
P01871	IGHM	IGHM Ig mu chain C region Tax_Id=9606	49306	Homo sapiens	45.26	3	1	8	47.29	3	1	5				
Q5C9Z4	NOM1	NOM1 Nucleolar MIF4G domain-containing protein 1 Tax_Id=9606	96257	Homo sapiens	45.22	7	1	1	43.7	7	1	1				
Q14194-2	CRMP1	CRMP1 Isoform LCRMP-1 of Dihydropyrimidinase-related protein 1 Tax_Id=9606	74262	Homo sapiens	45.14	7	1	1	43.55	7	1	1				
Q6ZMP0	THSD4	THSD4 Thrombospondin type-1 domain-containing protein 4 Tax_Id=9606	112450	Homo sapiens	44.69	3	1	1					25.66	3	1	1
Q9HCS7	XAB2	XAB2 Pre-mRNA-splicing factor SYF1 Tax_Id=9606	100009	Homo sapiens	44.66	3	1	2	43.48	3	1	1	23.62	2	1	1
Q2KHR2	RFX7	RFX7 DNA-binding protein RFX7 Tax_Id=9606	146895	Homo sapiens	44.55	2	1	1	40.09	2	1	1				
Q63HN8	RNF213	RNF213 E3 ubiquitin-protein ligase RNF213 Tax_Id=9606	591409	Homo sapiens	43.95	1	2	2	46.31	1	2	2				
A1A4Y4	IRGM	IRGM Immunity-related GTPase family M protein Tax_Id=9606	20142	Homo sapiens	43.81	15	1	1	34.06	15	1	1				
Q14667	KIAA0100	KIAA0100 UPF0378 protein KIAA0100 Tax_Id=9606	253700	Homo sapiens	43.48	1	1	5	41.94	1	1	2				
P09497	CLTB	CLTB Clathrin light chain B Tax_Id=9606	25190	Homo sapiens	43.28	6	1	1					26.35	6	1	1
O14514	BAI1	BAI1 Brain-specific angiogenesis inhibitor 1 Tax_Id=9606	173501	Homo sapiens	43.06	1	1	1					24.99	1	1	1
Q96MF4	CCDC140	CCDC140 Coiled-coil domain-containing protein 140 Tax_Id=9606	18252	Homo sapiens	42.71	9	1	1	38.24	9	1	1				
Q9Y2G4	ANKRD6	ANKRD6 Ankyrin repeat domain-containing protein 6 Tax_Id=9606	79971	Homo sapiens	42.62	1	1	1					26.62	1	1	1
Q12830	BPTF	BPTF Nucleosome-remodeling factor subunit BPTF Tax_Id=9606	338263	Homo sapiens	42.55	2	1	4	25.76	1	1	1	24.72	2	1	2
Q9NZN3	EHD3	EHD3 EH domain-containing protein 3 Tax_Id=9606	60887	Homo sapiens	42.35	4	1	1	45.76	4	1	1				
Q9NPA5	ZFP64	ZFP64 Zinc finger protein 64 homolog, isoforms 1 and 2 Tax_Id=9606	74643	Homo sapiens	42.33	2	1	3	27.53	2	1	1	25.12	2	1	1
Q96BY6	DOCK10	DOCK10 Dedicator of cytokinesis protein 10 Tax_Id=9606	249531	Homo sapiens	42.29	2	1	1	44.95	1	1	1				
O94762	RECQL5	RECQL5 ATP-dependent DNA helicase Q5 Tax_Id=9606	108858	Homo sapiens	42.19	2	1	2	27.61	2	1	1	24.77	2	1	1
Q8IY21	DDX60	DDX60 Probable ATP-dependent RNA helicase DDX60 Tax_Id=9606	197853	Homo sapiens	41.93	3	2	2	38.81	2	2	2				
Q9UL68-3	MYT1L	MYT1L Isoform 3 of Myelin transcription factor 1-like protein Tax_Id=9606	20620	Homo sapiens	41.76	13	1	1	41.46	7	1	1				
P28482	MAPK1	MAPK1 Mitogen-activated protein kinase 1 Tax_Id=9606	41389	Homo sapiens	41.68	7	1	1					24.42	7	1	1
Q15935	ZNF77	ZNF77 Zinc finger protein 77 Tax_Id=9606	61964	Homo sapiens	41.64	7	1	2	40.05	7	1	2				
Q9UKK3	PARP4	PARP4 Poly [ADP-ribose] polymerase 4 Tax_Id=9606	192595	Homo sapiens	41.6	3	1	2	39.67	2	1	1				
Q8TB40	ABHD4	ABHD4 Abhydrolase domain-containing protein 4 Tax_Id=9606	38794	Homo sapiens	41.56	6	1	1					27.27	6	1	1
Q70JA7	CHSY3	CHSY3 Chondroitin sulfate synthase 3 Tax_Id=9606	100284	Homo sapiens	41.49	4	1	1					26.94	1	1	1
O14526	FCHO1	FCHO1 FCH domain only protein 1 Tax_Id=9606	96861	Homo sapiens	41.33	1	1	2					22.74	1	1	2

Q9Y421	FAM32A	FAM32A Protein FAM32A Tax_Id=9606	13178	Homo sapiens	41.3	24	1	1					25.33	24	1	1
Q6NY19	KANK3	KANK3 KN motif and ankyrin repeat domain-containing protein 3 Tax_Id=9606	88425	Homo sapiens	41.29	4	1	1	43	4	1	1				
Q0Z7S8	FABP9	FABP9 Fatty acid-binding protein 9 Tax_Id=9606	15092	Homo sapiens	41.16	11	1	2					26.29	11	1	2
Q8IZR5	CMTM4	CMTM4 CKLF-like MARVEL transmembrane domain-containing protein 4 Tax_Id=9606	25827	Homo sapiens	41.07	6	1	1	43.17	6	1	1				
P20591	MX1	MX1 Interferon-induced GTP-binding protein Mx1 Tax_Id=9606	75520	Homo sapiens	41.04	1	1	1	45.04	1	1	1				
Q5JU67	C9orf117	C9orf117 Uncharacterized protein C9orf117 Tax_Id=9606	60533	Homo sapiens	40.99	4	1	1					26.51	3	1	1
Q6PI48	DARS2	DARS2 Aspartate--tRNA ligase, mitochondrial Tax_Id=9606	73562	Homo sapiens	40.55	5	1	1	39.76	5	1	1				
Q8NFY9	KBTBD8	KBTBD8 Kelch repeat and BTB domain-containing protein 8 Tax_Id=9606	68822	Homo sapiens	40.5	2	1	1	39.18	2	1	1				
P29375	KDM5A	KDM5A Lysine-specific demethylase 5A Tax_Id=9606	192096	Homo sapiens	40.47	2	1	1					20.66	2	1	1
O75038-2	PLCH2	PLCH2 Isoform 2 of 1-phosphatidylinositol 4,5-bisphosphate phosphodiesterase eta-2 Ta	124302	Homo sapiens	40.4	3	1	1					21.31	3	1	1
Q9BSD7	NTPCR	NTPCR Cancer-related nucleoside-triphosphatase Tax_Id=9606	20713	Homo sapiens	39.88	6	1	1					27.37	6	1	1
Q8NCP5	ZBTB44	ZBTB44 Zinc finger and BTB domain-containing protein 44 Tax_Id=9606	63847	Homo sapiens	39.65	2	1	1	38.2	2	1	1				
Q9H9T3	ELP3	ELP3 Elongator complex protein 3 Tax_Id=9606	62259	Homo sapiens	39.61	3	1	1	37.62	3	1	1				
P40939	HADHA	HADHA Trifunctional enzyme subunit alpha, mitochondrial Tax_Id=9606	82999	Homo sapiens	39.52	4	1	1								
P11597	CETP	CETP Cholesteryl ester transfer protein Tax_Id=9606	54756	Homo sapiens	39.45	7	1	3	43.86	7	1	2				
A4FU01	MTMR11	MTMR11 Myotubularin-related protein 11 Tax_Id=9606	79545	Homo sapiens	39.43	1	1	3					23.55	1	1	1
Q9H1C3	GLT8D2	GLT8D2 Glycosyltransferase 8 domain-containing protein 2 Tax_Id=9606	40026	Homo sapiens	39.29	10	1	1	39.17	7	1	1				
P14735	IDE	IDE Insulin-degrading enzyme Tax_Id=9606	117968	Homo sapiens	39.26	3	1	1	35.93	1	1	1				
Q8NG08	HELB	HELB DNA helicase B Tax_Id=9606	123252	Homo sapiens	39.14	3	1	5	45.49	2	1	4				
Q9NVR7	TBCCD1	TBCCD1 TBCC domain-containing protein 1 Tax_Id=9606	63530	Homo sapiens	39	4	1	1					22.82	4	1	1
Q1RMZ1	C7orf60	C7orf60 Probable methyltransferase BTM2 homolog Tax_Id=9606	46323	Homo sapiens	38.87	3	1	1	30.63	3	1	1				
Q9P212	PLCE1	PLCE1 1-phosphatidylinositol 4,5-bisphosphate phosphodiesterase epsilon-1 Tax_Id=960	258715	Homo sapiens	38.83	2	1	1					25.65	2	1	1
Q02388	COL7A1	COL7A1 Collagen alpha-1(VII) chain Tax_Id=9606	295219	Homo sapiens	38.8	0	1	1					23.24	0	1	1
Q14691	GINS1	GINS1 DNA replication complex GINS protein PSF1 Tax_Id=9606	22988	Homo sapiens	38.8	4	1	1	33.74	4	1	1				
Q08629	SPOCK1	SPOCK1 Testican-1 Tax_Id=9606	49124	Homo sapiens	38.52	9	1	1					23.39	5	1	1
O75792	RNASEH2A	RNASEH2A Ribonuclease H2 subunit A Tax_Id=9606	33394	Homo sapiens	38.37	10	1	1	41.84	10	1	1				
Q09328	MGAT5	MGAT5 Alpha-1,6-mannosylglycoprotein 6-beta-N-acetylglucosaminyltransferase A Tax_	84542	Homo sapiens	38.17	3	1	1	38.3	3	1	1				
Q9NRY4	ARHGAP35	ARHGAP35 Rho GTPase-activating protein 35 Tax_Id=9606	170514	Homo sapiens	37.85	1	1	1					21.65	1	1	1
P49588	AARS	AARS Alanine--tRNA ligase, cytoplasmic Tax_Id=9606	106810	Homo sapiens	37.78	5	1	1	39.53	4	1	1				
Q9H4I3	TRABD	TRABD TraB domain-containing protein Tax_Id=9606	42321	Homo sapiens	37.28	7	1	1					24.21	7	1	1
Q9H074	PAIP1	PAIP1 Polyadenylate-binding protein-interacting protein 1 Tax_Id=9606	53524	Homo sapiens	37.17	3	1	1	36.28	3	1	1				
Q9P2E5	CHPF2	CHPF2 Chondroitin sulfate glucuronyltransferase Tax_Id=9606	85948	Homo sapiens	37.12	2	1	1	40.15	2	1	1				
Q96S86	HAPLN3	HAPLN3 Hyaluronan and proteoglycan link protein 3 Tax_Id=9606	40894	Homo sapiens	37.03	4	1	2	34.28	4	1	2				
Q8IWB9	TEX2	TEX2 Testis-expressed sequence 2 protein Tax_Id=9606	125302	Homo sapiens	36.98	3	2	2	41.06	3	2	2				
Q9Y603	ETV7	ETV7 Transcription factor ETV7 Tax_Id=9606	38997	Homo sapiens	36.95	4	1	1								
Q15428	SF3A2	SF3A2 Splicing factor 3A subunit 2 Tax_Id=9606	49255	Homo sapiens	36.76	3	1	1					21.33	3	1	1
P20248	CCNA2	CCNA2 Cyclin-A2 Tax_Id=9606	48550	Homo sapiens	36.66	11	1	1					20.69	8	1	1
Q9H4Z3	PCIF1	PCIF1 Phosphorylated CTD-interacting factor 1 Tax_Id=9606	80669	Homo sapiens	36.62	3	1	1	39.02	3	1	1				
P18031	PTPN1	PTPN1 Tyrosine-protein phosphatase non-receptor type 1 Tax_Id=9606	49966	Homo sapiens	36.61	6	1	1					20.58	3	1	1
O95822	MLYCD	MLYCD Malonyl-CoA decarboxylase, mitochondrial Tax_Id=9606	55003	Homo sapiens	36.47	5	1	1	37.96	5	1	1				
P46063	RECQL	RECQL ATP-dependent DNA helicase Q1 Tax_Id=9606	73457	Homo sapiens	36.2	3	1	1	30.01	3	1	1				
A6NGG8	C2orf71	C2orf71 Uncharacterized protein C2orf71 Tax_Id=9606	139655	Homo sapiens	36.11	6	1	1								
P08174-6	CD55	CD55 Isoform 6 of Complement decay-accelerating factor Tax_Id=9606	56217	Homo sapiens	35.96	6	1	1	29.46	6	1	1				
Q6P4A8	PLBD1	PLBD1 Phospholipase B-like 1 Tax_Id=9606	63255	Homo sapiens	35.95	3	1	1					23.07	3	1	1
Q9ULX7	CA14	CA14 Carbonic anhydrase 14 Tax_Id=9606	37667	Homo sapiens	35.51	6	1	1	36.17	4	1	1				
Q9Y2D0	CA5B	CA5B Carbonic anhydrase 5B, mitochondrial Tax_Id=9606	36433	Homo sapiens	35.47	9	1	1					21.49	6	1	1
Q9Y696	CLIC4	CLIC4 Chloride intracellular channel protein 4 Tax_Id=9606	28772	Homo sapiens	35.37	9	1	1	35.58	9	1	1				
Q08499-6	PDE4D	PDE4D Isoform 5 of cAMP-specific 3',5'-cyclic phosphodiesterase 4D Tax_Id=9606	84427	Homo sapiens	35.35	3	1	2	32.2	1	1	1				
O94779	CNTN5	CNTN5 Contactin-5 Tax_Id=9606	120686	Homo sapiens	35.31	3	1	1					23.68	3	1	1
O60264	SMARCA5	SMARCA5 SWI/SNF-related matrix-associated actin-dependent regulator of chromatin su	121905	Homo sapiens	35.29	3	1	1					22.8	3	1	1
Q8TCU5	GRIN3A	GRIN3A Glutamate receptor ionotropic, NMDA 3A Tax_Id=9606	125465	Homo sapiens	35.22	2	1	1	33.48	2	1	1				
Q15399	TLR1	TLR1 Toll-like receptor 1 Tax_Id=9606	90291	Homo sapiens	35.21	4	1	1	37.25	4	1	1				

Q9H3R0	KDM4C	KDM4C Lysine-specific demethylase 4C Tax_Id=9606	119982	Homo sapiens	35.07	1	1	1	35.99	1	1	1				
P14410		SI Sucrase-isomaltase, intestinal Tax_Id=9606	209453	Homo sapiens	35.01	4	1	1	35.09	4	1	1				
Q16740	CLPP	CLPP ATP-dependent Clp protease proteolytic subunit, mitochondrial Tax_Id=9606	30180	Homo sapiens	35.01	5	1	1					20.64	5	1	1
P13727	PRG2	PRG2 Bone marrow proteoglycan Tax_Id=9606	25205	Homo sapiens	34.96	11	1	1					21.19	11	1	1
Q9Y5B9	SUPT16H	SUPT16H FACT complex subunit SPT16 Tax_Id=9606	119914	Homo sapiens	34.72	6	1	1	33.1	6	1	1				
Q5TC82	RC3H1	RC3H1 Roquin-1 Tax_Id=9606	125736	Homo sapiens	34.71	4	1	1								
Q96MD7	C9orf85	C9orf85 Uncharacterized protein C9orf85 Tax_Id=9606	20165	Homo sapiens	34.58	22	1	1	35.96	18	1	1				
Q5T2W1	PDZK1	PDZK1 Na(+)/H(+) exchange regulatory cofactor NHE-RF3 Tax_Id=9606	57129	Homo sapiens	34.39	5	1	1	38.46	5	1	1				
Q92546	RGP1	RGP1 Retrograde Golgi transport protein RGP1 homolog Tax_Id=9606	42455	Homo sapiens	34.16	5	1	1								
Q9P107	GMIP	GMIP GEM-interacting protein Tax_Id=9606	106683	Homo sapiens	34.02	2	1	1	37.74	2	1	1				
Q9UHY7	ENOPH1	ENOPH1 Enolase-phosphatase E1 Tax_Id=9606	28932	Homo sapiens	33.97	17	1	1	38.11	17	1	1				
P52737	ZNF136	ZNF136 Zinc finger protein 136 Tax_Id=9606	62783	Homo sapiens	33.92	4	1	1					22.52	4	1	1
Q96T23	RSF1	RSF1 Remodeling and spacing factor 1 Tax_Id=9606	163820	Homo sapiens	33.74	2	1	1					21.66	2	1	1
Q14152	EIF3A	EIF3A Eukaryotic translation initiation factor 3 subunit A Tax_Id=9606	166569	Homo sapiens	33.69	2	1	1	36.09	2	1	1				
P07196	NEFL	NEFL Neurofilament light polypeptide Tax_Id=9606	61516	Homo sapiens	33.63	12	1	1	35.89	12	1	1				
A2VEC9-2	SSPO	SSPO Isoform 2 of SCO-spondin Tax_Id=9606	138775	Homo sapiens	33.54	2	1	1					20.44	2	1	1
A6PVC2	TTLL8	TTLL8 Protein monoglycylase TTLL8 Tax_Id=9606	94676	Homo sapiens	33.54	5	1	4	35.34	2	1	3	23.17	5	1	1
Q9BXT5	TEX15	TEX15 Testis-expressed sequence 15 protein Tax_Id=9606	315337	Homo sapiens	33.54	3	1	1	33.94	2	1	1				
Q96MG2	JSRP1	JSRP1 Junctional sarcoplasmic reticulum protein 1 Tax_Id=9606	36318	Homo sapiens	33.41	6	1	1	34.11	6	1	1				
Q9UQ13	SHOC2	SHOC2 Leucine-rich repeat protein SHOC-2 Tax_Id=9606	64888	Homo sapiens	33.33	2	1	1								
Q96LB8	PGLYRP4	PGLYRP4 Peptidoglycan recognition protein 4 Tax_Id=9606	40620	Homo sapiens	33.2	9	1	1	36.79	5	1	1				
Q07283	TCHH	TCHH Trichohyalin Tax_Id=9606	253925	Homo sapiens	33.05	4	1	1					22.04	2	1	1
Q9NRX4-2	PHPT1	PHPT1 Isoform 2 of 14 kDa phosphohistidine phosphatase Tax_Id=9606	13671	Homo sapiens	32.89	25	1	1	35.22	15	1	1				
O95425	SVIL	SVIL Supervillin Tax_Id=9606	247746	Homo sapiens	32.43	3	2	5	32.68	2	1	1				
Q96P50	ACAP3	ACAP3 Arf-GAP with coiled-coil, ANK repeat and PH domain-containing protein 3 Tax_Id=	92494	Homo sapiens	32.25	3	1	1	34.87	3	1	1				
P52756	RBM5	RBM5 RNA-binding protein 5 Tax_Id=9606	92154	Homo sapiens	32.22	5	1	1					21.69	3	1	1
O60333	KIF1B	KIF1B Kinesin-like protein KIF1B Tax_Id=9606	204476	Homo sapiens	31.85	2	1	1	35.42	2	1	1				
Q9P0K9	FRRS1L	FRRS1L DOMON domain-containing protein FRRS1L Tax_Id=9606	37270	Homo sapiens	31.82	11	1	1					21.62	11	1	1
Q13976	PRKG1	PRKG1 cGMP-dependent protein kinase 1 Tax_Id=9606	76364	Homo sapiens	31.74	4	1	1	28.38	4	1	1				
Q00G26	PLIN5	PLIN5 Perilipin-5 Tax_Id=9606	50791	Homo sapiens	31.68	5	1	3								
P28335	HTR2C	HTR2C 5-hydroxytryptamine receptor 2C Tax_Id=9606	51821	Homo sapiens	31.53	8	1	1	35.49	8	1	1				
O95235	KIF20A	KIF20A Kinesin-like protein KIF20A Tax_Id=9606	100278	Homo sapiens	31.26	3	1	2					20.16	3	1	1
P32929-3	CTH	CTH Isoform 3 of Cystathionine gamma-lyase Tax_Id=9606	41260	Homo sapiens	31.25	7	1	2	31.54	7	1	1				
Q8N7E2	ZNF645	ZNF645 E3 ubiquitin-protein ligase ZNF645 Tax_Id=9606	48784	Homo sapiens	31.19	4	1	2	25.95	4	1	1				
P55017	SLC12A3	SLC12A3 Solute carrier family 12 member 3 Tax_Id=9606	113139	Homo sapiens	31.02	1	1	1	29.88	1	1	1				
Q9Y6Y1-4	CAMTA1	CAMTA1 Isoform 4 of Calmodulin-binding transcription activator 1 Tax_Id=9606	11480	Homo sapiens	30.97	19	1	1	29.03	19	1	1				
P40617	ARL4A	ARL4A ADP-ribosylation factor-like protein 4A Tax_Id=9606	22615	Homo sapiens	30.76	10	1	1	29.63	10	1	1				
Q6UXS9-2	CASP12	CASP12 Isoform 2 of Inactive caspase-12 Tax_Id=9606	19124	Homo sapiens	30.62	6	1	1					20.34	6	1	1
P35348-4	ADRA1A	ADRA1A Isoform 4 of Alpha-1A adrenergic receptor Tax_Id=9606	50777	Homo sapiens	30.46	2	1	1	28.32	2	1	1				
Q96JG6	CCDC132	CCDC132 Coiled-coil domain-containing protein 132 Tax_Id=9606	111174	Homo sapiens	30.2	3	1	1								
Q92736	RYR2	RYR2 Ryanodine receptor 2 Tax_Id=9606	564569	Homo sapiens	30.18	2	1	1	29.06	1	1	1				
Q9Y5H3	PCDHGA10	PCDHGA10 Protocadherin gamma-A10 Tax_Id=9606	101446	Homo sapiens	30.14	1	1	1					20.1	1	1	1
Q99496	RNF2	RNF2 E3 ubiquitin-protein ligase RING2 Tax_Id=9606	37655	Homo sapiens	30.05	5	1	1					21.15	3	1	1
P02766	TTR	TTR Transthyretin Tax_Id=9606	15887	Homo sapiens	30.03	10	1	1	32.56	10	1	1				
O15021	MAST4	MAST4 Microtubule-associated serine/threonine-protein kinase 4 Tax_Id=9606	284378	Homo sapiens	29.98	3	1	1	28.78	3	1	1				
P18054	ALOX12	ALOX12 Arachidonate 12-lipoxygenase, 12S-type Tax_Id=9606	75694	Homo sapiens	29.91	5	1	1	31.33	2	1	1				
Q5TCZ1	SH3PXD2A	SH3PXD2A SH3 and PX domain-containing protein 2A Tax_Id=9606	125289	Homo sapiens	29.91	5	1	1					24.51	5	1	1
P56715	RP1	RP1 Oxygen-regulated protein 1 Tax_Id=9606	240661	Homo sapiens	29.9	1	1	2	29.63	1	1	1				
Q7L9B9	EEPD1	EEPD1 Endonuclease/exonuclease/phosphatase family domain-containing protein 1 Tax_	62402	Homo sapiens	29.87	6	1	1	31.31	6	1	1				
P01023	A2M	A2M Alpha-2-macroglobulin Tax_Id=9606	163291	Homo sapiens	29.83	2	1	1	29.47	2	1	1				
Q13107	USP4	USP4 Ubiquitin carboxyl-terminal hydrolase 4 Tax_Id=9606	108565	Homo sapiens	29.8	4	1	1								
Q8WZA9	IRGQ	IRGQ Immunity-related GTPase family Q protein Tax_Id=9606	62717	Homo sapiens	29.68	11	1	1	34.92	11	1	1				

Q9Y4B6	VPRBP	VPRBP Protein VPRBP Tax_Id=9606	169007	Homo sapiens	29.59	2	1	1								
Q13085	ACACA	ACACA Acetyl-CoA carboxylase 1 Tax_Id=9606	265554	Homo sapiens	29.58	2	1	1					21.13	1	1	1
Q9NS56	TOPORS	TOPORS E3 ubiquitin-protein ligase Topors Tax_Id=9606	119197	Homo sapiens	29.57	2	1	1	33.37	2	1	1				
P50851	LRBA	LRBA Lipopolysaccharide-responsive and beige-like anchor protein Tax_Id=9606	319108	Homo sapiens	29.54	2	1	1	28.12	2	1	1				
Q8N4L4	SPEM1	SPEM1 Spermatid maturation protein 1 Tax_Id=9606	34773	Homo sapiens	29.24	3	1	1	32.7	3	1	1				
Q9H596	DUSP21	DUSP21 Dual specificity protein phosphatase 21 Tax_Id=9606	21528	Homo sapiens	29.2	9	1	1								
P01621		Ig kappa chain V-III region NG9 (Fragment) Tax_Id=9606	10728	Homo sapiens	29.19	9	1	1	26.05	9	1	1				
Q00688	FKBP3	FKBP3 Peptidyl-prolyl cis-trans isomerase FKBP3 Tax_Id=9606	25176	Homo sapiens	29.13	6	1	1	29.48	6	1	1				
P0C671	C6orf222	C6orf222 Uncharacterized protein C6orf222 Tax_Id=9606	71930	Homo sapiens	29.04	2	1	1	25.96	2	1	1				
O15090	ZNF536	ZNF536 Zinc finger protein 536 Tax_Id=9606	141417	Homo sapiens	29	2	1	1	27.44	2	1	1				
Q9Y592	CEP83	CCDC41 Centrosomal protein of 83 kDa Tax_Id=9606	82059	Homo sapiens	28.92	8	1	1								
Q14320	FAM50A	FAM50A Protein FAM50A Tax_Id=9606	40241	Homo sapiens	28.78	7	1	1	31.89	7	1	1				
Q9NVI7-2	ATAD3A	ATAD3A Isoform 2 of ATPase family AAA domain-containing protein 3A Tax_Id=9606	66218	Homo sapiens	28.78	12	1	1	31.51	9	1	1				
Q00536	CDK16	CDK16 Cyclin-dependent kinase 16 Tax_Id=9606	55715	Homo sapiens	28.62	5	1	1	26.54	5	1	1				
P07357	C8A	C8A Complement component C8 alpha chain Tax_Id=9606	65163	Homo sapiens	28.14	5	1	1					21.13	5	1	1
Q9NXX6	NSMCE4A	NSMCE4A Non-structural maintenance of chromosomes element 4 homolog A Tax_Id=96	44301	Homo sapiens	28.12	12	1	1								
Q13423	NNT	NNT NAD(P) transhydrogenase, mitochondrial Tax_Id=9606	113895	Homo sapiens	28.03	3	1	1								
Q1L5Z9	LONRF2	LONRF2 LON peptidase N-terminal domain and RING finger protein 2 Tax_Id=9606	83654	Homo sapiens	28	4	1	1	29.44	4	1	1				
Q8NHR7	C15orf43	C15orf43 Uncharacterized protein C15orf43 Tax_Id=9606	25315	Homo sapiens	28	6	1	1								
Q9HCR9	PDE11A	PDE11A Dual 3',5'-cyclic-AMP and -GMP phosphodiesterase 11A Tax_Id=9606	104751	Homo sapiens	27.99	4	1	1								
Q96S16	JMJD8	JMJD8 JmjC domain-containing protein 8 Tax_Id=9606	36869	Homo sapiens	27.95	8	1	1	29.48	8	1	1				
Q9NP80	PNPLA8	PNPLA8 Calcium-independent phospholipase A2-gamma Tax_Id=9606	88477	Homo sapiens	27.89	5	1	1	33.23	5	1	1				
Q6P2C0	WDR93	WDR93 WD repeat-containing protein 93 Tax_Id=9606	77378	Homo sapiens	27.85	4	1	1								
Q96T76	MMS19	MMS19 MMS19 nucleotide excision repair protein homolog Tax_Id=9606	113289	Homo sapiens	27.74	3	1	2	28.14	3	1	2				
Q9ULS6	KCNS2	KCNS2 Potassium voltage-gated channel subfamily S member 2 Tax_Id=9606	54237	Homo sapiens	27.73	6	1	1	29.4	6	1	1				
Q8WY41	NANOS1	NANOS1 Nanos homolog 1 Tax_Id=9606	30230	Homo sapiens	27.66	16	1	1	26.54	9	1	1				
Q8N8E3	CEP112	CEP112 Centrosomal protein of 112 kDa Tax_Id=9606	112748	Homo sapiens	27.58	1	1	1	25.38	1	1	1				
Q9NX61	TMEM161A	TMEM161A Transmembrane protein 161A Tax_Id=9606	53602	Homo sapiens	27.24	2	1	1	28.28	2	1	1				
Q9HBJ0	PLAC1	PLAC1 Placenta-specific protein 1 Tax_Id=9606	23616	Homo sapiens	27.22	8	1	1	29.65	8	1	1				
Q9UL45	BLOC1S6	BLOC1S6 Biogenesis of lysosome-related organelles complex 1 subunit 6 Tax_Id=9606	19743	Homo sapiens	26.96	9	1	1	26.17	9	1	1				
Q8WXI7	MUC16	MUC16 Mucin-16 Tax_Id=9606	2353227	Homo sapiens	26.79	0	1	1								
Q8IVG5	SAMD9L	SAMD9L Sterile alpha motif domain-containing protein 9-like Tax_Id=9606	184533	Homo sapiens	26.71	2	1	1	25.28	2	1	1				
O00418	EEF2K	EEF2K Eukaryotic elongation factor 2 kinase Tax_Id=9606	82144	Homo sapiens	26.54	2	1	1	26.99	2	1	1				
O43488	AKR7A2	AKR7A2 Aflatoxin B1 aldehyde reductase member 2 Tax_Id=9606	39589	Homo sapiens	26.42	8	1	1								
Q5SZK8	FREM2	FREM2 FRAS1-related extracellular matrix protein 2 Tax_Id=9606	351157	Homo sapiens	26.33	1	1	1	26.77	1	1	1				
Q8WUA4	GTF3C2	GTF3C2 General transcription factor 3C polypeptide 2 Tax_Id=9606	100679	Homo sapiens	26.23	2	1	1	24.27	2	1	1				
O60427	FADS1	FADS1 Fatty acid desaturase 1 Tax_Id=9606	51964	Homo sapiens	26.05	8	1	1								
Q12929	EPS8	EPS8 Epidermal growth factor receptor kinase substrate 8 Tax_Id=9606	91881	Homo sapiens	25.99	4	1	1								
O75880	SCO1	SCO1 Protein SCO1 homolog, mitochondrial Tax_Id=9606	33814	Homo sapiens	25.89	7	1	1								
Q9BY11	PACSIN1	PACSIN1 Protein kinase C and casein kinase substrate in neurons protein 1 Tax_Id=9606	50965	Homo sapiens	25.85	2	1	1	27.33	2	1	1				
Q96AC6	KIFC2	KIFC2 Kinesin-like protein KIFC2 Tax_Id=9606	90147	Homo sapiens	25.53	1	1	1	25.14	1	1	1				
Q0VF96	CGNL1	CGNL1 Cingulin-like protein 1 Tax_Id=9606	149079	Homo sapiens	25.45	2	1	1								
Q2LD37	KIAA1109	KIAA1109 Uncharacterized protein KIAA1109 Tax_Id=9606	555483	Homo sapiens	25.31	1	1	1	30.02	1	1	1				
Q8IX12	CCAR1	CCAR1 Cell division cycle and apoptosis regulator protein 1 Tax_Id=9606	132821	Homo sapiens	25.23	1	1	1	27.02	1	1	1				
Q96Q42	ALS2	ALS2 Alsin Tax_Id=9606	183633	Homo sapiens	24.52	1	1	1	28.36	1	1	1				
Q9ULD9	ZNF608	ZNF608 Zinc finger protein 608 Tax_Id=9606	162208	Homo sapiens	24.4	3	1	1								
O15083	ERC2	ERC2 ERC protein 2 Tax_Id=9606	110557	Homo sapiens	24.34	5	1	1								
P12814	ACTN1	ACTN1 Alpha-actinin-1 Tax_Id=9606	103057	Homo sapiens	24.16	3	1	1								
Q9UMN6	KMT2B	KMT2B Histone-lysine N-methyltransferase 2B Tax_Id=9606	293515	Homo sapiens	24.06	3	1	1	25.37	3	1	1				
Q96HD1	CRELD1	CRELD1 Cysteine-rich with EGF-like domain protein 1 Tax_Id=9606	45439	Homo sapiens	24.05	3	1	1	26.26	3	1	1				
Q9UNA4	POLI	POLI DNA polymerase iota Tax_Id=9606	83006	Homo sapiens	23.99	6	1	2	24.2	6	1	1				
Q92766-6	RREB1	RREB1 Isoform 6 of Ras-responsive element-binding protein 1 Tax_Id=9606	34614	Homo sapiens	23.93	6	1	1	25	6	1	1				

Q9Y6R7	FCGBP	FCGBP IgGFc-binding protein Tax_Id=9606	572019	Homo sapiens	23.93	1	1	1	25.71	1	1	1
Q9HBG7	LY9	LY9 T-lymphocyte surface antigen Ly-9 Tax_Id=9606	72139	Homo sapiens	23.89	4	1	1	21.53	4	1	1
Q8IXJ9	ASXL1	ASXL1 Putative Polycomb group protein ASXL1 Tax_Id=9606	165448	Homo sapiens	23.84	1	1	1				
Q6XPS3	TPTE2	TPTE2 Phosphatidylinositol 3,4,5-trisphosphate 3-phosphatase TPTE2 Tax_Id=9606	61111	Homo sapiens	23.64	8	1	1	25	8	1	1
O94851	MICAL2	MICAL2 Protein-methionine sulfoxide oxidase MICAL2 Tax_Id=9606	126689	Homo sapiens	23.6	5	1	1	25.94	5	1	1
P51854	TKTL1	TKTL1 Transketolase-like protein 1 Tax_Id=9606	65333	Homo sapiens	23.59	5	1	1				
Q14515	SPARCL1	SPARCL1 SPARC-like protein 1 Tax_Id=9606	75207	Homo sapiens	23.52	6	1	1				
O95239	KIF4A	KIF4A Chromosome-associated kinesin KIF4A Tax_Id=9606	139881	Homo sapiens	23.3	3	1	1				
Q9UK61	FAM208A	FAM208A Protein FAM208A Tax_Id=9606	189031	Homo sapiens	22.97	3	1	1	25.81	3	1	1
P11532	DMD	DMD Dystrophin Tax_Id=9606	426750	Homo sapiens	22.74	1	1	1				
Q8N5C6	SRBD1	SRBD1 S1 RNA-binding domain-containing protein 1 Tax_Id=9606	111775	Homo sapiens	22.7	1	1	1	27.01	1	1	1
Q07973	CYP24A1	CYP24A1 1,25-dihydroxyvitamin D(3) 24-hydroxylase, mitochondrial Tax_Id=9606	58875	Homo sapiens	22.47	7	1	1				
Q9ULI3	HEG1	HEG1 Protein HEG homolog 1 Tax_Id=9606	147460	Homo sapiens	22.28	4	1	1				
O14544	SOCS6	SOCS6 Suppressor of cytokine signaling 6 Tax_Id=9606	59528	Homo sapiens	21.95	1	1	1				
Q9UGL1	KDM5B	KDM5B Lysine-specific demethylase 5B Tax_Id=9606	175658	Homo sapiens	21.95	3	1	1	23.79	3	1	1
Q8NFZ3	NLGN4Y	NLGN4Y Neuroligin-4, Y-linked Tax_Id=9606	92020	Homo sapiens	21.9	7	1	1	21.12	7	1	1
O60245	PCDH7	PCDH7 Protocadherin-7 Tax_Id=9606	116071	Homo sapiens	21.87	3	1	1	22.48	3	1	1
Q86UW9	DTX2	DTX2 Probable E3 ubiquitin-protein ligase DTX2 Tax_Id=9606	67246	Homo sapiens	21.86	2	1	1				
Q96RS0	TGS1	TGS1 Trimethylguanosine synthase Tax_Id=9606	96620	Homo sapiens	21.84	3	1	1				
Q5HYC2	KIAA2026	KIAA2026 Uncharacterized protein KIAA2026 Tax_Id=9606	228087	Homo sapiens	21.76	3	1	1	25.91	1	1	1
Q96M66		Putative uncharacterized protein FLJ32790 Tax_Id=9606	20693	Homo sapiens	21.74	5	1	1	23.07	5	1	1
Q9NQH7	XPNPEP3	XPNPEP3 Probable Xaa-Pro aminopeptidase 3 Tax_Id=9606	57033	Homo sapiens	21.6	3	1	1				
Q8NFY4	SEMA6D	SEMA6D Semaphorin-6D Tax_Id=9606	119872	Homo sapiens	21.38	8	1	1	20.94	5	1	1

UniProt Accession, Gene Name, Protein Description, Theoretical Molecular Weight (CalcMr), Species, [a]Protein Score, [b]Percent Sequence Coverage, [c]Number of Significant Peptide Matches, [d]Number of Spectral Counts.

TABLE S1B: Proteomics Analysis of Plasmodium EV Fractions - Plasmodium					Combined Analysis				Biological Replicate 1				Biological Replicate 2			
Accession	Genename	Description	CalcMr	Species	Score[a]	Cov(%)[b]	SigM[c]	SpC[d]	Score[a]	Cov(%)[b]	SigM[c]	SpC[d]	Score[a]	Cov(%)[b]	SigM[c]	SpC[d]
Q8IJN7	ENO	ENO Enolase Tax_Id=36329	48677	Plasmodium falciparum	5871.52	88	35	111	5372.69	86	34	79	2993.29	58	20	31
Q7KQL9		PF14_0425 Fructose-bisphosphate aldolase Tax_Id=36329	40105	Plasmodium falciparum	5325.62	78	31	65	5144.26	78	30	49	1186.14	26	10	16
Q9TY95	SERA	SERA Serine-repeat antigen protein Tax_Id=36329	111768	Plasmodium falciparum	5286.77	47	42	98	5190.19	45	42	84	907.64	15	10	14
Q8I3X4		PFE0660c Purine nucleotide phosphorylase, putative Tax_Id=36329	26858	Plasmodium falciparum	3682.14	87	22	53	3617.58	87	22	32	1594.03	53	13	21
Q8IKK7	GAPDH	GAPDH Glyceraldehyde-3-phosphate dehydrogenase Tax_Id=36329	36635	Plasmodium falciparum	3270.79	81	21	67	3195.5	81	20	44	1125.09	46	11	23
P27362	PGK	PGK Phosphoglycerate kinase Tax_Id=36329	45426	Plasmodium falciparum	3069.24	67	24	73	3033.5	67	23	55	1300.15	40	11	18
Q8I0U8	MSP1	MSP1 Merozoite surface protein 1 Tax_Id=36329	195726	Plasmodium falciparum	2655.18	14	19	25	2328.38	11	15	20	368.27	4	5	5
Q8IEK1		MAL13P1.56 M1-family aminopeptidase Tax_Id=36329	126062	Plasmodium falciparum	2501.62	26	20	25	2461.13	26	20	20	290.76	8	4	5
C6KTA4		PFF1300w Pyruvate kinase Tax_Id=36329	55661	Plasmodium falciparum	2243.83	47	15	26	2236.91	47	15	25	154.26	6	1	1
Q8IJS1		PF10_0121 Hypoxanthine phosphoribosyltransferase Tax_Id=36329	26362	Plasmodium falciparum	2216.35	53	15	28	2205.95	53	15	20	635.19	26	5	8
Q8IB24		PF08_0054 Heat shock 70 kDa protein Tax_Id=36329	73915	Plasmodium falciparum	2087.51	32	18	48	2071.98	32	18	31	785.41	16	8	17
Q8I0P6		PF13_0304 Elongation factor 1-alpha Tax_Id=36329	48958	Plasmodium falciparum	1605.59	35	13	29	1515.53	35	12	24	304.68	16	5	5
Q7K6A4	PfSAMS	PfSAMS S-adenosylmethionine synthase Tax_Id=36329	44844	Plasmodium falciparum	1490.37	35	12	21	1462.74	32	12	16	359.07	16	4	5
Q8IKW5		PF14_0486 Elongation factor 2 Tax_Id=36329	93522	Plasmodium falciparum	1398.02	19	11	25	1337.84	19	10	13	705.32	10	7	12
Q8ILA4		PF14_0341 Glucose-6-phosphate isomerase Tax_Id=36329	67367	Plasmodium falciparum	1355.42	20	8	11	1352.43	18	8	11				
C0H5J9		MAL13P1.308 Uncharacterized protein Tax_Id=36329	288914	Plasmodium falciparum	1322.79	7	12	16	1308.12	7	12	15	47.53	1	1	1
Q76NL8	flN	flN Falcilysin Tax_Id=36329	138863	Plasmodium falciparum	1225.49	13	11	12	1214.25	11	10	10	157.07	4	2	2
K7NTP5		Hsp70-x Tax_Id=36329	75053	Plasmodium falciparum	1217.38	25	11	31	1206.22	25	11	20	413.65	12	4	11
Q8IIG6		PF11_0208 Phosphoglycerate mutase, putative Tax_Id=36329	28770	Plasmodium falciparum	1084.64	38	8	11	1083.83	38	8	11				
C6KT76		PFF1155w Hexokinase Tax_Id=36329	55261	Plasmodium falciparum	1062.99	32	8	8	1058.75	27	8	8				
Q8I566		PFL1720w Serine hydroxymethyltransferase Tax_Id=36329	49780	Plasmodium falciparum	1038.64	23	8	10	1043.05	23	8	8	80.08	7	2	2
Q7KQM0	TPI	TPI Triosephosphate isomerase Tax_Id=36329	27934	Plasmodium falciparum	1028.8	25	7	22	941.34	25	6	12	444.06	25	6	10
Q8I6U8	GBP	GBP Glycophorin-binding protein Tax_Id=36329	95844	Plasmodium falciparum	975.21	15	8	166	987.23	15	8	110	278.09	5	3	56
Q8IC05		PF07_0029 Heat shock protein 86 Tax_Id=36329	86166	Plasmodium falciparum	973.69	16	8	11	819.53	14	7	7	422.78	9	3	4
Q8I5D2	ABRA	ABRA 101 kDa malaria antigen Tax_Id=36329	86623	Plasmodium falciparum	961.02	14	7	13	962.12	14	7	12				
Q8I3M5		PFE1195w Karyopherin beta Tax_Id=36329	127353	Plasmodium falciparum	936	12	7	8	791.55	10	6	6	178.76	3	2	2
Q76NM6	vapA	vapA V-type proton ATPase catalytic subunit A Tax_Id=36329	68576	Plasmodium falciparum	867.05	18	7	8	865.47	18	7	7	28.67	3	1	1
P50250		PFE1050w Adenosylhomocysteinase Tax_Id=36329	53839	Plasmodium falciparum	859.08	21	9	11	864.85	21	9	10				
Q8IJA9		PF10_0289 Adenosine deaminase, putative Tax_Id=36329	42466	Plasmodium falciparum	826.59	22	6	13	827.53	22	6	11	86.01	4	2	2
Q8I3Y8		PFE0585c Myo-inositol 1-phosphate synthase, putative Tax_Id=36329	69112	Plasmodium falciparum	813.48	12	7	7	816.12	12	7	7				
Q8I603		PFL0210c Eukaryotic initiation factor 5a, putative Tax_Id=36329	17630	Plasmodium falciparum	764.11	39	5	13	767.67	39	5	12	28.89	7	1	1
Q6ZMA8		PFD0305c Vacuolar ATP synthase subunit b Tax_Id=36329	55788	Plasmodium falciparum	692.16	17	5	5	692.94	17	5	5				
Q8IDV0		PF13_0214 Elongation factor 1-gamma, putative Tax_Id=36329	47777	Plasmodium falciparum	692.16	18	5	5	692.94	18	5	5				
Q8IIK8	CyP22	CyP22 Peptidyl-prolyl cis-trans isomerase Tax_Id=36329	21731	Plasmodium falciparum	680.07	35	5	6	673.95	35	5	6				
Q8III5		PF11_0189 Insulinase, putative Tax_Id=36329	173625	Plasmodium falciparum	674.22	8	6	6	670.34	8	6	6				
Q8I5C5	MIF	MIF Macrophage migration inhibitory factor homologue Tax_Id=36329	12844	Plasmodium falciparum	668.27	41	5	16	624.84	41	5	9	421.09	36	4	7
Q8I1Y0	PF41	PF41 Merozoite surface protein P41 Tax_Id=36329	43088	Plasmodium falciparum	666.47	18	5	6	670.26	18	5	6				
Q8IL80	TPx1	TPx1 Thioredoxin peroxidase 1 Tax_Id=36329	21807	Plasmodium falciparum	613.83	36	6	11	603.44	36	6	7	270.87	18	3	4
Q8IC01		PF07_0033 Cg4 protein Tax_Id=36329	99964	Plasmodium falciparum	612.64	13	6	6	607.13	13	6	6				
Q8I4X0		PFL2215w Actin-1 Tax_Id=36329	41870	Plasmodium falciparum	590.49	18	5	10	589.15	18	5	9	154.26	11	1	1
Q8IL11	LAP	LAP M17 leucyl aminopeptidase Tax_Id=36329	67821	Plasmodium falciparum	557.73	10	4	4	558.35	10	4	4				
Q8ILT0		PF14_0164 Glutamate dehydrogenase Tax_Id=36329	52546	Plasmodium falciparum	531.79	16	4	4	522.13	16	4	4				
Q8I2X4		PFI0875w Heat shock protein 70 (HSP70) homologue Tax_Id=36329	72387	Plasmodium falciparum	530.98	9	4	6	423.77	9	3	4	207.28	5	2	2
C6KT34		PFF0940c Cell division cycle protein 48 homologue, putative Tax_Id=36329	92387	Plasmodium falciparum	527.43	10	4	5	423.77	8	3	3	66.65	1	1	2
O96164	SERA-4	SERA-4 Serine repeat antigen 4 (SERA-4) Tax_Id=36329	108681	Plasmodium falciparum	492.2	8	5	6	474.74	8	5	6				
Q8ID43		PF13_0349 Nucleoside diphosphate kinase Tax_Id=36329	17011	Plasmodium falciparum	475.44	31	4	6	461.89	31	4	4	144.62	19	1	1
Q8IDZ9		PF13_0179 Isoleucine-tRNA ligase, putative Tax_Id=36329	151311	Plasmodium falciparum	449.27	5	4	4	423.77	3	3	3	28.43	2	1	1
Q76NN7	PfCyP19	PfCyP19 Peptidyl-prolyl cis-trans isomerase Tax_Id=36329	18952	Plasmodium falciparum	441.87	42	4	9	438.8	42	4	7	137.15	12	2	2
C0H4Y6	PfPDI-8	PfPDI-8 Protein disulfide-isomerase Tax_Id=36329	55514	Plasmodium falciparum	425.15	8	4	4	421.19	6	4	4				

C6KT50		PFF1025c Pyridoxine/pyridoxal 5-phosphate biosynthesis enzyme Tax_Id=36329	33013	Plasmodium falciparum	423.3	20	3	4	423.77	20	3	3	142.71	4	1	1
Q8IDQ9	PfPMT	PfPMT Phosphoethanolamine N-methyltransferase Tax_Id=36329	31043	Plasmodium falciparum	423.3	13	3	4	423.77	13	3	4				
Q8IDJ8		PF13_0262 Lysine--tRNA ligase Tax_Id=36329	67590	Plasmodium falciparum	423.3	9	3	3	423.77	7	3	3				
Q8IAV1		PF08_0091 Uncharacterized protein Tax_Id=36329	144008	Plasmodium falciparum	422.96	7	4	6	393.22	7	4	4	25.05	2	1	2
Q8ILI6		PF14_0257 Acidic leucine-rich nuclear phosphoprotein 32-related protein Tax_Id=	33006	Plasmodium falciparum	413.99	13	3	5	415.4	13	3	5				
Q8IKT5		PF14_0517 Peptidase, putative Tax_Id=36329	88502	Plasmodium falciparum	404.04	7	5	5	396.3	7	5	5				
Q8I2J4	Pfn	Pfn Profilin Tax_Id=36329	19016	Plasmodium falciparum	394.24	19	3	7	383.24	19	3	7				
Q8IKC9		PF14_0676 Proteasome subunit beta type Tax_Id=36329	22862	Plasmodium falciparum	385.94	19	3	3	389.48	19	3	3				
Q7KQL8		PF14_0545 Thioredoxin Tax_Id=36329	11716	Plasmodium falciparum	332.4	42	4	5	329.68	42	4	5				
Q8I320	PfEF-1beta	PfEF-1beta Elongation factor 1-beta Tax_Id=36329	32026	Plasmodium falciparum	330.04	11	2	3	333.58	11	2	3				
Q8ILP3	Pf113	Pf113 Surface protein, Pf113 Tax_Id=36329	112574	Plasmodium falciparum	320.35	4	3	3	319.06	4	3	3				
Q8IJR9		PF10_0123 GMP synthetase Tax_Id=36329	63932	Plasmodium falciparum	316.78	12	3	3	306.71	11	3	3				
O15770	GR3	GR3 Glutathione reductase Tax_Id=36329	56492	Plasmodium falciparum	304.73	9	3	3	302.55	9	3	3				
C6KST3		PFF0420c Proteasome subunit alpha type 2, putative Tax_Id=36329	26528	Plasmodium falciparum	288.86	9	2	2	289.18	9	2	2				
Q8I261		PFA_0400c Beta3 proteasome subunit, putative Tax_Id=36329	23080	Plasmodium falciparum	288.86	13	2	2	289.18	13	2	2				
Q8I333		PFI0580c Falstatin, putative Tax_Id=36329	46958	Plasmodium falciparum	288.86	8	2	5	289.18	8	2	5				
Q8I3F1		PFE1600w Uncharacterized protein Tax_Id=36329	60188	Plasmodium falciparum	288.86	12	2	4	289.18	9	2	4				
Q8I5F4		PFL1270w Cof-like hydrolase, had-superfamily, subfamily iib Tax_Id=36329	33268	Plasmodium falciparum	288.86	14	2	4	289.18	14	2	4				
Q8IAM2	1-cyspxn	1-cyspxn 1-cys peroxiredoxin Tax_Id=36329	25164	Plasmodium falciparum	288.86	21	2	5	289.18	21	2	5				
Q8IEU2	pfg27-25	pfg27-25 Plasmodium falciparum gamete antigen 27/25 Tax_Id=36329	26004	Plasmodium falciparum	288.86	10	2	4	289.18	10	2	4				
Q9NLB2	GRX1	GRX1 Glutaredoxin Tax_Id=36329	12418	Plasmodium falciparum	288.86	18	2	2	289.18	18	2	2				
C6KTB1		PFF1335c 4-methyl-5(B-hydroxyethyl)-thiazol monophosphate biosynthesis enzym	20292	Plasmodium falciparum	288.86	23	2	3	289.18	23	2	3				
Q8IBF2		MAL7P1.171 Uncharacterized protein Tax_Id=36329	244195	Plasmodium falciparum	288.86	3	2	10	289.18	3	2	10				
Q8IIU7		PF11_0067 Conserved Plasmodium protein Tax_Id=36329	90787	Plasmodium falciparum	288.86	4	2	2	289.18	4	2	2				
Q8I2Q0		PFI1270w Putative uncharacterized protein Tax_Id=36329	24698	Plasmodium falciparum	288.74	8	2	2	287.33	8	2	2				
C0H4V6		MAL8P1.69 14-3-3 protein, putative Tax_Id=36329	30192	Plasmodium falciparum	284.18	23	3	4	282.82	23	3	4				
Q8II72		PF11_0302 Conserved Plasmodium protein Tax_Id=36329	51951	Plasmodium falciparum	283.11	8	2	2	276.71	5	2	2				
O96127		PFB0115w Conserved Plasmodium protein Tax_Id=36329	141848	Plasmodium falciparum	282.53	3	2	2	278.97	2	2	2				
P61076	trxr2	trxr2 Thioredoxin reductase 2 Tax_Id=36329	68697	Plasmodium falciparum	275.46	7	2	2	272.44	7	2	2				
Q8IIR7	ERC	ERC Endoplasmic reticulum-resident calcium binding protein Tax_Id=36329	39374	Plasmodium falciparum	239.02	10	2	3	236.56	10	2	3				
Q8I4T7		PFL2385c Conserved Plasmodium protein Tax_Id=36329	91947	Plasmodium falciparum	229.67	3	1	14	224.48	3	1	10	58.3	3	1	3
C0H4C9		PFE0320w Uncharacterized protein Tax_Id=36329	469873	Plasmodium falciparum	220.18	5	3	3					136.55	4	3	3
Q6LFH8	OAT	OAT Ornithine aminotransferase Tax_Id=36329	46055	Plasmodium falciparum	192.58	12	2	4	191.72	12	2	2	154.26	9	1	1
Q8IFP3		PFD1050w Alpha-tubulin ii Tax_Id=36329	49691	Plasmodium falciparum	185.23	5	2	4	182.74	5	2	4				
O77365		PFC0485w Protein kinase, putative Tax_Id=36329	295497	Plasmodium falciparum	176.84	3	2	2					90.45	2	2	2
C6KT82		PFF1185w Smarca-related protein Tax_Id=36329	315620	Plasmodium falciparum	168.73	3	2	8	169.85	2	2	4	86.53	2	1	4
Q8I467		PFE0165w Cofilin/actin-depolymerizing factor homolog 1 Tax_Id=36329	13740	Plasmodium falciparum	161.42	25	2	4	155.04	25	2	3	154.26	18	1	1
O97319		PFC0870w Elongation factor 1 (EF-1), putative Tax_Id=36329	17706	Plasmodium falciparum	160.76	7	1	2	160.87	7	1	2				
Q8IJF5		PF10_0243 Conserved Plasmodium protein Tax_Id=36329	190400	Plasmodium falciparum	155.69	2	2	13	85.95	2	1	4	154.26	2	1	8
C0H4C1		PFE0128c Uncharacterized protein Tax_Id=36329	107744	Plasmodium falciparum	154.43	3	1	32	154.59	3	1	18	128.48	1	1	14
C0H581		PFI1540w Putative uncharacterized protein Tax_Id=36329	85234	Plasmodium falciparum	154.43	2	1	36	154.59	2	1	20	92.16	2	1	12
C0H5H4		MAL13P1.226 Uncharacterized protein Tax_Id=36329	174490	Plasmodium falciparum	154.43	1	1	4					113.13	1	1	4
C6KTA3		PFF1295w Uncharacterized protein Tax_Id=36329	43649	Plasmodium falciparum	154.43	6	1	1	154.59	6	1	1				
O77368	PfATPase7	PfATPase7 P-type ATPase, putative Tax_Id=36329	218020	Plasmodium falciparum	154.43	3	1	1	154.59	3	1	1				
O96130	OPP	OPP Octaprenyl pyrophosphate synthase Tax_Id=36329	63045	Plasmodium falciparum	154.43	3	1	1	154.37	3	1	1				
P46468		PF07_0047 Putative cell division cycle ATPase Tax_Id=36329	142080	Plasmodium falciparum	154.43	4	1	1	154.59	3	1	1				
P61074	PCNA	PCNA Proliferating cell nuclear antigen Tax_Id=36329	30586	Plasmodium falciparum	154.43	9	1	1	154.59	5	1	1				
Q76NM3	PfLDH	PfLDH L-lactate dehydrogenase Tax_Id=36329	34107	Plasmodium falciparum	154.43	4	1	5	154.59	4	1	4	154.26	4	1	1
Q76NN6		PFD0950w Ran binding protein 1, putative Tax_Id=36329	33195	Plasmodium falciparum	154.43	10	1	2	154.59	10	1	2				
Q7KQK0	PKAr	PKAr CAMP-dependent protein kinase regulatory subunit, putative Tax_Id=36329	50832	Plasmodium falciparum	154.43	6	1	2	154.59	6	1	2				
Q7KQK5	AMA1	AMA1 Apical membrane antigen 1, AMA1 Tax_Id=36329	72042	Plasmodium falciparum	154.43	4	1	1	154.59	2	1	1				
Q7KQK6	Ran	Ran GTP-binding nuclear protein ran/tc4 Tax_Id=36329	24875	Plasmodium falciparum	154.43	22	1	3	154.59	16	1	3				

Q8I288		PFA_0260c Adenylyl cyclase-associated protein Tax_Id=36329	18528	Plasmodium falciparum	154.43	4	1	3	154.59	4	1	3				
Q8I3U0		PFE0840c Transcription factor with AP2 domain(S), putative Tax_Id=36329	276424	Plasmodium falciparum	154.43	1	1	9	154.59	1	1	3	106.76	1	1	6
Q8I3Z5	TCTP	TCTP Translationally-controlled tumor protein homolog Tax_Id=36329	19979	Plasmodium falciparum	154.43	8	1	2	154.59	8	1	2				
Q8I5D7		PFL1360c Conserved Plasmodium protein Tax_Id=36329	52885	Plasmodium falciparum	154.43	7	1	2	79.08	7	1	1	127.69	5	1	1
Q8I638	ACS7	ACS7 Acyl-CoA synthetase, PfACS7 Tax_Id=36329	104621	Plasmodium falciparum	154.43	3	1	2	154.59	3	1	2				
Q8I6T3		PF13_0156 Proteasome subunit beta type Tax_Id=36329	29961	Plasmodium falciparum	154.43	13	1	1	154.59	13	1	1				
Q8IAX8		PF08_0074 DNA/RNA-binding protein Alba, putative Tax_Id=36329	27258	Plasmodium falciparum	154.43	4	1	1	154.59	4	1	1				
Q8IB77		MAL8P1.33 GTP binding protein, putative Tax_Id=36329	91193	Plasmodium falciparum	154.43	7	1	3	154.59	3	1	3				
Q8IBN7		MAL7P1.114 P36-like protein homologue, putative Tax_Id=36329	24792	Plasmodium falciparum	154.43	9	1	1	154.59	9	1	1				
Q8ID66	PF92	PF92 Merozoite surface protein P92 Tax_Id=36329	92727	Plasmodium falciparum	154.43	3	1	1	154.59	3	1	1				
Q8IDC6		MAL13P1.284 Pyrroline carboxylate reductase Tax_Id=36329	28379	Plasmodium falciparum	154.43	16	1	1	154.59	9	1	1				
Q8IEH3		MAL13P1.73 Uncharacterized protein Tax_Id=36329	89232	Plasmodium falciparum	154.43	3	1	1	154.59	2	1	1				
Q8II43		PF11_0331 T-complex protein 1 subunit alpha Tax_Id=36329	60260	Plasmodium falciparum	154.43	4	1	1	154.59	4	1	1				
Q8II81		PF11_0293 Multiprotein bridging factor type 1, putative Tax_Id=36329	15525	Plasmodium falciparum	154.43	15	1	1	154.59	15	1	1				
Q8IIA4		PF11_0270 Threonine--tRNA ligase, putative Tax_Id=36329	119545	Plasmodium falciparum	154.43	3	1	1	154.59	3	1	1				
Q8IIB7		PF11_0257 Ethanolamine kinase, putative Tax_Id=36329	49914	Plasmodium falciparum	154.43	5	1	1	154.59	5	1	1				
Q8IIJ9		PF11_0174 Probable cathepsin C Tax_Id=36329	80412	Plasmodium falciparum	154.43	4	1	1	154.59	4	1	1				
Q8IIR3		PF11_0540a Conserved Plasmodium protein Tax_Id=36329	175053	Plasmodium falciparum	154.43	3	1	5	154.59	2	1	3	124.97	1	1	2
Q8IJ52		PF10_0348 Erythrocyte membrane protein, putative Tax_Id=36329	80247	Plasmodium falciparum	154.43	2	1	1	154.59	2	1	1				
Q8IKX8		PF14_0473 Rrp6 homologue, putative Tax_Id=36329	135628	Plasmodium falciparum	154.43	2	1	1	154.59	2	1	1				
Q8ILB9		PF14_0326 Dynein-related AAA-type ATPase Tax_Id=36329	971015	Plasmodium falciparum	154.43	2	1	1					98.85	2	1	1
Q8IM77		PF14_0013 Uncharacterized J domain-containing protein PF14_0013 Tax_Id=3632	57093	Plasmodium falciparum	154.43	5	1	3	147.69	5	1	2	120.42	2	1	1
Q8IBT4		PF07_0068 Cysteine desulfurase, putative Tax_Id=36329	64561	Plasmodium falciparum	152.01	7	1	4	107.25	7	1	2	42.44	7	1	1
C0H534		PFI0565w Putative uncharacterized protein Tax_Id=36329	61134	Plasmodium falciparum	151.08	6	1	1	148.21	6	1	1				
Q8I240		PFA_0510w Bromodomain protein, putative Tax_Id=36329	268207	Plasmodium falciparum	151.05	2	1	3	134.53	2	1	2	84.59	2	1	1
Q8IDA0	gc-betA	gc-betA Guanylyl cyclase Tax_Id=36329	376990	Plasmodium falciparum	150.02	3	2	7	141.24	2	2	3	31.47	2	1	4
Q8IIN2		PF11_0528 Conserved Plasmodium protein Tax_Id=36329	710223	Plasmodium falciparum	145.6	3	2	10	147.77	2	2	6	32.53	2	1	1
Q8IBA0	PfRACK	PfRACK Receptor for activated C kinase homolog, PfRACK Tax_Id=36329	35686	Plasmodium falciparum	142.62	7	1	1	134.4	3	1	1				
O77392		MAL3P6.3 Probable inorganic pyrophosphatase Tax_Id=36329	45232	Plasmodium falciparum	142.32	2	1	1	147.68	2	1	1				
Q8ILI2		PF14_0261 Proliferation-associated protein 2g4, putative Tax_Id=36329	42641	Plasmodium falciparum	142.3	6	1	1	133.35	6	1	1				
O97291		PFC0960c Putative uncharacterized protein Tax_Id=36329	231793	Plasmodium falciparum	142.18	4	1	5	74.47	3	1	3	52.31	2	1	2
C0H4B3		PFD0872w Uncharacterized protein Tax_Id=36329	582756	Plasmodium falciparum	140.58	3	2	3	97.08	2	1	1	32.81	1	1	2
Q8IBK6		MAL7P1.132 Uncharacterized protein Tax_Id=36329	243554	Plasmodium falciparum	140.15	1	1	1					82.55	1	1	1
Q8ILR9		PF14_0175 Protein PF14_0175 Tax_Id=36329	548870	Plasmodium falciparum	138.75	2	1	14	113	1	1	11	26.02	1	1	2
Q8I408		PFE0475w Asparagine-tRNA ligase, putative Tax_Id=36329	85249	Plasmodium falciparum	138.22	1	1	14	131.39	1	1	7	77.53	1	1	7
O97283		PFC0905c Putative uncharacterized protein Tax_Id=36329	360522	Plasmodium falciparum	135.45	2	1	2	70.71	2	1	1	41.48	1	1	1
C6KSZ3		PFF0735w Uncharacterized protein Tax_Id=36329	45768	Plasmodium falciparum	134.56	10	1	6	136.76	10	1	2	62.89	7	1	3
Q8IET8		MAL13P1.14 ATP dependent DEAD-box helicase, putative Tax_Id=36329	183685	Plasmodium falciparum	134.18	3	2	2	138.33	3	2	2				
Q8ILX7		PF14_0116 Putative uncharacterized protein Tax_Id=36329	56907	Plasmodium falciparum	132.8	3	1	5	90.62	3	1	4	34.32	3	1	1
Q9TY96	SERA-6	SERA-6 Serine repeat antigen 6 (SERA-6) Tax_Id=36329	118836	Plasmodium falciparum	130.03	2	1	1	126.6	2	1	1				
C6KSS6		PFF0385c Uncharacterized protein Tax_Id=36329	77689	Plasmodium falciparum	128.84	2	1	2	32.65	2	1	1	77.74	2	1	1
Q8IL26		PF14_0423 GCN2 alpha-related protein kinase Tax_Id=36329	184251	Plasmodium falciparum	128.61	4	1	1					70.08	3	1	1
Q8IIL1		PF11_0160 Conserved Plasmodium protein Tax_Id=36329	94293	Plasmodium falciparum	126.87	4	1	1	124.79	4	1	1				
Q8ILF2		PF14_0292 Cytosolic preribosomal GTPase, putative Tax_Id=36329	98316	Plasmodium falciparum	126.52	3	1	1					69.56	3	1	1
Q8IID5		PF11_0239 Calcium-dependent protein kinase, putative Tax_Id=36329	191814	Plasmodium falciparum	125.57	2	1	3	50.06	1	1	1	71.28	1	1	2
Q8ILG1		PF14_0282 Putative uncharacterized protein Tax_Id=36329	306807	Plasmodium falciparum	124.15	3	1	1	126.88	2	1	1				
C0H5H9		PF13_0264 Ubiquitin-activating enzyme E1, putative Tax_Id=36329	85105	Plasmodium falciparum	124.1	4	1	4	122.6	4	1	3	52.15	4	1	1
C6S3B5		PFB0467w 50S ribosomal protein L33, putative Tax_Id=36329	14158	Plasmodium falciparum	123.99	10	1	1					70.59	10	1	1
Q8IJ49		PF10_0351 Probable protein Tax_Id=36329	65151	Plasmodium falciparum	123.67	10	1	1	117.65	10	1	1				
C0H4P4		MAL7P1.146 Uncharacterized protein Tax_Id=36329	599325	Plasmodium falciparum	123.44	1	2	2	120.85	1	2	2				
C6KSZ6	Pfcrk-5	Pfcrk-5 Cyclin-dependent protein kinase, predicted Tax_Id=36329	82947	Plasmodium falciparum	123.32	1	1	1	116.48	1	1	1				
Q8IL46		PF14_0403 Protein prenyltransferase alpha subunit, putative Tax_Id=36329	87483	Plasmodium falciparum	121.52	3	1	1					67	2	1	1

Q8IIH3		PF11_0201 Ubiquitin-protein ligase, putative Tax_Id=36329	256822	Plasmodium falciparum	119.75	2	1	8	70.84	2	1	5	50.04	1	1	3
O77320		PFC0335c Putative uncharacterized protein Tax_Id=36329	448208	Plasmodium falciparum	119.22	3	1	1					66	1	1	1
Q8I581		PFL1645w Conserved Plasmodium protein Tax_Id=36329	466016	Plasmodium falciparum	118.88	1	1	1	125.8	1	1	1				
O96228		PFB0675w Conserved Plasmodium membrane protein Tax_Id=36329	160635	Plasmodium falciparum	117.63	3	1	2	115.09	2	1	1	67.73	2	1	1
Q8IJI6		PF10_0211 Conserved Plasmodium membrane protein Tax_Id=36329	830196	Plasmodium falciparum	117.48	2	2	3	110.86	1	2	2	46.53	1	1	1
Q8I3A3		PFI0225w Ubiquitin specific protease, putative Tax_Id=36329	207568	Plasmodium falciparum	116.64	4	1	1	110.06	3	1	1				
C0H491		PFD0320c Uncharacterized protein Tax_Id=36329	401566	Plasmodium falciparum	115.54	4	1	1	111.43	3	1	1				
Q8I2V5		PFI0970c TLD domain-containing protein Tax_Id=36329	124916	Plasmodium falciparum	115.25	2	1	4	42.8	2	1	1	64.02	1	1	3
Q8I3M8		PFE1180c Uncharacterized protein Tax_Id=36329	178974	Plasmodium falciparum	115.14	1	1	1					66.7	1	1	1
Q8IJQ9		PF10_0133 Conserved Plasmodium protein Tax_Id=36329	269272	Plasmodium falciparum	114	3	1	2	105.45	3	1	1	26.63	1	1	1
Q8ILA2		PF14_0343 Putative uncharacterized protein Tax_Id=36329	270478	Plasmodium falciparum	112.15	2	1	1					66.04	1	1	1
C6KSN6		PFF0185c Uncharacterized protein Tax_Id=36329	219258	Plasmodium falciparum	111.72	2	1	2					60.93	1	1	2
Q8I455		PFE0230w Uncharacterized protein Tax_Id=36329	291039	Plasmodium falciparum	110.94	2	1	8	115.18	2	1	4	53.56	1	1	5
Q8IDZ8	PfCpn20	PfCpn20 Cochaperonin Tax_Id=36329	29063	Plasmodium falciparum	110.71	13	1	1					61.75	13	1	1
Q8I569		PFL1705w RNA binding protein, putative Tax_Id=36329	194674	Plasmodium falciparum	110.5	1	1	1	106.17	1	1	1				
Q8I2P1		PFI1315c Putative uncharacterized protein Tax_Id=36329	144989	Plasmodium falciparum	109.79	5	1	1	114.78	5	1	1				
Q8I374		PFI0370c Subunit of proteaseome activator complex, putative Tax_Id=36329	33125	Plasmodium falciparum	108.13	10	1	1					63.26	10	1	1
C6KTB8	PfPK4	PfPK4 Protein kinase PK4 Tax_Id=36329	364331	Plasmodium falciparum	106.79	2	1	5	106.08	2	1	3	20.91	1	1	1
Q8IKT7		PF14_0515 Putative uncharacterized protein Tax_Id=36329	168933	Plasmodium falciparum	103.26	2	1	1	98.92	2	1	1				
C6KSQ4		PFF0275c Nucleoside diphosphate kinase, putative Tax_Id=36329	219869	Plasmodium falciparum	102.7	1	1	4	103.79	0	1	1	46.02	1	1	3
Q8I3T8		PFE0850c 60S ribosomal protein L12, putative Tax_Id=36329	18112	Plasmodium falciparum	100.55	5	1	3	96.61	5	1	3				
Q7KQL5		PF10_0084 Tubulin beta chain Tax_Id=36329	49751	Plasmodium falciparum	100.17	4	1	1	91.87	4	1	1				
C0H5D6		MAL13P1.140 Uncharacterized protein Tax_Id=36329	325123	Plasmodium falciparum	99.48	3	1	1	99.29	2	1	1				
Q8IBR9	PfEST	PfEST Exported serine/threonine protein kinase Tax_Id=36329	317692	Plasmodium falciparum	99.26	4	2	2	35.28	3	1	1	45.36	2	1	1
C0H4L1		MAL7P1.202 Uncharacterized protein Tax_Id=36329	145509	Plasmodium falciparum	97.51	2	1	1	93.85	2	1	1				
C0H4J3		MAL7P1.225.1 Uncharacterized protein Tax_Id=36329	34344	Plasmodium falciparum	95.71	3	1	1	98.37	3	1	1				
Q8IKI5		PF14_0620 Metal-dependent hydrolase, putative Tax_Id=36329	100044	Plasmodium falciparum	95.67	1	1	2	98.52	1	1	2				
Q8IDY5		PF13_0191 Uncharacterized protein Tax_Id=36329	54351	Plasmodium falciparum	95.22	2	1	1	91.32	2	1	1				
Q8IDH3		PF13_0273 Uncharacterized protein Tax_Id=36329	304306	Plasmodium falciparum	95.16	1	2	2	92.47	1	2	2				
Q8ILX0		PF14_0123 Putative uncharacterized protein Tax_Id=36329	390696	Plasmodium falciparum	93.13	2	1	13	89.8	1	1	8	58.05	1	1	3
Q8IE71		PF13_0139 Uncharacterized protein Tax_Id=36329	201043	Plasmodium falciparum	93.04	4	1	1					56.21	4	1	1
O96197	TKL-1	TKL-1 Protein kinase, putative Tax_Id=36329	148227	Plasmodium falciparum	92.25	3	1	1	90.69	1	1	1				
C6KSU1	PfCLS	PfCLS Mitochondrial cardiolipin synthase, PfCLS Tax_Id=36329	71207	Plasmodium falciparum	92.11	8	1	1	95.24	4	1	1				
Q8IEM5		PF13_0048 NUDIX hydrolase, putative Tax_Id=36329	137527	Plasmodium falciparum	91.79	7	1	2	32.37	2	1	1	41.67	7	1	1
C0H4Q1		PF07_0120 Uncharacterized protein Tax_Id=36329	304521	Plasmodium falciparum	88.56	3	1	1					51.18	2	1	1
C6KTD8		PFF1470c DNA polymerase epsilon, catalytic subunit a, putative Tax_Id=36329	344607	Plasmodium falciparum	88.31	2	1	2	35.01	2	1	1	51.95	2	1	1
Q8IBJ5		PF07_0107 Uncharacterized protein Tax_Id=36329	95179	Plasmodium falciparum	87.25	3	1	2					50.08	3	1	2
Q8I5T2		PFL0595c Glutathione peroxidase Tax_Id=36329	23952	Plasmodium falciparum	84.17	4	1	1	87.69	4	1	1				
C0H563		PFI1285w Protein kinase, putative Tax_Id=36329	141140	Plasmodium falciparum	83.34	1	1	1	81.24	1	1	1				
Q8IHT2		PF11_0447 Translation initiation factor eIF-1A, putative Tax_Id=36329	17866	Plasmodium falciparum	83.32	10	1	2	74.44	10	1	2				
C0H4F8		PFE1120w Uncharacterized protein Tax_Id=36329	1117187	Plasmodium falciparum	82.53	4	2	6	42.47	2	1	3	37.11	2	1	1
Q8I3Q8		PFE1015c Uncharacterized protein Tax_Id=36329	82395	Plasmodium falciparum	82.39	4	1	1					45.37	3	1	1
Q8IBA5		PF08_0016 Uncharacterized protein Tax_Id=36329	100939	Plasmodium falciparum	81.99	7	1	1	76.9	6	1	1				
Q8IDE2		PF13_0298 Uncharacterized protein Tax_Id=36329	166279	Plasmodium falciparum	80.71	2	1	2	81.3	2	1	2				
Q8I431		PFE0350c 60S ribosomal protein L4, putative Tax_Id=36329	46211	Plasmodium falciparum	80.45	4	1	1					42.88	4	1	1
Q8I620		PFL0125c Conserved Plasmodium protein Tax_Id=36329	130324	Plasmodium falciparum	79.7	3	1	2	70.54	2	1	1	42.46	2	1	1
Q8I542		PFL1845c Calcyclin binding protein, putative Tax_Id=36329	26643	Plasmodium falciparum	79.52	11	1	1	78.99	4	1	1				
B9ZSJ6		PFD0965W Phosphatidylinositol 4-kinase, putative Tax_Id=36329	611506	Plasmodium falciparum	79.45	2	1	1	78.71	2	1	1				
C6S3H2		PF14_0031a Putative uncharacterized protein Tax_Id=36329	229535	Plasmodium falciparum	79.1	4	1	1	83.49	3	1	1				
Q8IKJ5		PF14_0610 Zinc finger protein, putative Tax_Id=36329	39929	Plasmodium falciparum	77.88	7	1	6	72.86	7	1	3	30.48	4	1	3
C6KSU6		PFF0490w Uncharacterized protein Tax_Id=36329	244672	Plasmodium falciparum	77.3	2	1	1	69.06	2	1	1				
Q8I350		PFI0495w Putative uncharacterized protein Tax_Id=36329	368345	Plasmodium falciparum	76.13	3	1	1					45.68	2	1	1

Q8IKR4		PF14_0538 Putative uncharacterized protein Tax_Id=36329	283202	Plasmodium falciparum	76.03	1	1	1					43.73	1	1	1
Q8I5H1		PFL1185c Cytochrome c heme lyase, putative Tax_Id=36329	27717	Plasmodium falciparum	75.27	16	1	1	71.34	8	1	1				
Q9TY98		PFB0190c Conserved Plasmodium protein Tax_Id=36329	271490	Plasmodium falciparum	75.21	2	1	1					44.32	2	1	1
Q9U0G6	VAR	VAR Erythrocyte membrane protein 1, PfEMP1 Tax_Id=36329	250780	Plasmodium falciparum	75.09	3	1	4	74.21	3	1	3	27.18	2	1	1
Q8I6U9		PF14_0597 Cytochrome c1, putative Tax_Id=36329	46186	Plasmodium falciparum	74.55	5	1	4					43.29	5	1	3
Q8I2W7		PFI0910w DNA helicase, putative Tax_Id=36329	85509	Plasmodium falciparum	73.91	9	1	4	58.88	7	1	1	43.07	4	1	3
Q8IL57		PF14_0392 Serine/threonine protein kinase, putative Tax_Id=36329	263794	Plasmodium falciparum	72.29	5	1	1					42.2	2	1	1
C6KSV4		PFF0535c Transcription elongation factor SPT5 Tax_Id=36329	149366	Plasmodium falciparum	71.06	3	1	1					40.82	1	1	1
Q8IJ32		PF10_0368 Dynamin-like protein Tax_Id=36329	81526	Plasmodium falciparum	70.53	5	1	1	65.01	5	1	1				
C0H5D9		PF13_0148 Uncharacterized protein Tax_Id=36329	575464	Plasmodium falciparum	69.94	2	1	6	70.66	2	2	5	39.29	1	1	1
C0H4I8		MAL7P1.220 Uncharacterized protein Tax_Id=36329	23707	Plasmodium falciparum	65.52	5	1	1					38.73	5	1	1
Q8I3M2		PFE1210c Uncharacterized protein Tax_Id=36329	49181	Plasmodium falciparum	64.09	8	1	1	62.35	8	1	1				
Q8IDA6		MAL13P1.298 Uncharacterized protein Tax_Id=36329	249760	Plasmodium falciparum	62.47	4	1	3	66.24	3	1	2				
Q8IE81		PF13_0134 Uncharacterized protein Tax_Id=36329	48616	Plasmodium falciparum	62.29	4	1	2	61.45	4	1	2				
Q8I207		PFD0080c Uncharacterized protein Tax_Id=36329	60270	Plasmodium falciparum	62.19	3	1	1	62.29	3	1	1				
C0H487		PFD0160w Uncharacterized protein Tax_Id=36329	460087	Plasmodium falciparum	61.61	3	1	3	56.99	3	1	2	35.4	1	1	1
Q8IBX0	VAR	VAR Erythrocyte membrane protein 1, PfEMP1 Tax_Id=36329	238468	Plasmodium falciparum	61.61	3	1	1	61.95	2	1	1				
Q8IK03		PF10_0037 Conserved Plasmodium protein Tax_Id=36329	243183	Plasmodium falciparum	61.61	3	1	1	52.4	2	1	1				
Q8IBY8		PF07_0042 Uncharacterized protein Tax_Id=36329	350703	Plasmodium falciparum	60.55	3	1	1					34.81	1	1	1
Q8IL54		PF14_0395 Acid cluster protein 33 homologue, putative Tax_Id=36329	67622	Plasmodium falciparum	60.05	7	1	1	64.87	5	1	1				
Q8IKM3		PF14_0581 Apicoplast ribosomal protein S10, putative Tax_Id=36329	32584	Plasmodium falciparum	58.23	7	1	1	56.04	7	1	1				
C6S3B0		PFB0227c Conserved Plasmodium protein Tax_Id=36329	86236	Plasmodium falciparum	58.16	8	1	4					26.73	4	1	4
Q8I3J1		PFE1365w Uncharacterized protein Tax_Id=36329	20808	Plasmodium falciparum	58.09	6	1	1					31.97	6	1	1
Q8ILT5		PF14_0159 Protein SEY1 homolog Tax_Id=36329	110609	Plasmodium falciparum	57.29	2	1	1	55.7	2	1	1				
C6KST7		PFF0445w Uncharacterized protein Tax_Id=36329	720516	Plasmodium falciparum	57.18	1	1	1					34.29	1	1	1
Q8I2N6		PFI1340w Fumarate hydratase, putative Tax_Id=36329	78396	Plasmodium falciparum	54.71	3	1	1	56.62	3	1	1				
Q8IKK6		PF14_0599 Putative uncharacterized protein Tax_Id=36329	97668	Plasmodium falciparum	54.67	4	1	1					33.54	4	1	1
Q8IIG9		PF11_0205 Alternative splicing factor ASF-1 Tax_Id=36329	23652	Plasmodium falciparum	54.39	26	1	1	55.18	26	1	1				
Q8I259		PFA_0410w Putative uncharacterized protein Tax_Id=36329	255042	Plasmodium falciparum	53.92	1	1	2	23.18	1	1	1	32.79	1	1	1
Q8IDL5		PF13_0254 Uncharacterized protein Tax_Id=36329	460860	Plasmodium falciparum	53.33	1	1	1					32.27	1	1	1
Q8IBM5		PF07_0094 Uncharacterized protein Tax_Id=36329	66301	Plasmodium falciparum	52.58	4	1	2	55.15	4	1	2				
Q8IC14		MAL7P1.22 Uncharacterized protein Tax_Id=36329	194410	Plasmodium falciparum	51.92	2	1	1	43.66	2	1	1				
Q8I4P3	RIF	RIF Rifin Tax_Id=36329	41801	Plasmodium falciparum	51.8	4	1	1					31.4	4	1	1
Q8IL55		PF14_0394 Putative uncharacterized protein Tax_Id=36329	58371	Plasmodium falciparum	49.98	4	1	1					27.65	4	1	1
Q8IM32		PF14_0059 Putative uncharacterized protein Tax_Id=36329	338381	Plasmodium falciparum	49.85	1	1	1	48	1	1	1				
Q8I519	VAR	VAR Erythrocyte membrane protein 1, PfEMP1 Tax_Id=36329	261604	Plasmodium falciparum	49.06	3	1	1	49.7	3	1	1				
Q8IJW1		PF10_0080 Endonuclease, putative Tax_Id=36329	45595	Plasmodium falciparum	48.89	8	1	2	47.58	8	1	2				
Q8IHM8		PF11_0512 RESA-like protein with PHIST and DnaJ domains Tax_Id=36329	95492	Plasmodium falciparum	48.43	6	1	1	47.93	6	1	1				
Q8IDT8		MAL13P1.202 Uncharacterized protein Tax_Id=36329	231557	Plasmodium falciparum	46.98	3	1	2					27.19	1	1	2
Q8I1X5		PFD0265w Pre-mRNA splicing factor, putative Tax_Id=36329	366398	Plasmodium falciparum	46.23	2	1	3					27.63	1	1	3
Q9NF98		PFC1016w Putative uncharacterized protein Tax_Id=36329	21787	Plasmodium falciparum	45.72	8	1	1	44.77	8	1	1				
Q8IC27		PF07_0016 Uncharacterized protein Tax_Id=36329	218376	Plasmodium falciparum	45.61	6	1	1					29.74	4	1	1
C0H4Y5		PF08_0014 Apicoplast ribosomal protein L21, putative Tax_Id=36329	38509	Plasmodium falciparum	45.36	3	1	3	40.92	3	1	2	28.39	3	1	1
C0H468		PFC0080c Putative uncharacterized protein Tax_Id=36329	87508	Plasmodium falciparum	45.27	8	1	1	43.48	4	1	1				
Q8IKT2		PF14_0520 6-phosphogluconate dehydrogenase, decarboxylating Tax_Id=36329	52994	Plasmodium falciparum	45.23	4	1	1	45.65	3	1	1				
Q8IIU3		PF11_0071 RuvB DNA helicase, putative Tax_Id=36329	53406	Plasmodium falciparum	45.1	3	1	1	40.67	3	1	1				
Q8IJW3		PF10_0078 Histone deacetylase, putative Tax_Id=36329	282227	Plasmodium falciparum	44.54	1	1	2	42.29	1	1	2				
C0H4Q9		MAL8P1.151 Inositol phosphatase, putative Tax_Id=36329	173061	Plasmodium falciparum	44.3	3	1	1	43.35	3	1	1				
Q8IB64		MAL8P1.42 Uncharacterized protein Tax_Id=36329	162313	Plasmodium falciparum	44.17	2	1	1	46.06	2	1	1				
Q8I5W5	PCRMP 3	PCRMP Cysteine repeat modular protein 3, putative Tax_Id=36329	418846	Plasmodium falciparum	44.02	3	1	1	39.77	2	1	1				
Q8IM05		PF14_0088 Aldo/keto reductase, putative Tax_Id=36329	143353	Plasmodium falciparum	43.96	4	1	1	45.02	3	1	1				
Q8IBW7	VAR	VAR Erythrocyte membrane protein 1, PfEMP1 Tax_Id=36329	248942	Plasmodium falciparum	43.93	2	1	1	44.72	2	1	1				

Q8I3V8		PFE0750c RNA recognition motif, putative Tax_Id=36329	98382	Plasmodium falciparum	43.18	3	1	1					25.93	3	1	1
Q8I545		PFL1830w Ubiquitin-like protein, putative Tax_Id=36329	8601	Plasmodium falciparum	43.01	16	1	1					25.26	16	1	1
Q8ID92		PF13_0326 Cofilin/actin-depolymerizing factor homolog 2 Tax_Id=36329	16997	Plasmodium falciparum	42.74	10	1	1					21.09	10	1	1
Q76NM5	eba-140	eba-140 Erythrocyte binding antigen-140 Tax_Id=36329	140596	Plasmodium falciparum	42.55	5	1	1	38.81	4	1	1				
O97331		PFC0075c Putative uncharacterized protein Tax_Id=36329	34316	Plasmodium falciparum	42.28	4	1	1	42.29	4	1	1				
Q8IL71		PF14_0377 Vesicle-associated membrane protein, putative Tax_Id=36329	27686	Plasmodium falciparum	42.07	5	1	1	38.51	5	1	1				
Q8IE35		PF13_0161 Uncharacterized protein Tax_Id=36329	205477	Plasmodium falciparum	41.56	3	1	1					25.28	2	1	1
Q8IIZ6		PF10_0406 Erythrocyte membrane protein 1, PfEMP1 Tax_Id=36329	248436	Plasmodium falciparum	41.52	3	1	1					24.06	3	1	1
Q8IBB3		PF08_0011 Leucine-tRNA ligase Tax_Id=36329	178430	Plasmodium falciparum	41.15	3	1	1					23.25	2	1	1
Q8IJ55	SPAM	SPAM Merozoite surface protein 3 Tax_Id=36329	40119	Plasmodium falciparum	41.14	3	1	1	43.04	3	1	1				
Q8I494	RIF	RIF Rifin Tax_Id=36329	41779	Plasmodium falciparum	40.39	9	1	1					22.35	6	1	1
Q8II22		PF11_0353 Conserved Plasmodium protein Tax_Id=36329	214592	Plasmodium falciparum	40.28	3	1	2	39.14	2	1	1				
Q8IKP8		PF14_0556 Putative uncharacterized protein Tax_Id=36329	183546	Plasmodium falciparum	40.25	4	1	1					23.53	3	1	1
Q8I338		PFI0555c Putative uncharacterized protein Tax_Id=36329	147227	Plasmodium falciparum	39	5	1	1					23.51	2	1	1
Q8I284		PFA_0280w Asparagine-rich antigen Pfa35-2 Tax_Id=36329	364073	Plasmodium falciparum	38.78	1	1	1					23.64	1	1	1
Q8IJD6		PF10_0262 Conserved Plasmodium protein Tax_Id=36329	196492	Plasmodium falciparum	38.58	4	1	3	31.08	2	1	1	23.85	3	1	1
Q8IHZ1		PF11_0385 UVB-resistance protein UVR8 homologue Tax_Id=36329	48114	Plasmodium falciparum	38.45	8	1	1	39.9	8	1	1				
Q8ILR6	UFD1	UFD1 Ubiquitin fusion degradation protein UFD1, putative Tax_Id=36329	32478	Plasmodium falciparum	36.82	5	1	1	38.56	5	1	1				
O77375		PFC0805w DNA-directed RNA polymerase Tax_Id=36329	278676	Plasmodium falciparum	36.24	2	1	6					21.95	1	1	6
Q8IK59		PF14_0747 Surface-associated intersprsed gene 14.1 (SURFIN 14.1) Tax_Id=36329	241735	Plasmodium falciparum	36.02	3	1	1	37.28	2	1	1				
Q8IBE9		MAL7P1.174 Uncharacterized protein Tax_Id=36329	37723	Plasmodium falciparum	36.01	10	1	1	38.79	10	1	1				
Q8I5M1		PFL0905c Conserved Plasmodium protein Tax_Id=36329	58797	Plasmodium falciparum	35.95	2	1	4	33.62	2	1	1				
Q8IJ92		PF10_0307 Conserved Plasmodium protein Tax_Id=36329	109276	Plasmodium falciparum	35.75	2	1	1					20.47	2	1	1
C6S3L1		PFL1970w Erythrocyte membrane protein 1, PfEMP1 Tax_Id=36329	247315	Plasmodium falciparum	35.74	2	1	1	43.11	2	1	1				
Q8IIV7		PF11_0056 Conserved Plasmodium protein Tax_Id=36329	76077	Plasmodium falciparum	35.61	3	1	1	36.98	3	1	1				
Q8I3L1		PFE1265w G-protein coupled receptor, putative Tax_Id=36329	55704	Plasmodium falciparum	35.32	6	1	1					21.55	6	1	1
C6KTB7		PFF1365c Putative E3 ubiquitin-protein ligase protein PFF1365c Tax_Id=36329	1206019	Plasmodium falciparum	35.29	3	1	1	33.61	2	1	1				
Q8I531	ApiAP2	ApiAP2 Transcription factor with AP2 domain(S), putative Tax_Id=36329	299381	Plasmodium falciparum	34.78	2	1	1	33.29	2	1	1				
O77384		PFC0760c Protein PFC0760c Tax_Id=36329	402952	Plasmodium falciparum	34.65	1	1	1					21.79	1	1	1
Q8IJ89		PF10_0310 Conserved Plasmodium protein Tax_Id=36329	106689	Plasmodium falciparum	34.44	3	1	2	25.7	3	1	1	23.74	2	1	1
C6KT56		PFF1055c Uncharacterized protein Tax_Id=36329	92452	Plasmodium falciparum	34.35	8	1	1	34.24	6	1	1				
Q8I303		PFI0730w BSD domain, putative Tax_Id=36329	84017	Plasmodium falciparum	34.35	4	1	1					21.95	4	1	1
Q8II83		PF11_0291 Conserved Plasmodium protein Tax_Id=36329	212865	Plasmodium falciparum	34.28	1	1	3	28.86	1	1	2				
Q8IM18		PF14_0073 Putative uncharacterized protein Tax_Id=36329	241131	Plasmodium falciparum	33.51	4	1	1	37.25	3	1	1				
C0H527		PFI0410c Putative uncharacterized protein Tax_Id=36329	241297	Plasmodium falciparum	33.32	3	1	1	32.13	1	1	1				
Q8IBA3		PF08_0018 Translation initiation factor-like protein Tax_Id=36329	164181	Plasmodium falciparum	33.29	0	1	4	29.79	0	1	3				
O77366		PFC0850c Endonuclease/exonuclease/phosphatase family protein, putative Tax_I	108306	Plasmodium falciparum	33.08	2	1	1	33.35	2	1	1				
C0H4P2		MAL7P1.134 Uncharacterized protein Tax_Id=36329	420676	Plasmodium falciparum	32.9	2	1	1	33.43	1	1	1				
C0H4F2		PFE1010w Protein phosphatase, putative Tax_Id=36329	83405	Plasmodium falciparum	32.61	3	1	2	20.29	3	1	1				
Q8IHV9		PF11_0417 Conserved Plasmodium protein Tax_Id=36329	219855	Plasmodium falciparum	32.11	5	1	1	30.97	5	1	1				
O96205		PFB0560w Conserved Plasmodium protein Tax_Id=36329	477854	Plasmodium falciparum	31.57	2	1	3	26.32	1	1	1	20.35	1	1	1
C0H4K5		MAL7P1.18 Serine/threonine protein kinase, putative Tax_Id=36329	192854	Plasmodium falciparum	31.55	1	1	1					20.99	1	1	1
C6KSV0		PFF0510w Histone H3 Tax_Id=36329	15446	Plasmodium falciparum	31.54	10	1	2	25.29	10	1	2				
Q8IDB6		MAL13P1.289 Mitotic control protein dis3 homologue, putative Tax_Id=36329	126662	Plasmodium falciparum	31.47	3	1	1	32.17	3	1	1				
Q8IKU7		PF14_0504 Putative uncharacterized protein Tax_Id=36329	110617	Plasmodium falciparum	31.4	6	1	3	30.97	4	1	3				
C6KST1		PFF0410w Uncharacterized protein Tax_Id=36329	233099	Plasmodium falciparum	29.7	1	1	1	29.99	1	1	1				
O97247		PFC0285c T-complex protein beta subunit, putative Tax_Id=36329	59068	Plasmodium falciparum	29.63	9	1	1	31.45	2	1	1				
Q8IES7		MAL13P1.19 Peptidase, putative Tax_Id=36329	1111794	Plasmodium falciparum	29.62	2	1	1	29.3	1	1	1				
C0H575		PFI1468c Leucine-rich repeat protein 8, LRR8 Tax_Id=36329	138632	Plasmodium falciparum	29.15	2	1	1	25.05	2	1	1				
O77339		PFC0560c Putative uncharacterized protein Tax_Id=36329	94883	Plasmodium falciparum	29.06	5	1	1	31.07	3	1	1				
Q8IIC8		PF11_0246 Conserved Plasmodium protein Tax_Id=36329	153698	Plasmodium falciparum	28.63	2	1	1	22.92	2	1	1				
Q8IDQ6		PF13_0237 Uncharacterized protein Tax_Id=36329	114585	Plasmodium falciparum	28.18	2	1	1	22.54	1	1	1				

Q8IL47		PF14_0402 Putative uncharacterized protein Tax_Id=36329	249604	Plasmodium falciparum	28.01	5	1	1	29.03	5	1	1				
Q8IBH9		PF07_0115 Cation transporting ATPase, cation transporter Tax_Id=36329	225077	Plasmodium falciparum	27.92	3	1	1					20.03	2	1	1
Q8IE42		PF13_0155 Uncharacterized protein Tax_Id=36329	320137	Plasmodium falciparum	27.55	6	1	1	29.14	4	1	1				
Q8IHU6		PF11_0431 Membrane skeletal protein, putative Tax_Id=36329	59605	Plasmodium falciparum	27.32	5	1	1	28.35	5	1	1				
C6KSY8		PFF0710w Uncharacterized protein Tax_Id=36329	72444	Plasmodium falciparum	26.75	10	1	1	23.76	10	1	1				
Q8ILW8		PF14_0125 Deoxyhypusine synthase Tax_Id=36329	57242	Plasmodium falciparum	26.68	2	1	1	31.24	2	1	1				
Q8IKQ6		PF14_0547 Putative uncharacterized protein Tax_Id=36329	371014	Plasmodium falciparum	26.5	3	1	2	30.56	2	1	1				
Q8I1Y6		PFD0207c Uncharacterized protein Tax_Id=36329	75373	Plasmodium falciparum	26.43	2	1	1	27.15	2	1	1				
C0H571	RhopH2	RhopH2 High molecular weight rhoptry protein-2 Tax_Id=36329	162665	Plasmodium falciparum	25.92	7	1	1	27.98	7	1	1				
C6KT28		PFF0910c Uncharacterized protein Tax_Id=36329	123082	Plasmodium falciparum	25.92	2	1	1	22.21	2	1	1				
C0H557		PFI1205c Putative uncharacterized protein Tax_Id=36329	154490	Plasmodium falciparum	25.26	3	1	1	22.02	3	1	1				
Q8IM55		PF14_0036 Phosphatase, putative Tax_Id=36329	35219	Plasmodium falciparum	25.16	4	1	1	30.98	4	1	1				
Q8I5X4		PFL0355c Conserved Plasmodium protein Tax_Id=36329	100169	Plasmodium falciparum	24.88	4	1	1	23.79	3	1	1				
Q8IEF3		PF13_0089 Inositol polyphosphate kinase, putative Tax_Id=36329	192246	Plasmodium falciparum	24.69	4	1	1	21.6	3	1	1				
Q8ILL5		PF14_0228 Putative uncharacterized protein Tax_Id=36329	183575	Plasmodium falciparum	24.58	5	1	1	20.42	4	1	1				
B3FEM7	PDEgamma	PDEgamma Calcium/calmodulin-dependent 3',5'-cyclic nucleotide phosphodiester	91680	Plasmodium falciparum	24.28	3	1	1	23.04	3	1	1				
Q8IJX6		PF10_0065 Conserved Plasmodium protein Tax_Id=36329	37798	Plasmodium falciparum	24.13	4	1	1	25.41	4	1	1				
Q8IIJ0		PF11_0184 DNA mismatch repair protein MLH1, putative Tax_Id=36329	118307	Plasmodium falciparum	23.73	4	1	1	27.3	4	1	1				
O96136		PFB0160w ERCC1 nucleotide excision repair protein, putative Tax_Id=36329	28287	Plasmodium falciparum	23.59	10	1	1	29.64	10	1	1				
Q8IBP4		MAL7P1.108 Phosphoinositide-binding protein, putative Tax_Id=36329	258426	Plasmodium falciparum	23.38	1	1	1	21.04	1	1	1				
Q8IIU6		PF11_0068 tRNA-splicing ligase RtcB homolog Tax_Id=36329	56321	Plasmodium falciparum	22.71	9	1	1	23.24	9	1	1				
Q8IDZ6		PF13_0182 Ubiquitin-activating enzyme, putative Tax_Id=36329	218525	Plasmodium falciparum	22.68	3	1	1	23.77	2	1	1				
Q8IDM8		PF13_0250 G-beta repeat protein, putative Tax_Id=36329	41374	Plasmodium falciparum	22.12	2	1	1	21.69	2	1	1				
O97265		PFC0630w Zinc finger protein, putative Tax_Id=36329	22473	Plasmodium falciparum	21.26	4	1	1	22.07	4	1	1				
Q8IHN1		PF11_0509 Ring-infected erythrocyte surface antigen, putative Tax_Id=36329	126917	Plasmodium falciparum	20.74	2	1	1	21.69	2	1	1				
O96204		PFB0555c Conserved Plasmodium membrane protein Tax_Id=36329	494213	Plasmodium falciparum	20.57	2	1	1	23.09	1	1	1				
Q8I425		PFE0385w Uncharacterized protein Tax_Id=36329	177425	Plasmodium falciparum	20.45	3	1	1	23.25	3	1	1				
Q8I3C9		PFI0090c Putative uncharacterized protein Tax_Id=36329	49467	Plasmodium falciparum	20.31	2	1	1	22.01	2	1	2				

UniProt Accession, Gene Name, Protein Description, Theoretical Molecular Weight (CalcMr), Species, [a]Protein Score, [b]Percent Sequence Coverage, [c]Number of Significant Peptide Matches, [d]Number of Spectral Counts.

Supplementary Table 1: Proteomic analysis of Pf-derived EVs

The table shows the list of (A) human and (B) Plasmodium proteins identified by proteomic analysis.
