# Supplementary material for: 20S proteasomes secreted by the malaria parasite promote its growth
Source: Nat Commun. 2021 Feb 19;12:1172. doi: 10.1038/s41467-021-21344-8 (PMC7895969; doi:10.1038/s41467-021-21344-8)
Supplement: Supplementary file 3 — Description of Additional Supplementary Files [file 41467_2021_21344_MOESM3_ESM.pdf]

Description of Additional Supplementary Files

File Name: Supplementary Data 1
Description: Phosphoproteomics analyses
