# Supplementary material for: 20S proteasomes secreted by the malaria parasite promote its growth
Source: Nat Commun. 2021 Feb 19;12:1172. doi: 10.1038/s41467-021-21344-8 (PMC7895969; doi:10.1038/s41467-021-21344-8)
Supplement: Supplementary file 5 — Reporting Summary [file 41467_2021_21344_MOESM5_ESM.pdf]

nature research

Corresponding author(s): Michal Sharon
Neta Regev-Rudski

Last updated by author(s): Jan 18, 2021

Reporting Summary

Nature Research wishes to improve the reproducibility of the work that we publish. This form provides structure for consistency and transparency in reporting. For further information on Nature Research policies, see our Editorial Policies and the Editorial Policy Checklist.

Statistics

For all statistical analyses, confirm that the following items are present in the figure legend, table legend, main text, or Methods section.

n/a	Confirmed	
☐	☒	The exact sample size (*n*) for each experimental group/condition, given as a discrete number and unit of measurement
☐	☒	A statement on whether measurements were taken from distinct samples or whether the same sample was measured repeatedly
☐	☒	The statistical test(s) used AND whether they are one- or two-sided<br>*Only common tests should be described solely by name; describe more complex techniques in the Methods section.*
☒	☐	A description of all covariates tested
☒	☐	A description of any assumptions or corrections, such as tests of normality and adjustment for multiple comparisons
☐	☒	A full description of the statistical parameters including central tendency (e.g. means) or other basic estimates (e.g. regression coefficient) AND variation (e.g. standard deviation) or associated estimates of uncertainty (e.g. confidence intervals)
☐	☒	For null hypothesis testing, the test statistic (e.g. *F*, *t*, *r*) with confidence intervals, effect sizes, degrees of freedom and *P* value noted<br>*Give P values as exact values whenever suitable.*
☒	☐	For Bayesian analysis, information on the choice of priors and Markov chain Monte Carlo settings
☒	☐	For hierarchical and complex designs, identification of the appropriate level for tests and full reporting of outcomes
☒	☐	Estimates of effect sizes (e.g. Cohen's *d*, Pearson's *r*), indicating how they were calculated

Our web collection on statistics for biologists contains articles on many of the points above.

Software and code

Policy information about availability of computer code

Data collection

Tecan icontrol v. 3.9.1.0
ThermoScientific MyECL Imager v. 2.2.0.1250
JPK Nanowizard III AFM (Berlin, Germany) in QI mode Control Software v. 6.1.159.
Nanoscope v. 9.2 software (Build R2Sr1.130547)
Custom software from the group of Georg Fantner, EPFL, (IbniAFMController-BetaVersion v. 2.0.16-20190117 written under Labview environment)
NIS Element software version 4.4
Dionex Chromatography MS Link (ThermoScientific)
Xcalibur v. 4.2 (ThermoScientific)

Data analysis

JPK data processing software version v. 6.1.86
MaxQuant v. 1.5.2.8.
ImageJ 1.51k, R v.4
NTA v. 2·1 (Nanosight)
MyECL Imager V. 2.2.0.1250 (ThermoScientific)
Gen5™ (BioTek) v. 3.04
Everest (BioRad) v. 2.3.03.0
Amersham Imager 680 (GE Healthcare Life Sciences)
Xcalibur v. 4.2 (ThermoScientific)
Dionex Chromatography MS Link (ThermoScientific)
Percolator v. 2.10

ProteomeDiscoverer v2.1 (Thermo Fisher Scientific)
MSPro and Digger v1.0; http://repository.unimelb.edu.au/10187/18167
Mascot v. 2.4
Gwyddion v. 2.55
Python v. 3.6.8
Scikit-learn v. 0.21.1
Keras v. 2.2.2
SciPy v. 1.2.1
OpenCV v. 3.4.2
R, v. 4.0.3
Origin 8 and the implement algorithm in the OriginPro 2018 data analysis software, v. b9.5.0.193.
OriginLab Pro 2018b (v. 9.55)
GO Ontology database - released 2019-10-08.
PANTHER v. 15 - released 2020-02-14.
Graphpad PRISM v.8
Microsoft Office Excel 2016
Code availability:
The code used for the Acoustic Force Spectroscopy (AFS) cell mechanics experiments can be found in the following link: https://gitlab.com/sorkin.raya/Cellular-mechanics-AFS/tree/master.
The code for image processing and machine learning is composed of many scripts for each sub-task. Relevant scripts will be supplied from the corresponding author by request.

For manuscripts utilizing custom algorithms or software that are central to the research but not yet described in published literature, software must be made available to editors and reviewers. We strongly encourage code deposition in a community repository (e.g. GitHub). See the Nature Research guidelines for submitting code & software for further information.

Data

Policy information about availability of data

All manuscripts must include a data availability statement. This statement should provide the following information, where applicable:
- Accession codes, unique identifiers, or web links for publicly available datasets
- A list of figures that have associated raw data
- A description of any restrictions on data availability

The datasets generated during the current study are available from the corresponding author on reasonable request.
The mass spectrometry phosphoproteomics data have been deposited to the ProteomeXchange Consortium via the PRIDE partner repository with the dataset identifier PXD018154.
The mass spectrometry proteomics data have been deposited to the ProteomeXchange Consortium via the PRIDE [1] partner repository with the dataset identifier PXD023353 and 10.6019/PXD023353

Field-specific reporting

Please select the one below that is the best fit for your research. If you are not sure, read the appropriate sections before making your selection.

☒ Life sciences ☐ Behavioural & social sciences ☐ Ecological, evolutionary & environmental sciences

For a reference copy of the document with all sections, see nature.com/documents/nr-reporting-summary-flat.pdf

Life sciences study design

All studies must disclose on these points even when the disclosure is negative.

Sample size	We usually used a sample size of three replicates per treatment, based on our previous experience with similar experiments. In some biological experiments, and dependent on the biological material availability, we increased sample size to increase the statistical power.
Data exclusions	Data were only excluded if the experiment failed for technical reasons.
Replication	In vitro activity assays were performed three times, with successful replication noted for all experiments.<br>Cellular experiments were performed between three and twelve times, with successful replication noted for all experiments.
Randomization	The selection of which cells will receive what treatment, in all experiments, was random.
Blinding	All measurements are machine-based and not human assessments, therefore blinding is not relevant to this study.

Reporting for specific materials, systems and methods

We require information from authors about some types of materials, experimental systems and methods used in many studies. Here, indicate whether each material, system or method listed is relevant to your study. If you are not sure if a list item applies to your research, read the appropriate section before selecting a response.

Materials & experimental systems

n/a	Involved in the study
☐	☒ Antibodies
☒	☐ Eukaryotic cell lines
☒	☐ Palaeontology and archaeology
☒	☐ Animals and other organisms
☐	☒ Human research participants
☒	☐ Clinical data
☒	☐ Dual use research of concern

Methods

n/a	Involved in the study
☒	☐ ChIP-seq
☐	☒ Flow cytometry
☒	☐ MRI-based neuroimaging

Antibodies

Antibodies used	anti-PSMD1 (ab2941, Abcam), anti-PSMA1 (ab140499, Abcam), HSP90 (ab13495, Abcam), Dematin (ab226357, Abcam), GAPDH (ab8245, Abcam), Spectrin alfa chain (SPTA1) (ab11751, Abcam), anti- SR1 (ab71893, Abcam), anti-beta adducin (ADD2) (ab251821, abcam), anti-protein 4.1r (EPB41) (ab185704, Abcam), anti-- Ankyrin (ANK1) (ab212053, Abcam), anti-Phospho-serine threonine (ab17464, Abcam), anti ubiquitin (PW0930, Enzo).<br>Secondary antibodies used for western blots include Goat anti-mouse IgG-HRP (115-035-003, Jackson) and Goat anti-rabbit IgG-HRP (111-035-003, Jackson).
Validation	All antibodies were validated by the supplier. Datasheets of all antibodies contain a representative western blot example. They were further validated in the lab by Western blot using appropriate cell lysates.

Human research participants

Policy information about studies involving human research participants

Population characteristics	Not relevant in this study
Recruitment	The malaria parasites were grown at human A+ RBCs from naïve donors supply by the Israeli “MDA” blood bank
Ethics oversight	The use of human blood was approved at the by the IRB and Helsinki committees, and signed by the chairs, Prof. Yosef Shaul (IRB) and Prof. Arnon Afek (Helsinki). Approval documents are included.

Note that full information on the approval of the study protocol must also be provided in the manuscript.

Flow Cytometry

Plots

Confirm that:

- ☒ The axis labels state the marker and fluorochrome used (e.g. CD4-FITC).
- ☒ The axis scales are clearly visible. Include numbers along axes only for bottom left plot of group (a 'group' is an analysis of identical markers).
- ☒ All plots are contour plots with outliers or pseudocolor plots.
- ☒ A numerical value for number of cells or percentage (with statistics) is provided.

Methodology

Sample preparation	Live cells were stained with HO and TO and incubated for 30min at 37deg, then processed in ZE5.
Instrument	Biorad ZE5 cell analyzer
Software	FCS express v7
Cell population abundance	In the FACS experiments we use only packed, uninfected red blood cells, which is 100% homogeneous, therefore, cell population abundance is not relevant for these experiments.
Gating strategy	Gating strategy was done according to the uRBCs control (that does not express HO and TO).

☒ Tick this box to confirm that a figure exemplifying the gating strategy is provided in the Supplementary Information.
