# Supplementary material for: 20S proteasomes secreted by the malaria parasite promote its growth
Source: Nat Commun. 2021 Feb 19;12:1172. doi: 10.1038/s41467-021-21344-8 (PMC7895969; doi:10.1038/s41467-021-21344-8)

Supplementary Figure S2 – Raw data for Fig 1A, 1B

A. % parasitemia relative to control for Fig. 1A

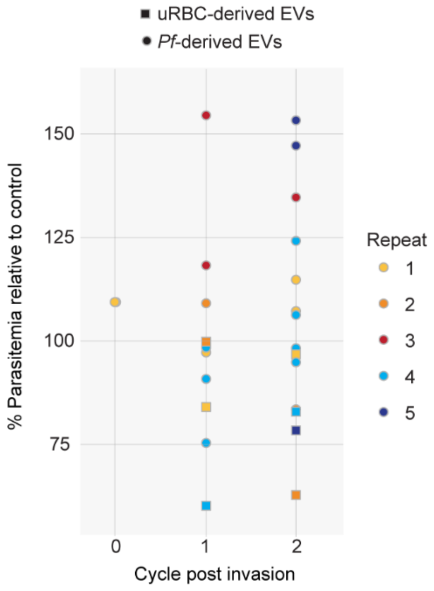

B. % parasitemia for Fig. 1A

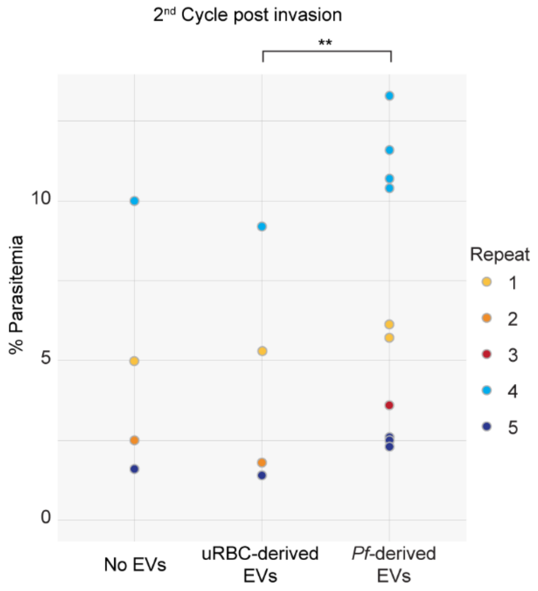

C. % parasitemia relative to control for Fig. 1B

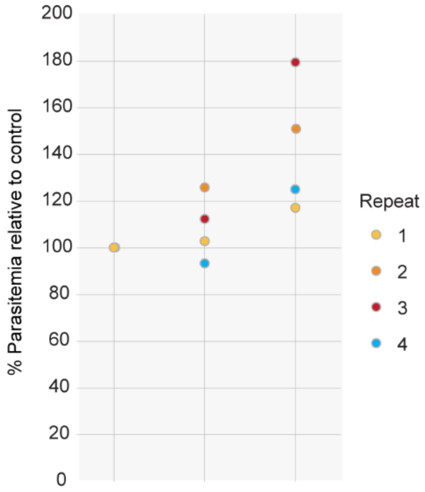

D. % parasitemia for Fig. 1B

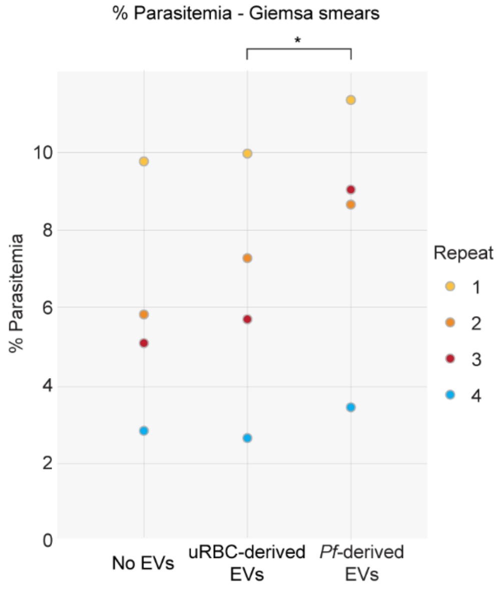

Original blots for fig. 3A

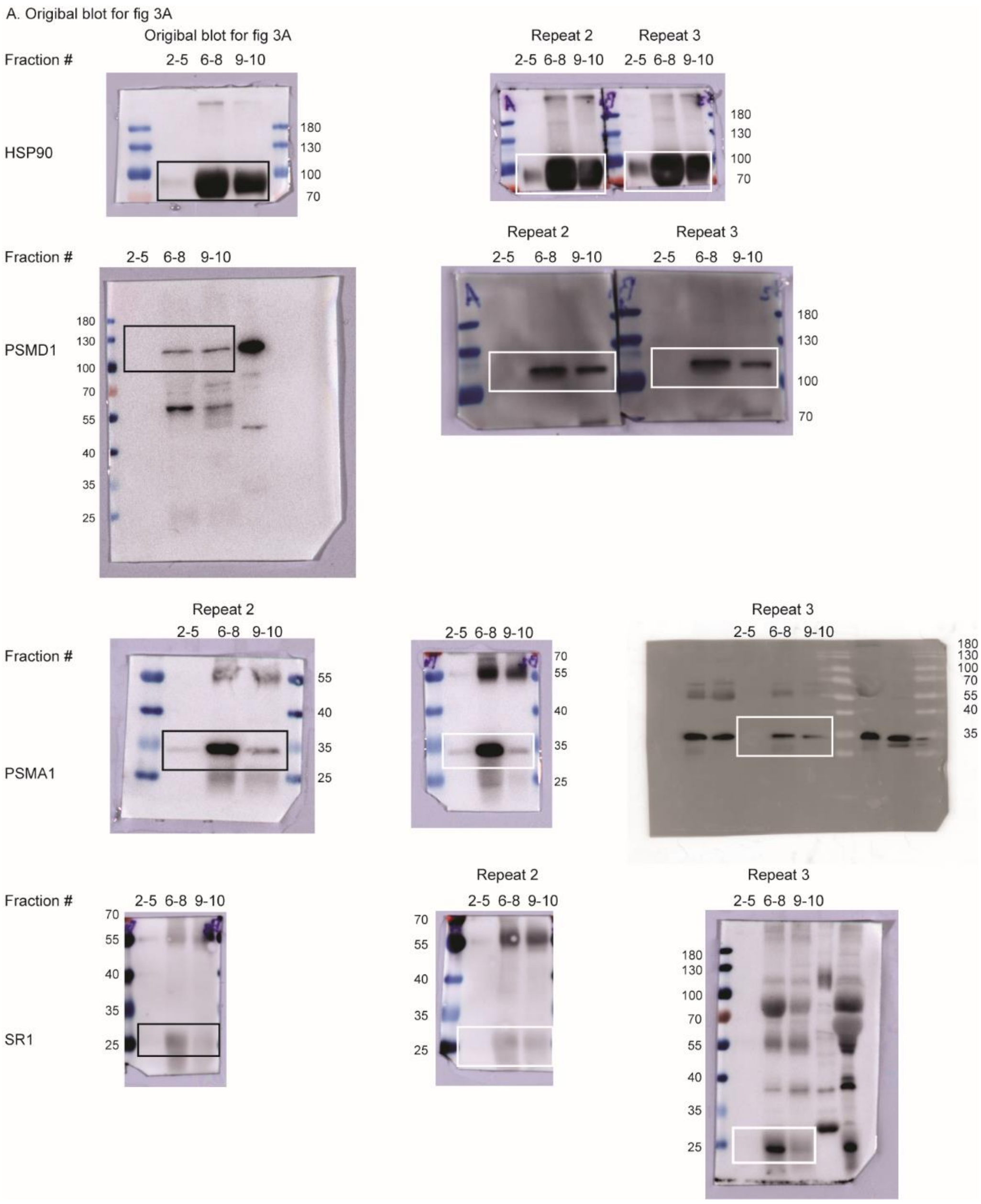

Original gels and blots for fig. 3C

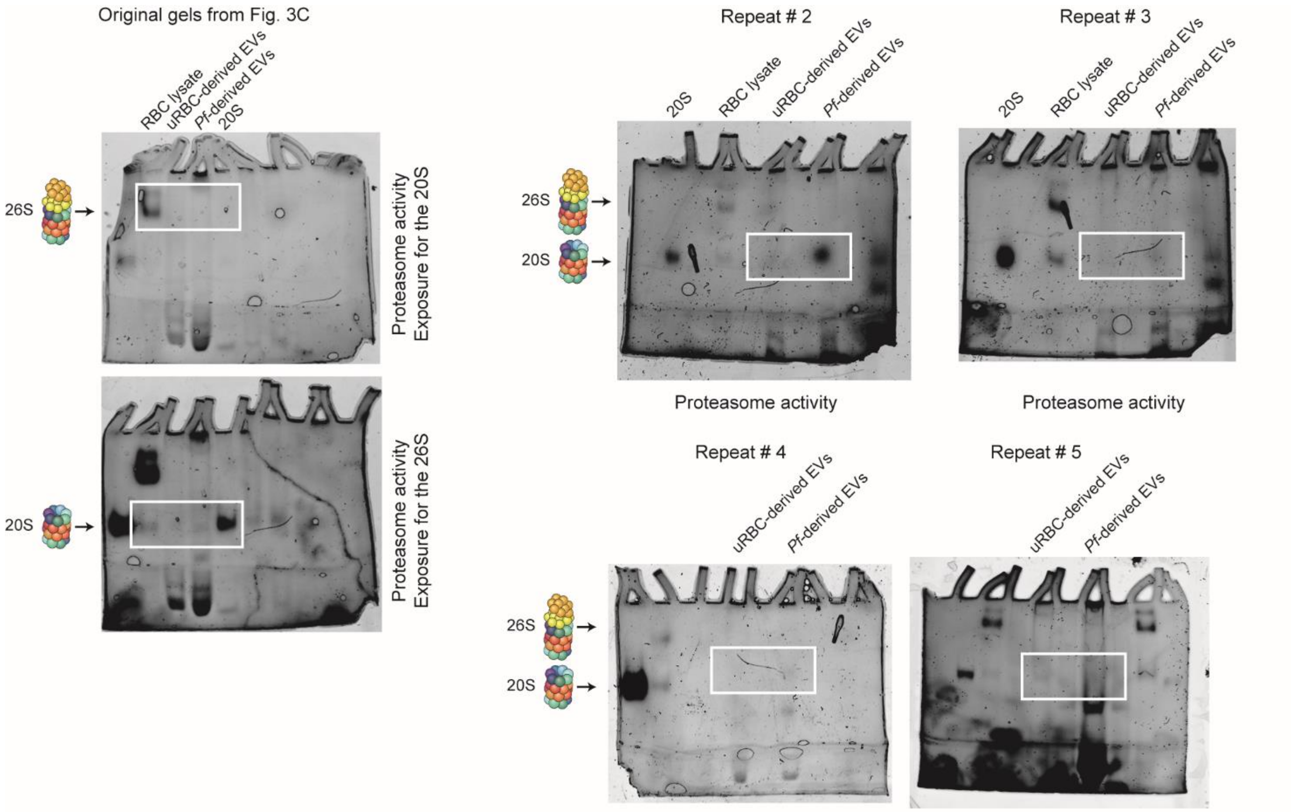

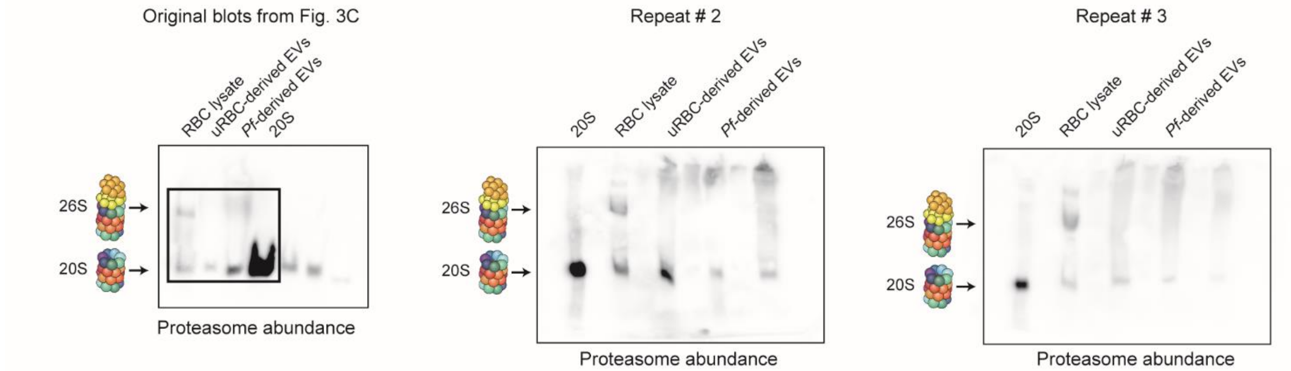

Original gels and blots for fig. 3D

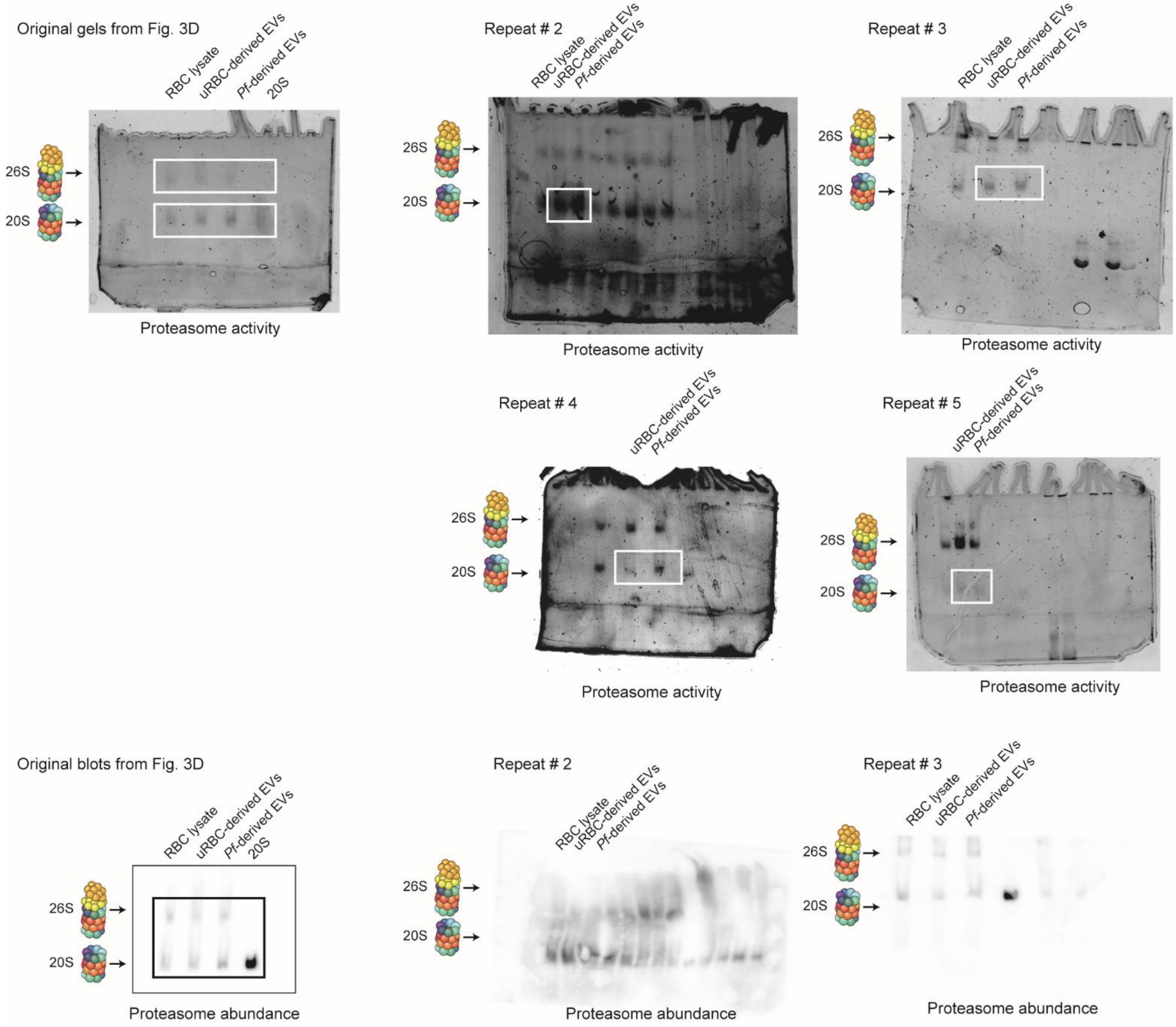

Original blots for fig. 3E

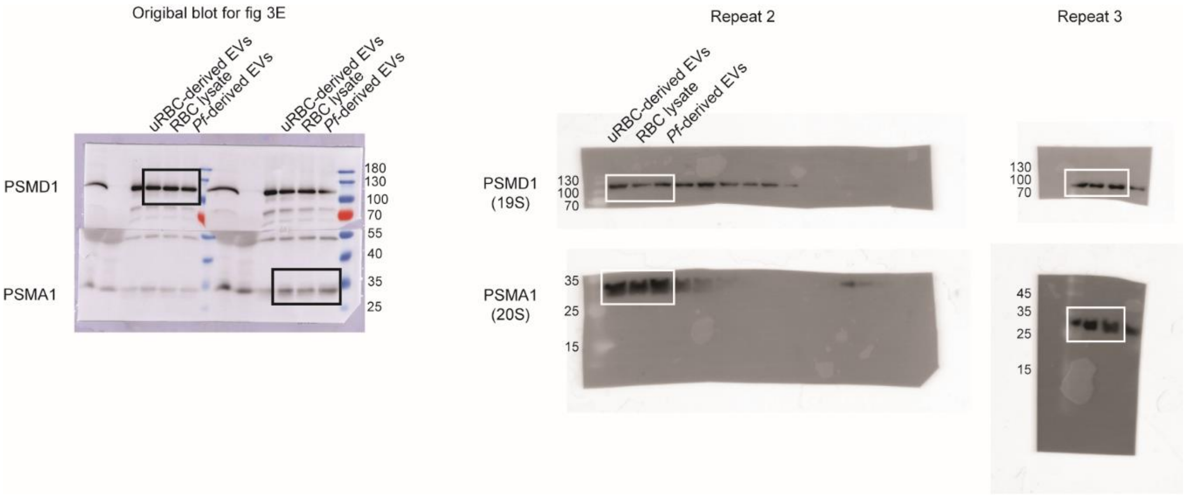

Supplementary Figure S7 – Raw data for Fig. 3B, 3C, 3D, 3F

A. Raw data for Fig. 3B

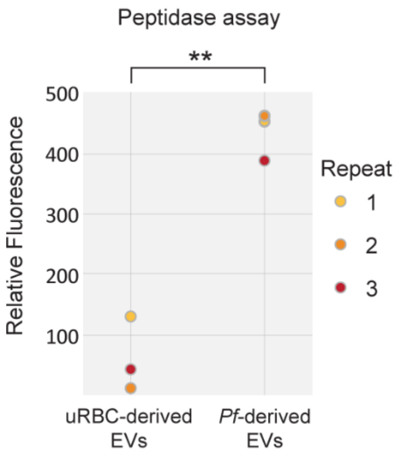

B. Raw data for Fig. 3C

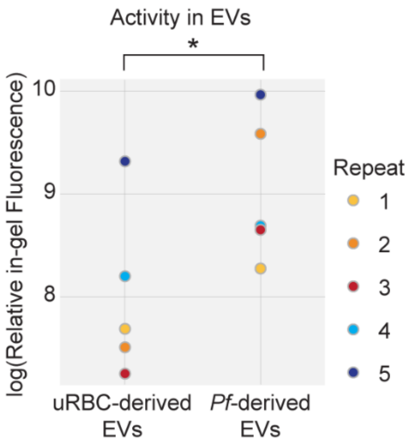

C. Raw data for Fig. 3D

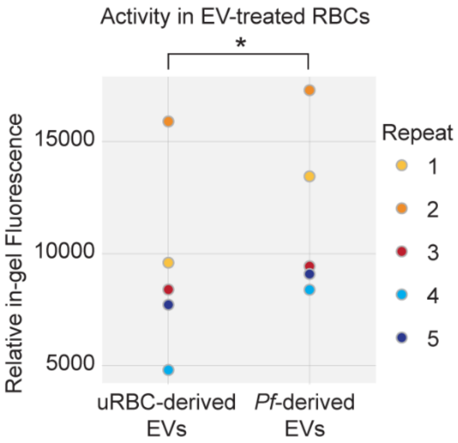

D. Raw data for Fig. 3F

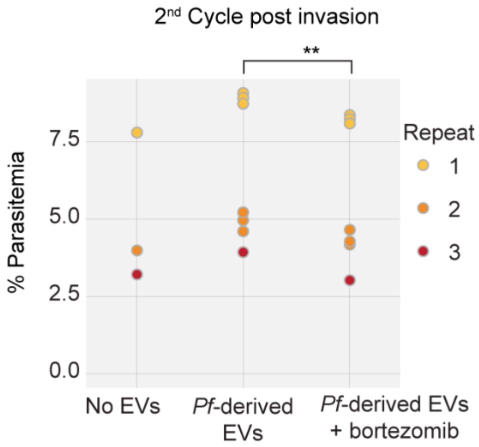

Original blots for fig. 5A

Figure 5A - original gels

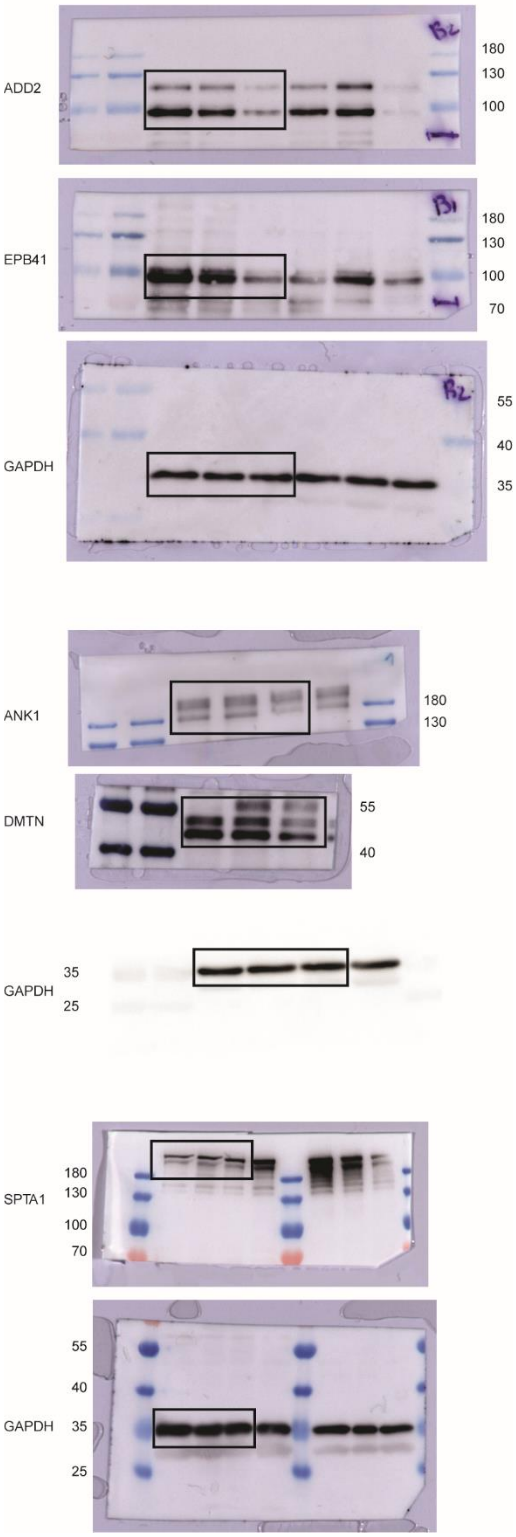

Original blots for fig. 5D

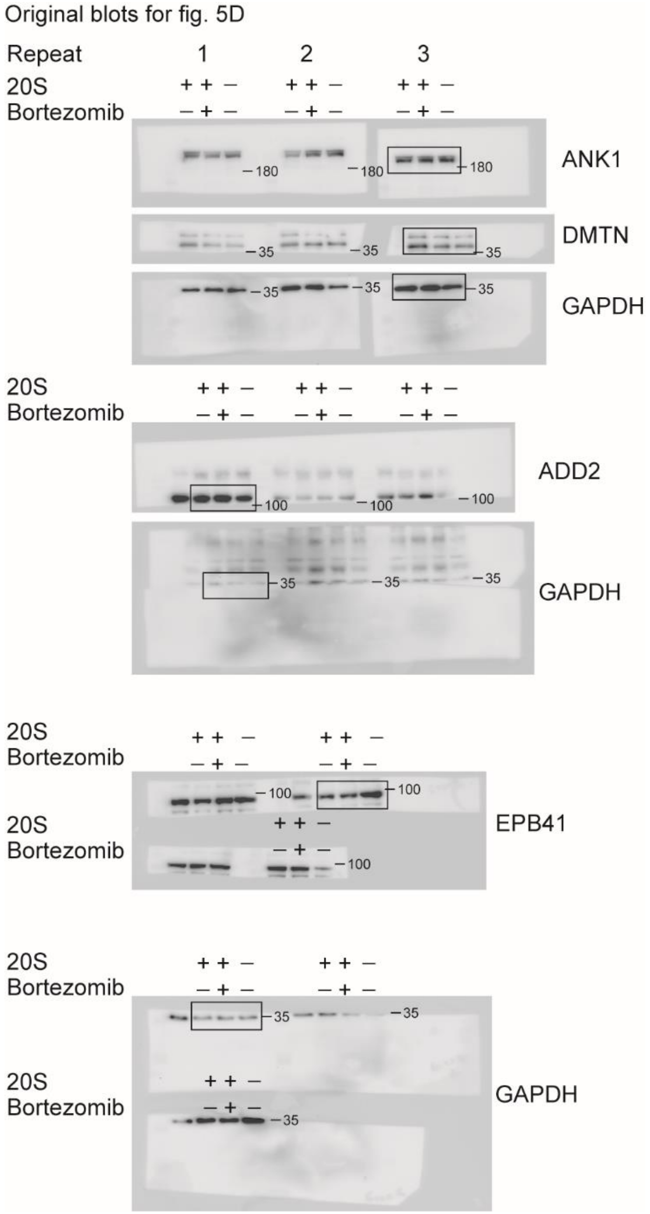

Original blots for fig. 5E

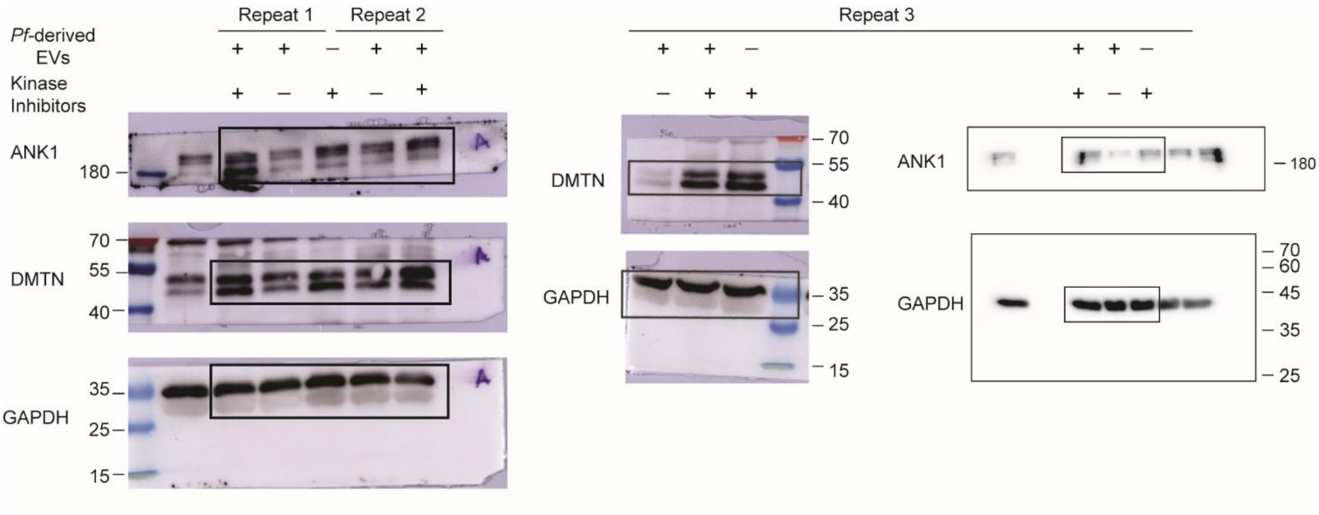

Supplementary Figure S14 – Raw data for fig. 5B, 5F

A. Raw data for Fig. 5B

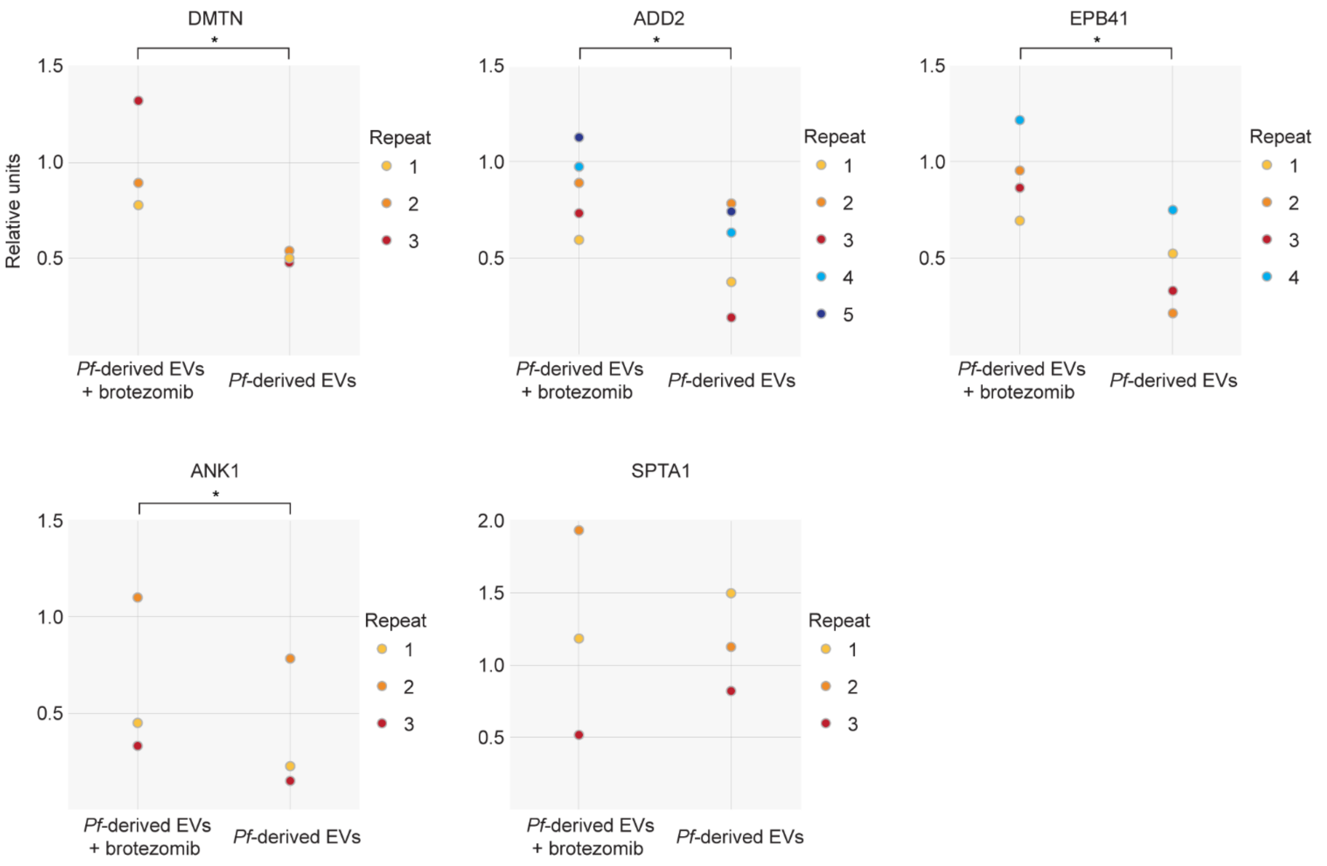

B. Raw data for Fig. 5F

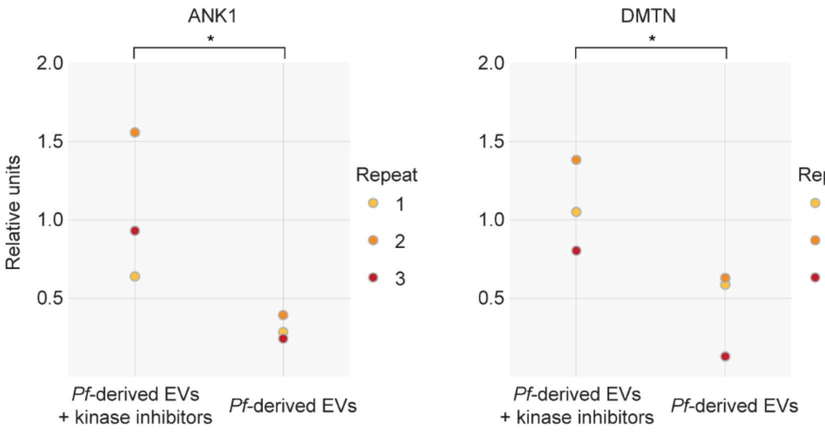

Original blots for 3 repeats of fig. S5

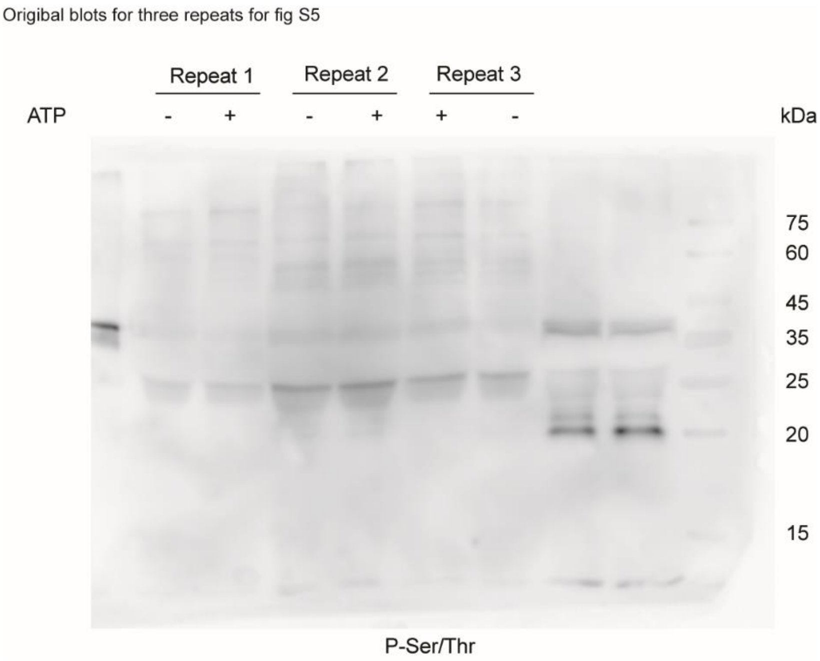

Original blots for 2 repeats for fig. S6A

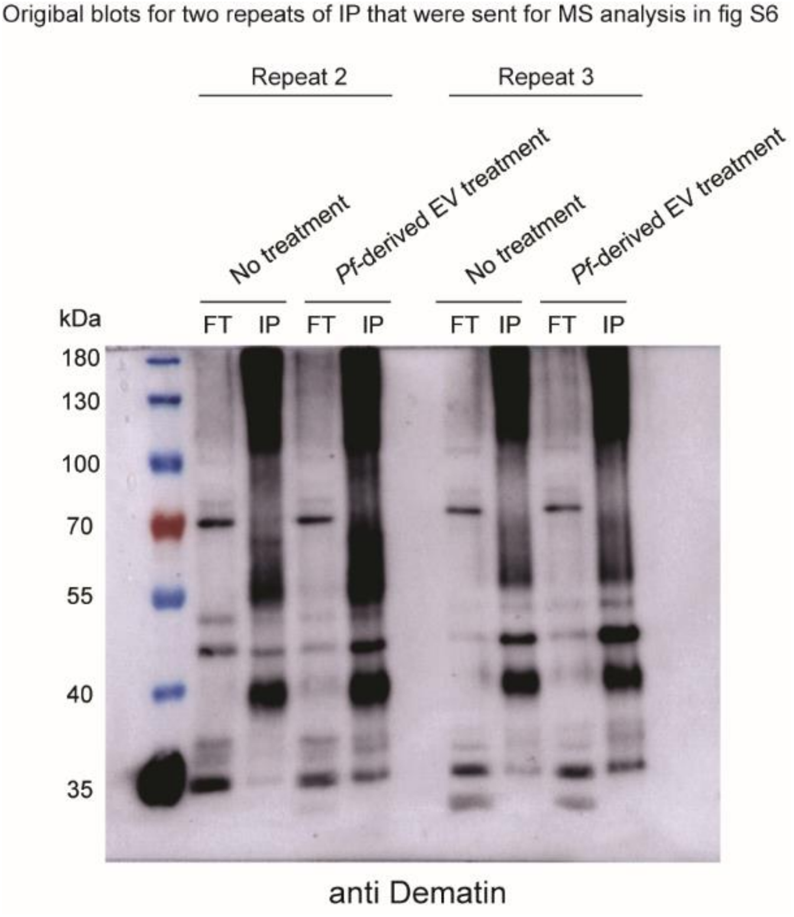

Original blots for 3 repeats of fig. S10

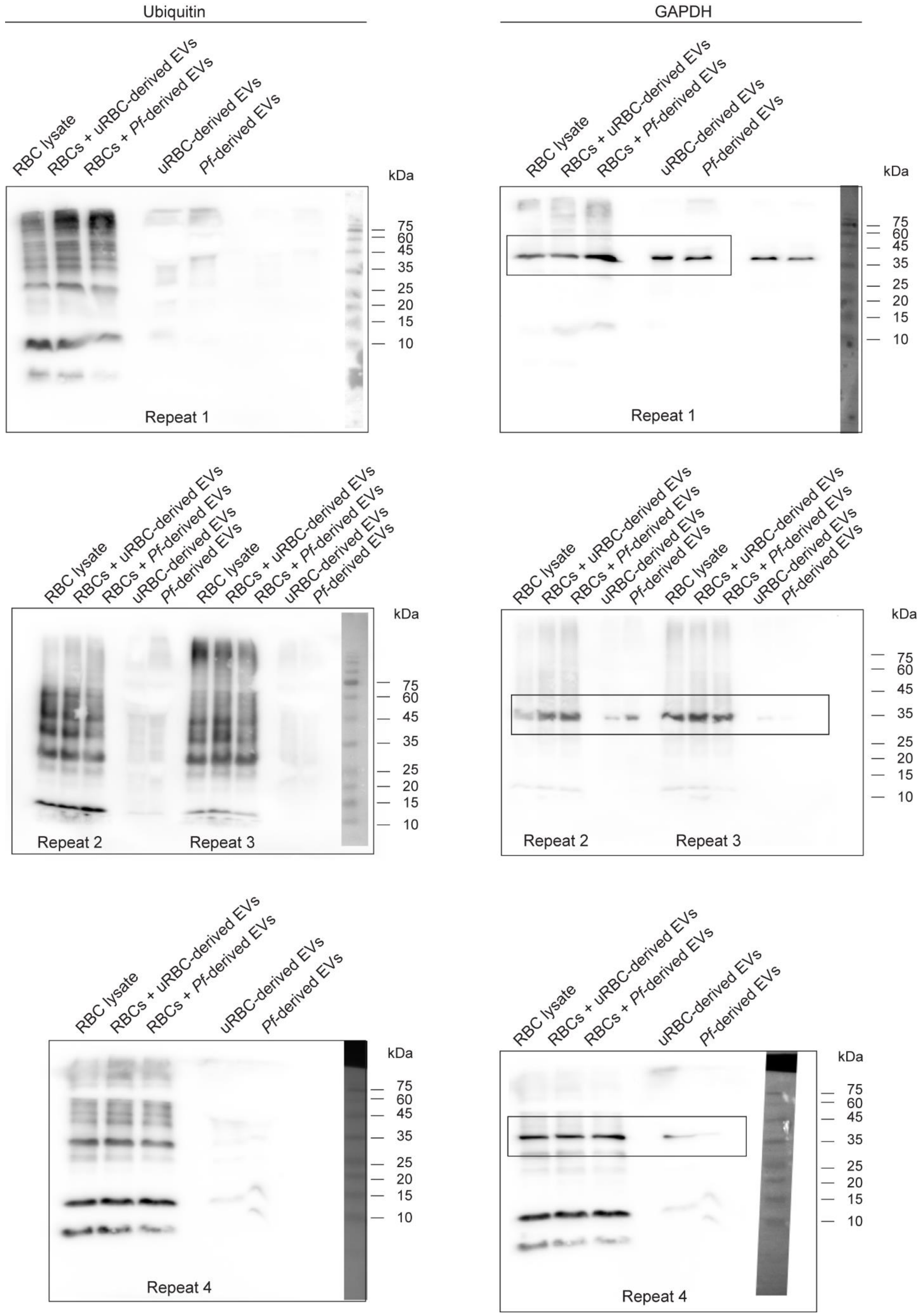

Supplement: Supplementary file 6 — Source Data [file 41467_2021_21344_MOESM6_ESM.zip › Source Data/Source Data_Gels and Blots.pdf]
